# Rhizosphere Microbiomes in a Historical Maize-Soybean Rotation System Respond to Host Species and Nitrogen Fertilization at the Genus and Subgenus Levels

**DOI:** 10.1128/AEM.03132-20

**Published:** 2021-05-26

**Authors:** Michael A. Meier, Martha G. Lopez-Guerrero, Ming Guo, Marty R. Schmer, Joshua R. Herr, James C. Schnable, James R. Alfano, Jinliang Yang

**Affiliations:** a Department of Agronomy and Horticulture, University of Nebraska—Lincoln, Lincoln, Nebraska, USA; b Center for Plant Science Innovation, University of Nebraska—Lincoln, Lincoln, Nebraska, USA; c Department of Biochemistry, University of Nebraska—Lincoln, Lincoln, Nebraska, USA; d USDA-ARS Agroecosystem Management Research Unit, Lincoln, Nebraska, USA; e Department of Plant Pathology, University of Nebraska—Lincoln, Lincoln, Nebraska, USA; Chinese Academy of Sciences

**Keywords:** 16S, ASV, amplicon sequence variants, crop rotation, maize, microbiome, nitrogen fertilization, soybean, rhizobiome, rhizosphere-inhabiting microbes

## Abstract

Root-associated microbes are key players in plant health, disease resistance, and nitrogen (N) use efficiency. It remains largely unclear how the interplay of biological and environmental factors affects rhizobiome dynamics in agricultural systems. In this study, we quantified the composition of rhizosphere and bulk soil microbial communities associated with maize (Zea mays L.) and soybean (Glycine max L.) in a long-term crop rotation study under conventional fertilization and low-N regimes. Over two growing seasons, we evaluated the effects of environmental conditions and several treatment factors on the abundance of rhizosphere- and soil-colonizing microbial taxa. Time of sampling, host plant species, and N fertilization had major effects on microbiomes, while no effect of crop rotation was observed. Using variance partitioning as well as 16S sequence information, we further defined a set of 82 microbial genera and functional taxonomic groups at the subgenus level that show distinct responses to treatment factors. We identified taxa that are highly specific to either maize or soybean rhizospheres, as well as taxa that are sensitive to N fertilization in plant rhizospheres and bulk soil. This study provides insights to harness the full potential of soil microbes in maize and soybean agricultural systems through plant breeding and field management.

**IMPORTANCE** Plant roots are colonized by large numbers of microbes, some of which may help the plant acquire nutrients and fight diseases. Our study contributes to a better understanding of root-colonizing microbes in the widespread and economically important maize-soybean crop rotation system. The long-term goal of this research is to optimize crop plant varieties and field management to create the best possible conditions for beneficial plant-microbe interactions to occur. These beneficial microbes may be harnessed to sustainably reduce dependency on pesticides and industrial fertilizer. We identify groups of microbes specific to the maize or to the soybean host and microbes that are sensitive to nitrogen fertilization. These microbes represent candidates that may be influenced through plant breeding or field management, and future research will be directed toward elucidating their roles in plant health and nitrogen usage.

## INTRODUCTION

Crop rotations of maize and soybean exploit the symbiotic relationship of legumes with nitrogen (N)-fixing bacteria. This rotation system has historically been a widespread practice in the United States and continues to be employed as a supplement to synthetic N fertilizer (1). Soybean-maize (2) and other crop rotations in general (3, 4) have also shown beneficial effects on crop yield, disease resistance, weed management, and soil nutrient conservation. Root-colonizing soil microbes may play a role in N use efficiency (5), plant health (6), and crop performance (7) in agricultural fields. Furthermore, the capacity of plants to recruit a specific set of beneficial microbes can potentially be employed in plant breeding and genetic engineering to improve disease resistance and yield potential of crop plants while reducing the application of exogenous fertilizer and pesticides (8–11).

Soil and rhizosphere microbial communities have been studied for several major crop species, including maize (12), soybean (13), wheat (14), and rice (15), as well as in crop rotation systems, including maize-wheat (16), wheat-maize-soybean (17), and more complex systems (4). Similarly, the effects of N fertilization on microbial communities have been studied in maize (18), wheat (19), and rice (20). Previous studies have also shown that soil microbial communities are sensitive to farming practices such as conventional versus organic, tillage versus no tillage, or cover crop versus fallow fields (21, 22). Overall, a large body of research has shown that crop plant species, N fertilization, and possibly crop rotation affect rhizosphere microbial community structure. However, it is largely unknown how combinations of these factors together shape rhizosphere and soil microbial communities and how each factor ranks in terms of its impact on the abundance of distinct rhizosphere- and soil-colonizing microbial taxa. For instance, it has been unclear whether maize and soybean planted in succession in the same field would adopt similar root microbiomes in response to soil “memory” induced by the previous year’s crop (23), or if the effect of the host plant would outweigh any crop rotation effects.

In this study, we leveraged a long-term experimental field (1, 24) with consistent crop rotations (established 1972) and N fertilizer regimes (established 1983) in a 2-year replicated experiment. Through 16S sequencing of rhizosphere and bulk soil samples and statistical modeling of individual amplicon sequence variants (ASVs), we aim to rank the impact of agriculturally relevant factors, including environmental conditions (year and month of sampling), biological factors (crop plant species), and agricultural practices (N fertilization and crop rotation) on the abundance of rhizosphere- and bulk soil-colonizing microbes. We further aim to identify functional taxonomic groups of microbes that respond to these diverse treatment factors as consistent units. Among these taxa, we seek to identify the key respondents that are specific to either maize or soybean and taxa that respond to inorganic N fertilization or the lack thereof.

## RESULTS

Overall, sequencing yielded 41.4 million (41.4M) raw 16S reads for 384 samples, with a median number of 121,000 (121K) reads per sample for rhizosphere and 103K reads per sample for bulk soil samples. After a series of quality and abundance filtering steps (see Fig. S1 in the supplemental material), a final set of 4.3M reads were retained that belong to a curated set of 2,225 unique ASVs derived from both rhizosphere and bulk soil samples. The median read counts per sample were 13.1K for rhizosphere and 5.9K for bulk soil samples.

### Rhizosphere and soil microbiomes in a historical crop rotation system are highly dynamic over time and across niche environments.

**FIG 1:**
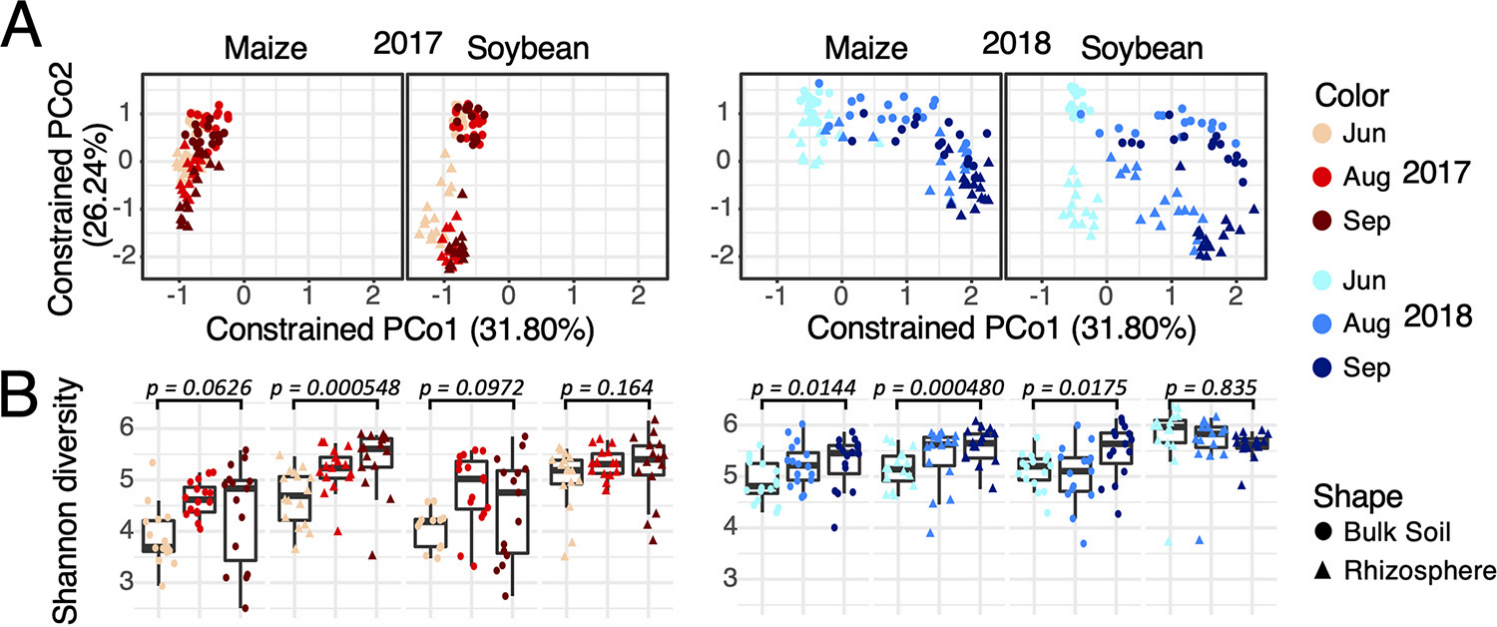
Principal-coordinate analysis (PCoA) identifies time of sampling and soil compartments as major factors shaping high-level microbial community structure as measured by Shannon diversity index. (A) PCoA using weighted UniFrac distances, separated into four panels by year and plant species. Colors indicate sampling time points, and shapes indicate soil compartments. (B) Shannon diversity index plotted for each sample and summarized in box plots grouped by plant species, soil compartment and month. *P* values are given for comparison between early season (June) and late season (September) samples using one-tailed *t* tests.

Because our field experiments are subject to year and seasonal effects (Fig. S2), our first analysis was to assess how rhizosphere and bulk soil microbiomes vary across early, middle, and late season sampling time points in two consecutive years. Constrained principal-coordinate analysis (PCoA) (Fig. 1A) revealed the time of sampling to be the largest source of variation (PCoA axis 1, 31.80%), followed by soil compartment rhizosphere versus bulk soil (PCoA axis 2, 26.24%), while effects of host plant species and N treatment were also apparent (Fig. S3). Time point variation may be attributable to temperature and precipitation patterns. In particular, the last sampling time point in 2018 occurred soon after a major precipitation event associated with drastic changes in microbial community composition (Fig. S2). Rhizosphere and bulk soil microbiomes are more dissimilar in soybean than in maize, with clear separation along axis 2 in the PCoA plot. In both soil compartments, we observed higher microbial diversity in 2018 than in 2017 as measured by the Shannon diversity index (Fig. 1B). In addition, both bulk soil and rhizosphere microbiomes tended to increase in diversity as the season progressed (Fig. 1B).

### Environment, host plant, and agricultural practice together shape microbial communities.

**FIG 2:**
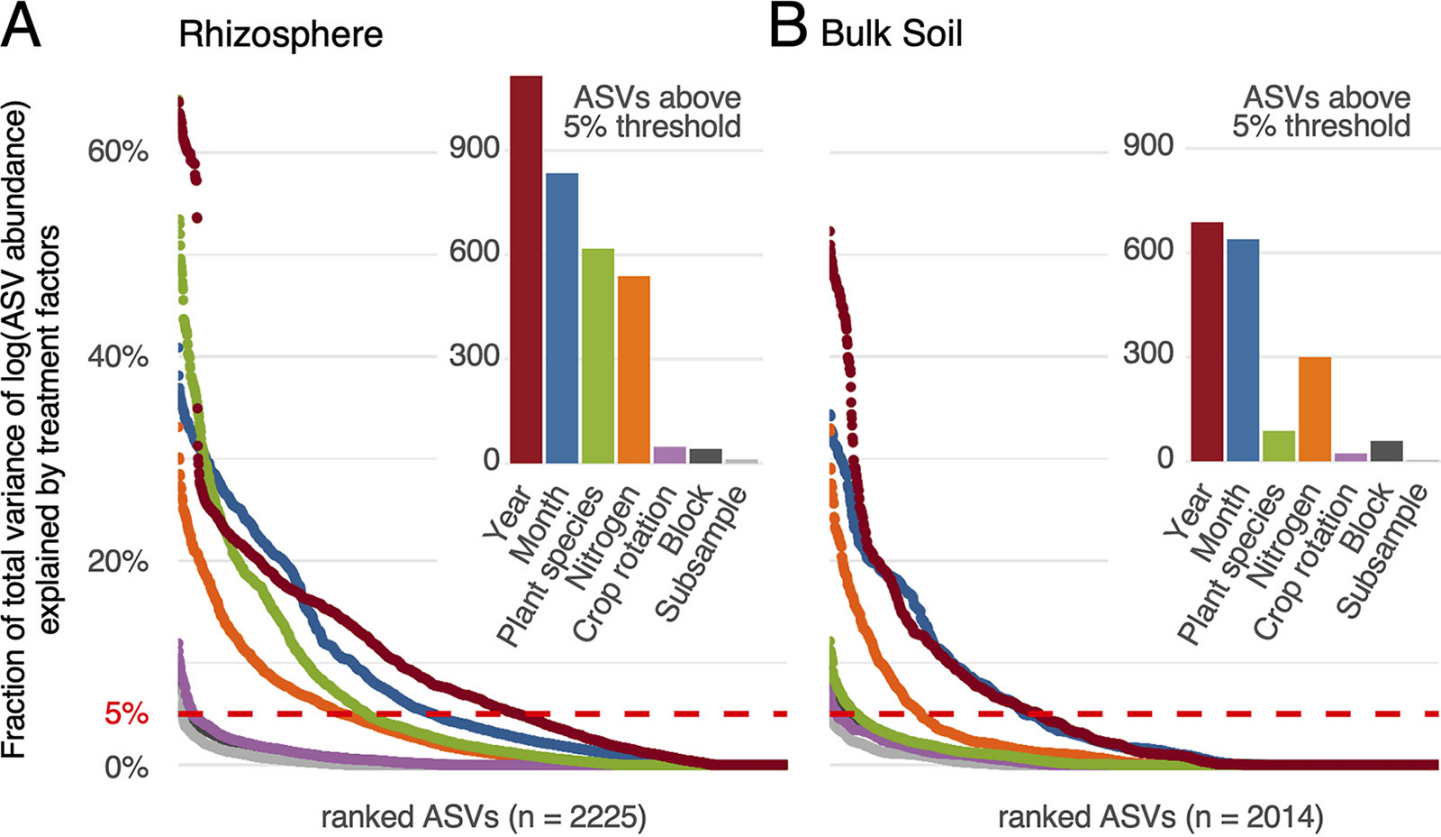
Variance partitioning results for different treatment factors influencing ASV variation in rhizosphere and bulk soil samples. For each treatment factor, percent variance explained (*y* axis) was calculated for ASVs in the rhizosphere (A) and in bulk soil (B). ASVs were ranked by response to treatment factors (*x* axis). Red dashed lines indicate the 5% arbitrary threshold. The inset figures show the numbers of ASVs exceeding the 5% arbitrary threshold for different treatment factors.

We fit a mixed linear model for each ASV as a response variable to reveal in more detail to what degree microbial communities are influenced by different treatment factors (see Materials and Methods). Through variance partitioning, we calculated the proportion of total variance attributable to each treatment factor (termed “variance scores”) for rhizosphere (2,225 ASVs) and bulk soil (2,014 ASVs). We tallied the number of ASVs that are responsive to treatment—defined here as any ASVs with a variance score above an arbitrary threshold of 5%—to estimate the relative importance of each treatment factor in shaping microbiome composition (Fig. 2). For rhizosphere data, out of *n* = 2,225 ASVs, we found 1,115 (50.1%) responsive to year and 835 (37.5%) responsive to month above the 5% threshold. For bulk soil data, out of *n* = 2,014 ASVs, we found 668 (34.2%) responsive to year and 639 (31.7%) responsive to month. These results agreed with our previous observations (Fig. 2) and suggested that environmental factors affect microbiome abundance in the rhizosphere more than in bulk soil.

Interestingly, microbial communities responded to host plant species to a statistically significantly higher degree in the rhizosphere than the bulk soil (chi-square *P* value = 2.2e−16), with variance scores of 618 ASVs in rhizosphere and only 88 ASVs in bulk soil exceeding 5%. Employing a threshold of 10% reveals a similar pattern with 422 ASVs in the rhizosphere and 9 ASVs in bulk soil exceeding the threshold (chi-square *P* value = 2.2e−16), and patterns were overall consistent at thresholds of 2.5% or 10% (Fig. S4). For 36 ASVs in the rhizosphere, more than 40% of total variance was explained by host plant species, whereas no response was observed in bulk soil. These results are consistent with the idea that rhizosphere ecosystems are home to highly specialized microbes that have coevolved alongside plant hosts, whereas bulk soil harbors more uniform microbial communities.

Among factors related to agricultural practice, we found that 5% or more variability was explained by N treatment in 539 rhizosphere ASVs and in 300 bulk soil ASVs (chi-square *P* value = 3.6e−14), with scores exceeding 20% for 71 and 42 ASVs (chi-square *P* value = 0.03267), respectively. In contrast, response to crop rotation was negligible in both rhizosphere and bulk soil, suggesting that the previous year’s crop has at best a minor effect on microbial community composition in any given year. We detected no noticeable variation due to experimental blocks and subsamples.

### Response to experimental treatments reveals functional groups of microbial taxa at subgenus level.

As responses to host plant species and N treatment were apparent at the level of individual ASVs, we hypothesized that the responsive ASVs might be clustered into functional taxonomic groups. To address this hypothesis, we binned ASVs into 87 distinct microbial genera based on SILVA taxonomy annotation. However, by plotting all ASVs within each genus against the variance scores in response to plant host species and N treatment, we noticed a high range of values in some cases (Fig. S5), suggesting that there may be distinct groups of ASVs within the same genus that show different responses to treatments.

To achieve taxonomic resolution beyond the genus level, a phylogenetic tree of all ASVs was plotted together with the variance scores for each of 87 genera. This procedure allowed us to identify a total of 105 genera and subgenus groups that show distinct and unambiguous responses to treatments (here indicated with the suffix “_S”). Using this approach, we identified subgroups in 12 genera: *Streptomyces*, *Chitinophaga*, *Flavobacterium*, *Pedobacter*, *Mucilaginibacter*, *Burkholderia*, *Pseudomonas*, *Sphingomonas*, *Sphingobium*, *Mesorhizobium*, *Nitrobacter*, and *RB41* with distinct patterns of variance partitioning (Supplemental File S1). We refer to these groups as subgenus groups to draw a distinction between functional groups identified by their response to treatment variables and microbial species that are categorized through 16S rRNA phylogeny.

**FIG 3:**
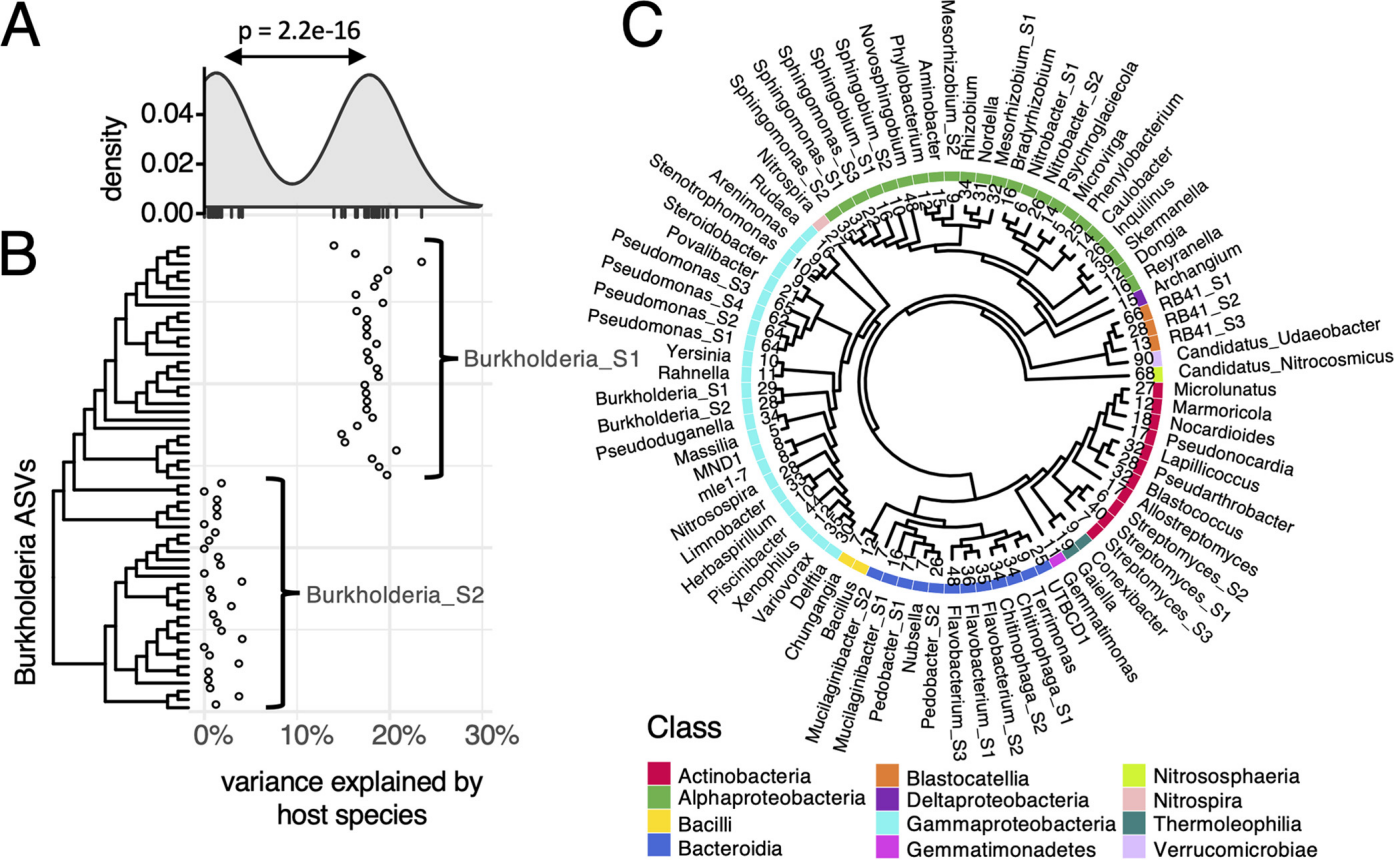
A set of 82 taxonomic groups at the genus and subgenus level was defined based on 16S rRNA sequences and response to treatment factors. (A) Variance explained by host species in rhizospheres plotted for 57 ASVs in the genus *Burkholderia*. The density plot indicates bimodal distribution. (B) Variance scores plotted against the phylogenetic tree of all ASVs in the *Burkholderia* genus reveal subgenus groups *Burkholderia_S1*, which responds to host plant species, and *Burkholderia_S2*, which is indifferent to host plant species. (C) Phylogeny of 82 taxonomic groups analyzed in this study. Numbers above cladogram tips indicate the number of unique ASVs observed in each taxonomic group. Colors indicate class, and tip labels indicate genus and subgenus group (S) where applicable.

For example, the genus *Burkholderia* (Fig. 3A and B) shows two clusters of ASVs (*Burkholderia_S1*, *n* = 29 ASVs, and *Burkholderia_S2*, *n* = 28 ASVs) that exhibited significantly different variance scores (Wilcoxon rank sum test *P* value = 2.2e−16). These clusters are further grouped by phylogeny, which may indicate separate evolutionary lineages. Notably, the same subgenus groups can be distinguished based on total abundance. *Burkholderia_S1* ASVs are overall more abundant than *Burkholderia_S2* ASVs (Fig. S6). Furthermore, 52 out of 57 *Burkholderia* ASVs were recovered in two independent external data sets and the same subgenus groups were observed in different geographical locations and at different times (Fig. S7). This increases our confidence that experimental data can be used in conjunction with 16S sequence information to distinguish functional taxonomic groups at the subgenus level and that the same subgenus groups are reproducible in similar experiments.

In total, a final set of 82 taxonomic groups (genera and subgenus groups) was defined that responded to treatments as a unit. These groups spanned 64 genera and 12 classes of prokaryotes and contained between 5 and 102 ASVs, displayed in a phylogenetic tree (Fig. 3C) generated based on 300-bp 16S sequences and rooted using the outgroup *Candidatus_Nitrocosmicus* (*Archaea*). This set of 82 taxa was used for subsequent analyses in this study. Total abundances of each group were estimated by the sum of read counts across all samples (Fig. S6).

To evaluate how our ASV grouping method compares to automated operational taxonomic unit (OTU) clustering, OTU picking was performed on the sets of ASVs within each of the 12 genera for which we identified subgenus groups (see Materials and Methods). The number of subgenus groups generated by classical OTU picking at a fixed 97% sequence identity threshold was in many cases larger than the number of subgroups identified using our method, which may indicate some redundancy (Table S1). In other cases (including *Burkholderia* in Fig. 3A and B), OTU picking failed to identify subgenus groups altogether, even though variance partitioning data show a clear distinction in the functional behavior of groups of ASVs.

Clustering ASVs into functional groups that respond consistently to treatments allowed us to identify specific taxa that differ between environmental and experimental conditions discussed above (Fig. 2) at the highest possible taxonomic resolution.

### Maize and soybean recruit distinct and highly specialized microbial taxa to rhizospheres.

**FIG 4:**
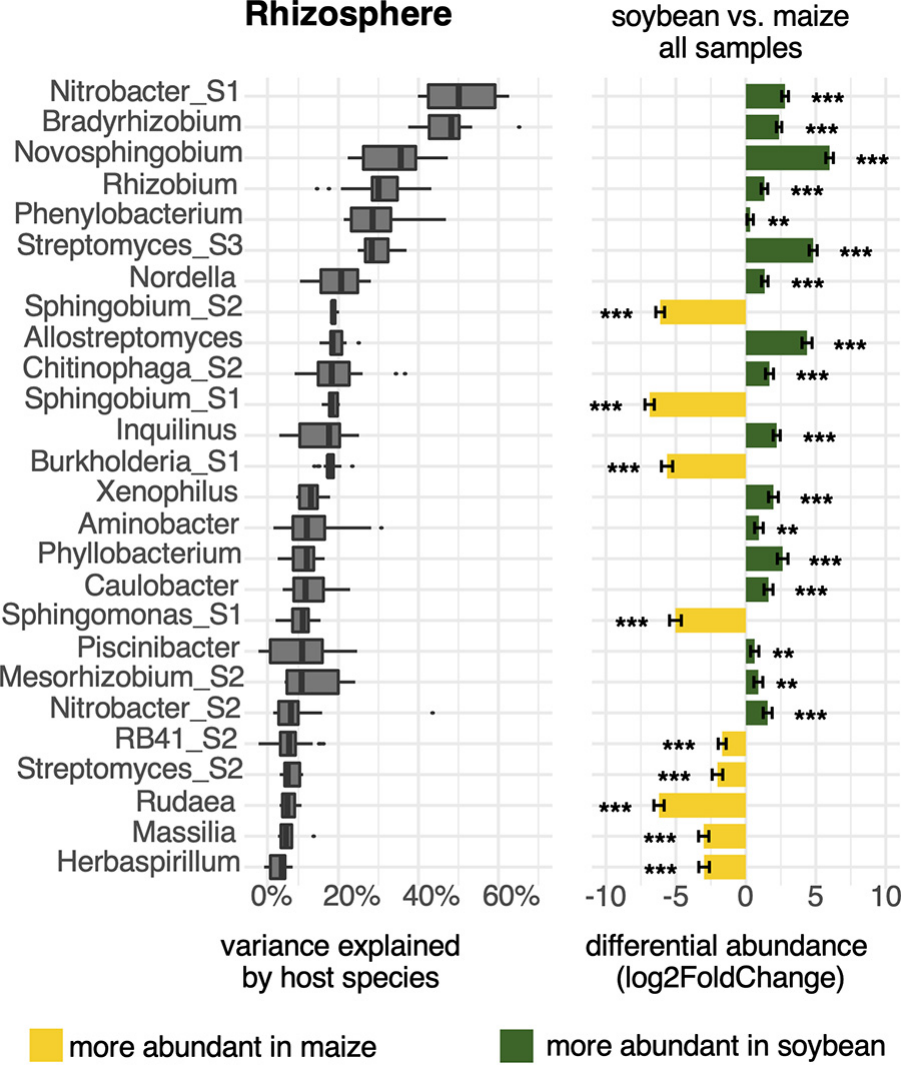
Several microbial groups are enriched in either maize or soybean rhizospheres. Taxonomic groups at the genus and subgenus levels were ranked by the fraction of total variance explained by the host plant species (left). Groups with a median variance score >5% are shown. Differential abundance of groups, log_2_ (abundance in soybean/abundance in maize), was calculated for 96 pairs of rhizosphere samples using DESeq2 (right). Bars show standard errors, and asterisks indicate significantly different abundance between soybean and maize at FDR-adjusted *P* values of <0.01 (***) and <0.05 (**).

Twenty-six rhizosphere-dwelling taxa showed a strong response to host plant species (Fig. 4). Out of the top 10 taxa responding to host plant species, 9 are specific to soybean. These include *Bradyrhizobium*, *Rhizobium*, *Nordella*, *Nitrobacter*, *Novosphingobium*, *Phenylobacterium*, *Streptomyces_S1*, *Allostreptomyces*, and *Chitinophaga_S2*.

Notably, distinct responses to host plant species were observed at the genus or subgenus level. Among the three genera within the *Sphingomonadaceae* family, *Novosphingobium* (log_2_ fold change [log_2_FC] = 5.98; false-discovery rate [FDR]-adjusted *P* value = 1.44e−105) was highly specific to soybean, whereas *Sphingobium_S1* (log_2_FC = −6.88; FDR = 1.67e−93), *Sphingobium_S2* (log_2_FC = −6.12; FDR = 1.02e−83), and *Sphingomonas_S1* (log_2_FC = −5.03; FDR = 6.97e−32) were specific to maize. *Sphingomonas_S2* (log_2_FC = −0.77; FDR = 0.0107) showed no substantial host preference.

Within the genus *Burkholderia*, the subgenus group *Burkholderia_S1* (log_2_FC = −5.63; FDR = 2.50e−43) was highly specific to maize, whereas *Burkholderia_S2* (log_2_FC = 0.01; FDR = 0.9786) appears to have no preference (compare also with Fig. 3B). Similarly, within the *Streptomyces* genus, *Streptomyces_S3* (log_2_FC = 4.81; FDR = 7.82e−63) was highly specific to soybean, whereas *Streptomyces_S2* (log_2_FC = −2.00; FDR = 1.94e−07) showed a preference for maize, and *Streptomyces_S1* (log_2_FC = −0.15; FDR = 0.5928) was found in roughly equal proportions in soybean and maize.

In contrast, no taxonomic groups responded to plant species above the 5% threshold in bulk soil, with the exception of *Rudaea* (see Fig. S8 for complete data). This indicates a minor impact of crop plants on bulk soil microbiomes in the field.

### Nitrogen treatment affects soil and rhizosphere microbiomes directly and indirectly via host plant effects.

**FIG 5:**
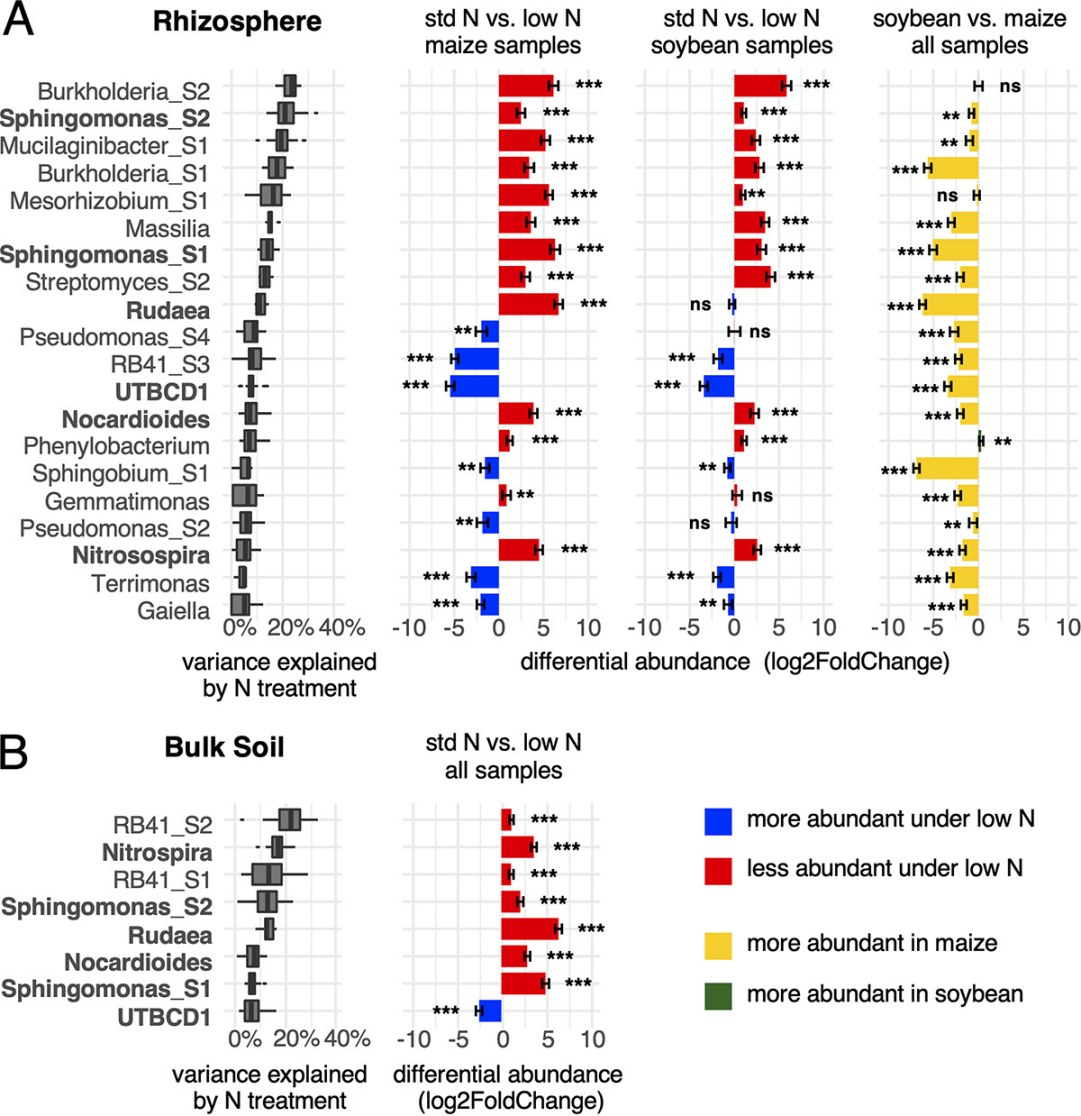
Microbial groups respond to N treatment in rhizospheres of either plant species and in bulk soil. Taxonomic groups at the genus and subgenus levels were ranked by the fraction of total variance explained by N treatment in the rhizosphere (A) and in bulk soil (B). Groups with a median variance score >5% are shown. Differential abundance of groups, log_2_ (abundance under std N/abundance under low N), was calculated pairwise for 48 maize and 48 soybean rhizosphere samples and for 96 bulk soil samples using DESeq2. For comparison, differential abundance in soybean versus maize in rhizosphere (green/yellow bars) is shown. Bars show standard errors, and asterisks indicate significantly different abundance between std N and low N at FDR-adjusted *P* values of <0.01 (***) and <0.05 (**). Taxa that showed response to N treatment in both rhizosphere and bulk soil are shown in bold.

Figure 5 shows microbial taxa that respond to N treatment at a threshold of >5% variance explained. We hypothesized that the N treatment would affect rhizosphere microbiomes of maize and soybean differently; hence, differential abundances of microbial taxa were analyzed separately for 48 low-N versus 48 standard (std)-N maize rhizosphere samples and for 48 low-N versus 48 std-N soybean rhizosphere samples (Fig. 5A). For comparison, differential abundance of microbes between maize and soybean is shown as before (Fig. 5A, rightmost graph). For bulk soil, comparisons of 96 low-N versus 96 std-N samples were made with samples from both maize and soybean fields (Fig. 5B). The complete data are shown in Fig. S9. More taxa were responsive in rhizosphere samples (*n* = 20) than in bulk soil (*n* = 8) at a threshold of >5% variance explained by N treatment. Notably, several taxa responded to N treatment both in bulk soil and in rhizospheres: *Nitrospira*, *Sphingomonas_S1*, *Sphingomonas_S2*, *Rudaea*, *Nocardioides*, and *UTBCD1* (marked bold in Fig. 5). Among these taxa, *UTBCD1* increased under low N, whereas the other groups increased under std N in both bulk soil and rhizospheres. Two subgroups of genus *RB41*, *RB41_S1* and *RB41_S2*, were responsive to N treatment exclusively in bulk soil, whereas *RB41_S3* was responsive in both rhizospheres. *RB41_S1* and *RB41_S2* increased under std N, whereas *RB41_S3* was highly increased under low N.

Groups that responded to N treatment in both rhizospheres include *Burkholderia_S1*, *Burkholderia_S2*, *Mucilaginibacter_S1*, *Mesorhizobium_S1*, *Massilia*, *Streptomyces_S2*, *Pseudomonas_S2*, *Pseudomonas_S4*, *RB41_S3*, *Phenylobacterium*, *Sphingobium_S1*, *Gemmatimonas*, *Terrimonas*, and *Gaiella*.

These data suggest that N fertilization has a direct effect on 6 microbial taxa that respond in both rhizosphere and bulk soil environments, as well as an indirect effect on taxa that only respond in rhizospheres, which is likely induced by changes in the host plant rhizosphere.

### Maize rhizosphere microbiomes are affected by N deficiency.

The differential abundance of microbial groups in response to N treatment tends to be higher in maize than in soybean. This was noticed by calculating the means of absolute log_2_ fold change (low-N ASV counts versus std-N ASV counts) for maize and soybean in rhizospheres (maize mean log_2_FC of 1.945735 versus soybean mean log_2_FC of 0.9755595; Welch two-sample *t* test *P* value = 1.54e−05) as well as in bulk soil (maize mean log_2_FC of 1.51321 versus soybean mean log_2_FC of 0.8643147; *P* value = 0.001722). In accordance with this, the vast majority of taxa responding to N treatment are more abundant in maize than in soybean rhizospheres (Fig. 5A, rightmost graph). While responses to N treatments are generally more pronounced in maize rhizospheres than in soybean rhizospheres, the direction of the changes seems to be consistent between host plant species, with a few notable exceptions: *Rudaea* organisms are more abundant under std-N treatment than under low N in maize rhizospheres (and in bulk soil), whereas no response to N treatment was observed in soybean rhizospheres. Similarly, *Pseudomonas_S4* and *Pseudomonas_S2* increase in abundance under low N in maize rhizospheres but not in soybean rhizospheres. It is worth noting in this context that maize showed a severe N deficiency phenotype, especially late in the season. Together, these data show that variation in N levels likely has a direct effect on soil microbes as well as an indirect effect through the impact of N levels on plant health and root exudation, which is most apparent in maize.

## DISCUSSION

Through statistical modeling of individual ASVs and variance partitioning, we identified time of sampling, plant species, and N treatment as major factors that influence rhizosphere microbial communities, while no major effect of crop rotation was observed. Year-to-year variation due to different weather conditions is common in agricultural experiments, and soil microbial communities are known to be affected by changes in temperature or humidity (49, 50) (Fig. S2). Seasonal variation has an additional biological cause as host plant physiology—including root exudation—changes significantly as plants mature (27). Host plant effects may also partly explain why rhizosphere microbiomes are more sensitive overall to environmental variables than bulk soil microbiomes (Fig. 2). Apart from environmental factors, the host plant species is the most important factor shaping rhizosphere microbiomes. Genetic distance between plant species (28) and between genotypes of the same species (29) often correlates with differences in microbial communities. N fertilization had an effect on both rhizosphere and soil microbial communities, which has been observed before in maize (18). It may thus be possible to modify the composition of microbial communities in the field through plant breeding and the mode of fertilizer application, respectively.

For a detailed analysis of any changes induced by experimental conditions, we used a combination of variance partitioning data and DNA sequence information to identify functional taxonomic groups at the genus and subgenus levels. This strategy revealed several low-level taxonomic groups that respond to host plant genetics and N fertilization in maize/soybean agricultural systems. Although further validation is required to define functional groups of microbes across different experiments, the two subgroups of *Burkholderia* identified in this study were successfully reproduced in two independent data sets from different years and locations (Fig. S7), and they showed significantly different responses to host plant species (Fig. 3A and B). Most interestingly, traditional OTU picking would have failed to distinguish these two groups (Table S1). Thus, we demonstrated that multifactorial experimental designs may be exploited to identify functional taxonomic groups in microbiome studies using both 16S rRNA sequence information and experimental data.

In accordance with previous research (30), we observed strong responses to host plant species in both maize and soybean rhizospheres and no response in bulk soil sampled only a few centimeters away from root surfaces. An immediate effect of host plants on bulk soil microbiomes was not observed and not expected, as root exudate concentrations decline exponentially and reach virtually zero only 7 mm into the soil (31). The top 6 taxa responding to plant host species are specific to soybean (Fig. 4). Unsurprisingly, they include N-fixing bacteria such as *Bradyrhizobium*, *Rhizobium*, and closely related *Nordella*. These were previously identified as key components of soybean microbiomes (32). *Nitrobacter* is closely related to *Bradyrhizobium* and involved in nitrite oxidation (33). *Novosphingobium*, *Phenylobacterium*, *Streptomyces*, and *Allostreptomyces* have no known role in the N cycle. Notably, *Novosphingobium* is highly specific to soybean, and *Sphingobium* and *Sphingomonas* are specific to maize, while all three genera are members of the *Sphingomonadaceae* family. These results once again underline the importance of adequate taxonomic resolution and suggest that maize and soybean rhizospheres are colonized by highly specialized groups of microbes, which may have evolved symbiotic relationships with their respective hosts.

The vast majority of taxa responding to N fertilizer are more abundant in maize rhizospheres than in soybean rhizospheres, whereas soybean-specific taxa generally do not respond to N treatments (Fig. 5A, rightmost graph, and Fig. S8). We attribute this to the established fact that maize shows a severely stressed phenotype under N deficiency, especially late in the season, which induces dramatic changes to root architecture, including root hair length and density (34). N-limited conditions have also been shown to alter plant root exudate profiles (35, 36). In contrast, soybean plants are hardly affected if fields are not fertilized. Thus, we identified two factors that shape microbial communities in agricultural systems: direct application of N fertilizer to the soil, which should affect both rhizosphere and bulk soil microbes, and changes due to altered root architecture and exudation patterns in response to N deficiency, which should mainly affect rhizosphere microbiomes. In accordance with this, we found more taxa affected by N treatment in rhizospheres than in bulk soil. Microbial taxa directly affected by N treatment are likely the ones that show a response to N treatment in both rhizosphere and bulk soil samples (marked in bold in Fig. 5). All other taxa are likely affected indirectly, and reduced abundance under N deficiency may be due to reduced vigor of the host plant rather than due to a simple lack of inorganic N to consume. These findings also support the idea that plant rhizospheres are colonized by highly specialized groups of microbes that are intimately tied to the host.

Taxa that increase in abundance under standard N fertilization are often capable of directly metabolizing ammonia or nitrate. *Gemmatimonas*, *Nitrospira*, *Mesorhizobium*, *Burkholderia*, *Rudaea*, *RB41*, and *Sphingomonas* were shown to be key players in nitrification (37) and N assimilation (38). On the other hand, taxa that increase in abundance under low-N conditions in plant rhizospheres may be able to take advantage of reduced plant vigor under N deficiency. Conversely, some microbes may also be actively recruited by plants if they confer a growth or disease resistance benefit under low-N stress conditions. The *Pseudomonas* genus contains both opportunistic pathogens and strains with plant growth-promoting activity (38), and some groups have previously been observed in maize rhizospheres under low-N conditions. *Terrimonas*, *Gaiella*, and *Gemmatimonas* have been observed in maize rhizospheres before (39), although their function is unknown. *UTBCD1* (*Chitinophagaceae*) and *RB41_S3* (*Pyromonadaceae*), both uncultured bacteria, increased the most under low-N conditions. Overall, surprisingly little is known about these taxa that respond positively to N deficiency in rhizospheres, and it remains to be determined whether they are simple opportunists, whether they cause disease, or whether they actively respond to changes in root exudate profiles under low-N conditions and, if so, whether they have plant growth-promoting capabilities that could be exploited to improve agricultural production.

### Conclusions.

In this study, we observed that rhizosphere and bulk soil microbiomes are primarily shaped by seasonal effects due to environmental changes, host plant species, and N treatment, whereas crop rotation of maize and soybean seems to be of minor importance. This suggests that maize and soybean rhizosphere microbiomes can potentially be manipulated through targeted plant breeding and farm management. We defined a set of 82 functional taxonomic groups at the genus and subgenus levels based on both 16S rRNA sequence information and responses to treatment variables, several of which are highly adapted to either maize (e.g., *Sphingobium*) or soybean (e.g., *Novosphingobium*) and may thus be relevant to the health and performance of their respective host. Lastly, we showed that N fertilization or the lack thereof has a direct effect on the abundance of several groups of microbes in bulk soil and rhizospheres as well as a possible indirect effect via reduced host plant vigor that is most apparent in maize. The findings presented in this work enhance our understanding of the key factors that influence rhizobiome compositions in two major crop plants under conventional and N-limited farming practices. Further research in this direction may open avenues to sustainably improve crop performance in the agricultural industry.

## MATERIALS AND METHODS

### Experimental design and sample collection.

**FIG 6:**
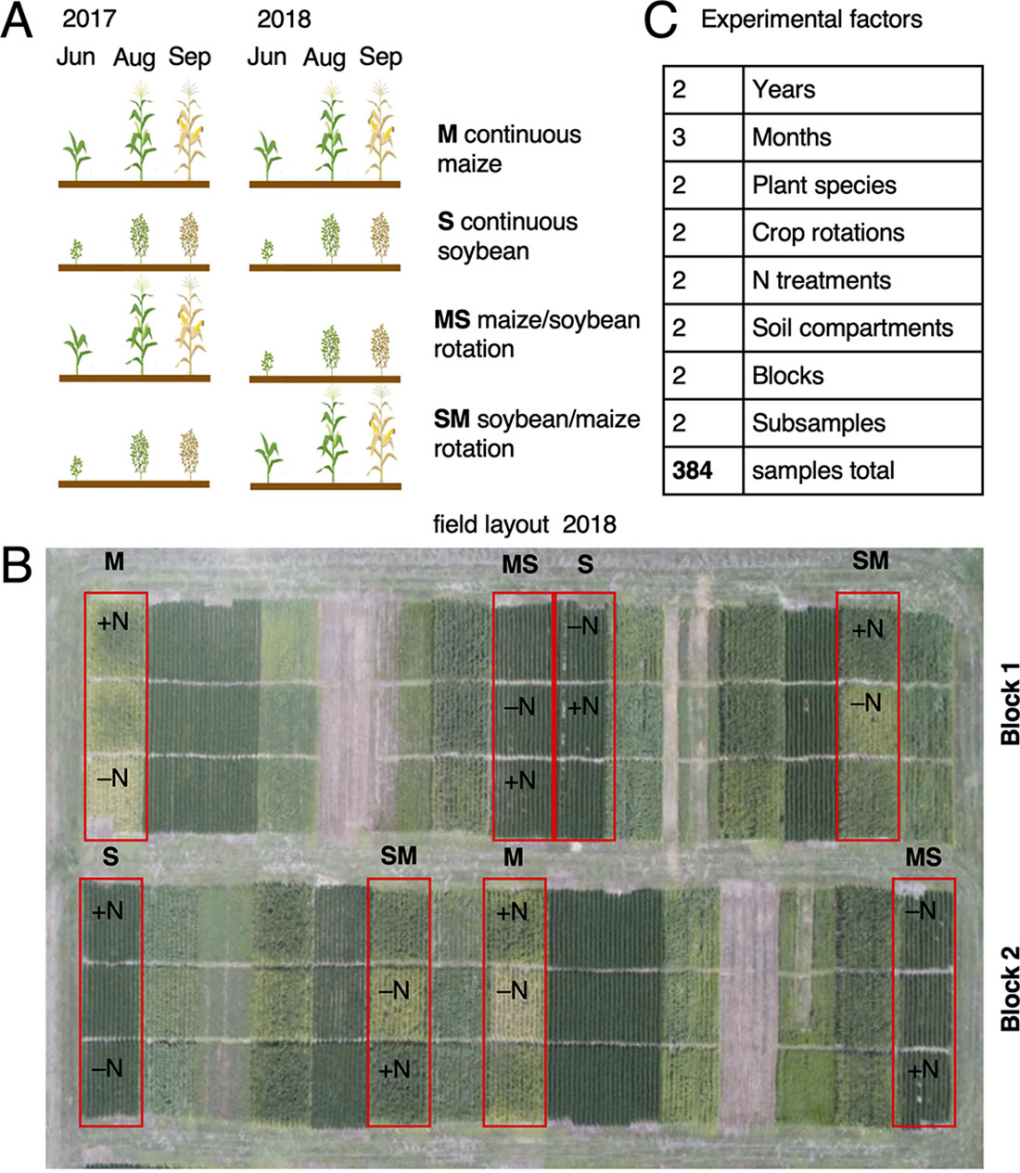
Experimental design. (A) Maize (M) and soybean (S) continuous crops as well as crop rotations (MS and SM) were tracked in June, August, and September in two consecutive years. (B) Field layout in the second year showing experimental blocks, maize or soybean plots (outlined in red), and subplots with either low (–N) or standard N treatment (+N) separated by alleys. (C) Overview of treatment factors analyzed in this study.

Maize and soybean plots in a historic crop rotation study at the Eastern Nebraska Research Extension Center near Mead, NE (41.167380, −96.418667), were arranged in a randomized complete block design. Seeds were not sterilized prior to planting. Plant growth conditions and field management are the same as described by Sindelar et al. (40). The long-term weather information can be accessed at the USDA-ARS Agricultural Collaborative Research Outcomes System (AgCROS) website (https://agcros-usdaars.opendata.arcgis.com/). For this study, plants were sampled from two replicate blocks in each of two subsequent years (2017 and 2018). Each replication included four plots planted with continuous maize (M), continuous soybean (S), maize rotated with soybean (MS), and soybean rotated with maize (SM). Each plot contained a subplot with standard N treatment (180 kg/ha annually for maize, 68 kg/ha for soybean) and a subplot with low-N conditions (no added N). From each subplot, two rhizosphere and two bulk soil samples were collected in June, August, and September (7, 14, and 20 weeks after planting). In total, 384 samples were collected (Fig. 6). This experimental design made it possible to distinguish 5 experimental factors: year of sampling (year 1 or year 2), month of sampling (early, middle, and late season), plant species (maize or soybean), crop rotation (continuous versus rotated), and N treatment (standard N fertilization or low-N conditions). All analyses were conducted separately for rhizosphere soil and bulk soil.

### 16S rRNA sequencing and microbial community analysis.

Genomic DNA was extracted from 192 rhizosphere and 192 bulk soil samples using the DNeasy PowerSoil kit (Qiagen, Hilden, Germany). Paired-end sequencing of a 300-bp sequence spanning the V4 region of the ribosomal 16S rRNA was generated using the Illumina MiSeq platform (Illumina Inc., San Diego, CA) with V4_515F_Nextera and V4_806R_Nextera primers. ASVs were called using a dada2-based pipeline as described previously (41, 42). In brief, raw 16S reads were demultiplexed, checked for chimeras, and merged, and nonprokaryotic sequences as well as low-abundance ASVs were removed. A phylogenetic tree of ASVs was constructed using MAFFT (43) and FastTree (44).

### Grouping of ASVs into functional taxonomic groups.

ASVs were initially grouped at the genus level, the lowest taxonomic level where groups of operational taxonomic units (OTUs) or ASVs can be reliably annotated using short reads of 16S rRNA gene sequence alone based on the SILVA reference database (45). To achieve better taxonomic resolution, we further identified functional taxonomic groups by grouping ASVs that respond consistently to experimental treatments (see Results). To validate this approach, we identified the same functional groups in two independent external data sets of 181 and 250 maize rhizosphere samples, respectively.

As a control, ASVs were clustered into OTUs using open-reference OTU picking in qiime (46) for all genera in which functional subgroups were identified. The number of OTUs generated was compared to the number of groups identified through manual identification of functional subgroups to test whether OTU picking would identify the same groups (Table S1).

### Statistical analysis.

For a total of 373 samples, constrained principal-coordinate analysis based on Bray-Curtis dissimilarity and permutational multivariate analysis of variance (PERMANOVA) was performed using R package vegan (47) with the model dissimilarity ∼ Year + Month + Host species + Crop rotation + Nitrogen + Block + Host species:Nitrogen. Shannon diversity metrics were calculated using R package phyloseq (48). Variance partitioning was performed on the ASV table with log-transformed relative abundances to estimate the contribution of each treatment factor to changes in microbiome composition in rhizosphere and bulk soil. For each of 2,225 ASVs present in rhizospheres and a subset of 2,014 ASVs present in bulk soil, the fraction of total variance explained by each treatment factor was calculated using R package lme4 (25) with the model log(ASV relative abundance) ∼ Year + Month + Host species + Crop rotation + Nitrogen + Block + Subsample, where all factors were fit as random effects.

Differential abundance of taxonomic groups in response to treatments was calculated with R package DESeq2 (26). Starting from the ASV table with raw sequence counts, ASVs were agglomerated into 82 taxonomic groups identified above, and a +1 pseudocount was added to all table values. Unless stated otherwise, 96 samples were used for comparisons, e.g., 96 soybean rhizosphere samples versus 96 maize rhizosphere samples.

For a detailed description of experimental procedures, view the methods in the supplemental material.

### Data availability.

The Sequence Read Archive (SRA) accession number for the sequencing data reported in this paper is PRJNA669400. The two external data sets we used for validation can be accessed under numbers PRJNA685208 and PRJNA685228. Scripts used to analyze the data are available on GitHub (https://github.com/mandmeier/USDA_CornSoy).
