# Supplementary material for: Rhizosphere Microbiomes in a Historical Maize-Soybean Rotation System Respond to Host Species and Nitrogen Fertilization at the Genus and Subgenus Levels
Source: Appl Environ Microbiol. 2021 May 26;87(12):e03132-20. doi: 10.1128/AEM.03132-20 (PMC8174755; doi:10.1128/AEM.03132-20)
Supplement: SUPPLEMENTAL FILE 1 — Supplemental material. Download aem.03132-20-s0001.pdf, PDF file, 1.1 MB [file aem.03132-20-s0001.pdf]

Supplementary Methods

Experimental field

Root and soil samples for this study were collected in 2017 and in 2018 from rain-fed experimental plots managed by USDA-ARS that had consistent long-term crop rotations and nitrogen fertilizer regimes in place for close to four decades. Plots are located at the University of Nebraska Agricultural research and Development Center near Mead, NE at the geographical location [41°10'00.7"N 96°25'07.2"W]. In brief, continuous maize (Zea mays L., Pioneer P1751AMT), continuous soybean (Glycine max L., Pioneer P31T11R), maize/soybean and other crop rotations were arranged in a randomized complete block design. Subplots were randomly assigned either low nitrogen (no extra fertilizer) medium nitrogen or high nitrogen treatments (180 kg/ha ammonium nitrate annually for maize, 68 kg/ha for soybean). Details about the rotation study are described by Peterson and Varvel (1,2).

Rhizobiome sampling

Root and soil samples were collected in June, early August, and September (7, 14, and 20 weeks after planting) in both 2017 and 2018. Samples were taken from maize and soybean plants in two different cropping systems (continuous and rotated) with two replicate plots per plant species and cropping system. Continuous crops were continuous maize (plots 115, 208), continuous soybean (plots 107, 215), crop rotations were maize in 2017/soybean in 2018 (plots 108 & 201) and soybean in 2017/maize in 2018 (plots 102, 210). On each of the 8 plots, samples were taken from both the low nitrogen and high nitrogen subplot. On each subplot, two subsamples were taken for a total of 4 replicates per plant species / cropping system / nitrogen treatment combination. Each replicate sample in turn consisted of pooled material from two adjacent plants that were randomly selected from the inner rows of each subplot.

Plant roots were dug up to a depth of 30 cm and rootstocks were manually shaken to remove loosely adherent soil. This soil was collected from two adjacent plants, homogenized and 15 ml was collected in a 50 ml tube. We called this fraction bulk soil. To gather a representative root sample, roots from two plants were cut into 5 cm pieces, homogenized and one 50 ml tube was filled with random root material. In soybean, root nodules were removed before cutting the roots to decrease the abundance of (well studied) nodule-forming rhizobium symbionts. Also, as soybean roots are relatively small early in the growing season up to 5 adjacent plants were pooled to gather enough root material for the June time points.

Sample processing

Root and bulk soil samples were immediately put on ice, transferred to the lab and processed within 24 h. 30 ml phosphate buffer (46 mM NaH2PO4, 60 mM NA2HPO4, 200 µl/L Silwet-77) was added to the root samples in 50 ml tubes and samples were vortexed horizontally for 3 min at 8000 rpm to shake tightly adherent soil off the roots. This fraction we call the rhizosphere. The rhizosphere soil suspension was filtered through a 100 µm nylon cell strainer (Celltreat Scientific Products, Pepperell, MA, USA) to remove residual plant material. The filtrate was centrifuged for 10 min at 4000 g. Supernatant was discarded and the soil pellet was stored at -20°C. For bulk soil samples, 30 ml phosphate buffer was added to 15 ml soil collected from the field and the sample was vortexed, filtered and processed in the same way as the rhizosphere samples.

In total, 2 crop species x 2 crop rotations x 2 N treatments x 2 years x 3 time points x 4 replicates x 2 fractions amounted to 384 samples.

DNA isolation and quantification

DNA was isolated using the DNeasy PowerSoil kit (Qiagen, Hilden, Germany) for both rhizosphere and bulk soil samples, starting with up to 250 mg material. Differing from the standard protocol, all samples were eluted in 50 µl TE (10 mM Tris-HCl, 1 mM disodium EDTA, pH 8.0) passed twice over the ion exchange column to increase DNA concentration. DNA was quantified fluorometrically using the QuantiFluor® dsDNA System (Promega, Madison, WI, USA). DNA extraction was repeated with fresh material if DNA concentration was less than 1 ng/µl.

16 S library preparation and sequencing

DNA samples were processed at the University of Minnesota Genomics Center (Minneapolis, MN, USA). In brief, the 16S rRNA V4 region was amplified using V4_515F_Nextera and V4_806R_Nextera primers and sequencing library preparation as described by Gohl (3). For the 2018 samples, oligonucleotide PCR blockers (PNA Bio INC, Thousand Oaks, CA, USA) targeting mitochondrial and chloroplast sequences were applied in the primary V4 amplification to reduce amplification of templates derived from eukaryotes. Up to 120 barcoded samples were pooled per sequencing run and sequenced on an Illumina MiSeq platform.

Raw read processing and construction of ASV table

16S sequencing reads were processed in R 3.5.2 using a workflow described by Callahan (4), which employs the package dada2 1.10.1 (5). Cluster computing resources at the UNL Holland Computing Center were used for computationally demanding steps. In brief, ~300 bp raw sequencing reads were trimmed using *filterAndTrim()* at 240 bp (forward reads) and 200 bp (reverse reads), respectively. Amplicon sequence variants (ASV) were inferred using *dada()* and forward and reverse reads were merged with *mergePairs()*. A sequence table was generated using *makeSequenceTable()* and chimaeras were removed using *removeBimeraDenovo()*.

Taxonomy was assigned to ASVs with *assignTaxonomy()* using the SILVA database version 132 as a reference. SILVA was our taxonomy of choice because it is a relatively large 16S sequence database compared to alternative databases, it is regularly maintained and updated and it is widely used in ecological research, making our results comparable to other 16S studies. (13)

Taxonomic training data formatted for DADA2 (silva_nr_v132_train_set.fa.gz) was obtained from https://zenodo.org/record/1172783#.XhN6UxdKh24, as referenced by https://benjjneb.github.io/dada2/training.html on GitHub. 16S reads and sample data were prepared in an R Phyloseq object for further processing.

ASV table filtering

Raw ASV reads were subjected to a series of filters to produce a final ASV table with biologically relevant 16S sequences: 1) Removed singleton 16S reads. 2) removed sequences that did not map to either Bacteria or Archaea. 3) Removed chloroplast sequences. 4) Removed mitochondrial sequences. 5) Prevalence filter: A range of prevalence filters was tested with x observations in n number of samples.

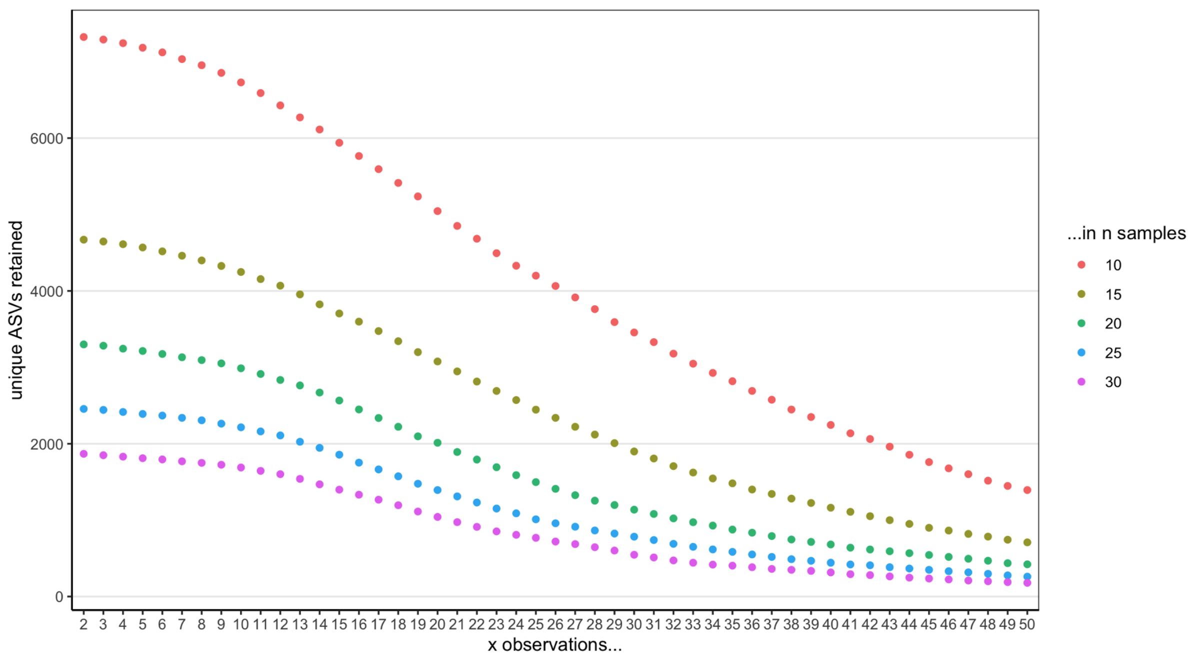

Prevalence filtering of ASV table. To find a workable prevalence filter, various thresholds “x observations in n samples” were tested. We decided to set the threshold to retain ASVs to 10 observations in at least 20 samples right before rapid loss of ASVs is observed (green curve).

To eliminate the vast majority of low-abundance sequences, ASVs were retained that had at least 10 observations in at least 20 samples. 6) Removed any reads that failed to be classified down to the genus level using SILVA taxonomy. This excludes reads where taxonomy is unclear or ambiguous and ensures efficient grouping of ASVs at the genome level later on. 7) Removed any ASVs where log transformed relative abundances were not normally distributed across 384 samples. The ASV table from step 6 was converted to relative abundances and values were transformed with the natural logarithm. Using the R “stats” package, normality was assessed for each ASV individually using the Shapiro-Wilks test shapiro.test(). Shapiro p-values were adjusted

with the Benjamini-Hochberg procedure to account for false discovery rate. ASVs were discarded if p_adj < 0.05, meaning a significant difference from a normal distribution. The resulting set of 2225 ASVs was used to generate a phylogenetic tree using mafft v. 7.404 (6) for multiple alignment and fasttree v. 2.1 (7) and the phylogenetic tree was attached to the phyloseq object. Lastly, 11 samples were removed from the data set that had fewer than 1000 total ASV counts. The final ASV table thus contained 2225 ASVs x 373 samples. This table was again converted to relative abundances and log transformed.

Grouping of ASVs into functional taxonomic groups

ASVs were initially grouped at the genus level, the lowest taxonomic level where groups of operational taxonomic units (OTUs) or amplicon sequence variants (ASVs) can be reliably annotated using short reads of 16S rDNA alone based on the SILVA reference database (8). Sub-genus functional groups were further identified through taxonomic clustering of each genus' ASVs and associated variance partitioning data. A phylogenetic tree of all ASVs was plotted together with the variance scores for each of 87 genera. This procedure allowed us to identify a total of 105 genera and sub-genus groups that show distinct and unambiguous responses to treatments. 82 groups that had at least five distinct ASVs were used for subsequent analyses. For each set of ASVs that mapped to a genus in which subgroups were identified, open-reference OTU picking was performed to cluster ASVs into OTUs. OTUs were generated from FASTA files using pick_open_reference_otus.py implemented in Qiime/1.9 (9) with default settings. The number of OTUs generated through this OTU picking procedure was compared to the number of groups identified through manual identification of functional genus subgroups (Table S1).

Statistical analysis

For a total of 373 samples, constrained principal coordinates analysis based on Bray-Curtis dissimilarity and Permutation ANOVA was performed using R package vegan (10) with the model dissimilarity ~Year + Month + Host species + Crop rotation + Nitrogen + Block + Host species:Nitrogen. Shannon diversity metrics were calculated using R package phyloseq (11). Variance partitioning was performed on the above ASV table using R package lme4 using the model log(ASV relative abundance) ~ Year + Month + Host Species + Crop Rotation + Nitrogen + Block + Subsample with all random factors.

Differential abundance of taxonomic groups in response to treatments was calculated with R package DESeq2 (12). Starting from the ASV table with raw sequence counts, ASVs were agglomerated into 82 taxonomic groups identified above, and a +1 pseudocount was added to all table values. Unless stated otherwise, n = 96 samples were used for comparisons, e.g. 96 soybean rhizosphere samples vs. 96 maize rhizosphere samples.

Data Availability

The Sequence Read Archive (SRA) accession number for the sequencing data reported in this paper is PRJNA669400. The two external datasets we used for validation can be accessed under PRJNA685208 and PRJNA685228, respectively. Scripts used to analyze the data are available on GitHub (https://github.com/mandmeier/USDA_CornSoy).

References

1. Peterson TA, Varvel GE. Crop Yield as Affected by Rotation and Nitrogen Rate. I. Soybean. Agronomy Journal. 1989;81(5):727.
2. Varvel GE. Crop Rotation and Nitrogen Effects on Normalized Grain Yields in a Long-Term Study. Agronomy Journal. 2000;92(5):938.
3. Gohl DM, Vangay P, Garbe J, MacLean A, Hauge A, Becker A, et al. Systematic improvement of amplicon marker gene methods for increased accuracy in microbiome studies. Nature Biotechnology. 2016 Sep;34(9):942–9.
4. Callahan BJ, Sankaran K, Fukuyama JA, McMurdie PJ, Holmes SP. Bioconductor Workflow for Microbiome Data Analysis: from raw reads to community analyses. F1000Research. 2016 Nov 2;5:1492.
5. Callahan BJ, McMurdie PJ, Rosen MJ, Han AW, Johnson AJA, Holmes SP. DADA2: High-resolution sample inference from Illumina amplicon data. Nature Methods. 2016 Jul;13(7):581–3.
6. Katoh K. MAFFT: a novel method for rapid multiple sequence alignment based on fast Fourier transform. Nucleic Acids Research. 2002 Jul 15;30(14):3059–66.
7. Price MN, Dehal PS, Arkin AP. FastTree: Computing Large Minimum Evolution Trees with Profiles instead of a Distance Matrix. Molecular Biology and Evolution. 2009 Jul 1;26(7):1641–50.
8. Yilmaz P, Parfrey LW, Yarza P, Gerken J, Pruesse E, Quast C, et al. The SILVA and “All-species Living Tree Project (LTP)” taxonomic frameworks. Nucl Acids Res. 2014 Jan;42(D1):D643–8.
9. Caporaso JG, Kuczynski J, Stombaugh J, Bittinger K, Bushman FD, Costello EK, et al. QIIME allows analysis of high-throughput community sequencing data. Nat Methods. 2010 May;7(5):335–6.
10. Oksanen J, Blanchet FG, Friendly M, Kindt R, Legendre P, McGlinn D, et al. vegan: Community Ecology Package. R package version 2.4-3. Vienna: R Foundation for Statistical Computing[Google Scholar]. 2016;
11. McMurdie PJ, Holmes S. phyloseq: An R Package for Reproducible Interactive Analysis and Graphics of Microbiome Census Data. Watson M, editor. PLoS ONE. 2013 Apr 22;8(4):e61217.
12. Love MI, Huber W, Anders S. Moderated estimation of fold change and dispersion for RNA-seq data with DESeq2. Genome Biology [Internet]. 2014 Dec [cited 2019 Apr 22];15(12). Available from: http://genomebiology.biomedcentral.com/articles/10.1186/s13059-014-0550-8
13. Balvočiūtė M, Huson DH. SILVA, RDP, Greengenes, NCBI and OTT — how do these taxonomies compare? BMC Genomics [Internet]. 2017 Mar [cited 2020 Jan 6];18(S2). Available from: http://bmcgenomics.biomedcentral.com/articles/10.1186/s12864-017-3501-4

Supplementary Figures

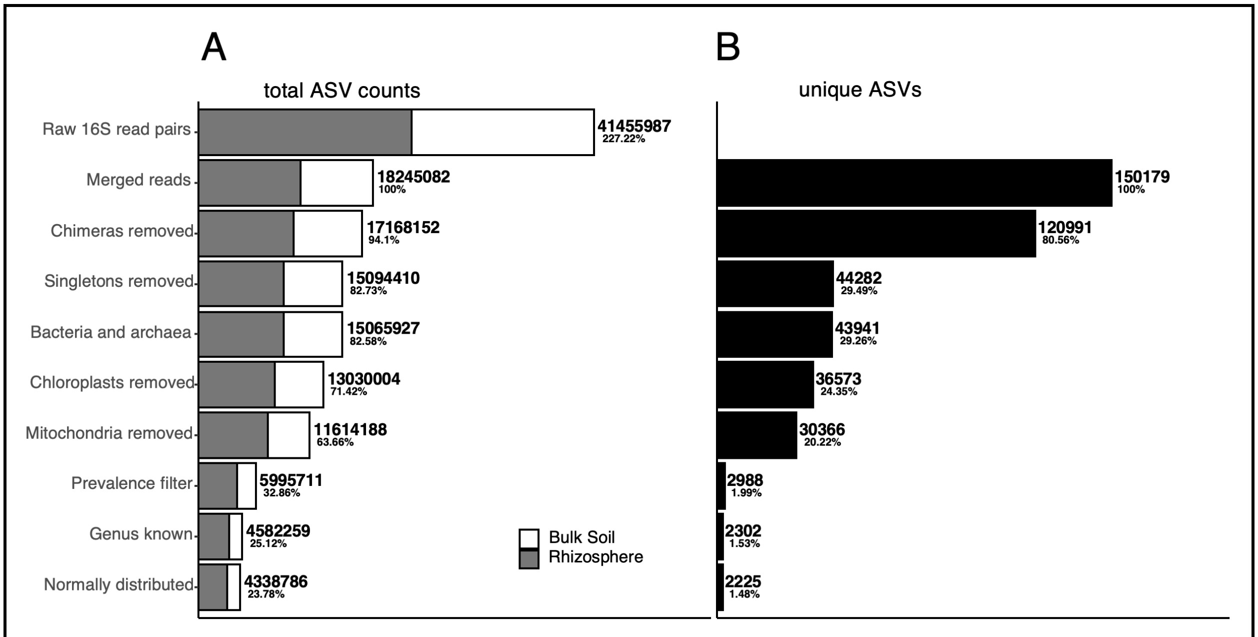

Fig. S1) ASV counts at each filtering step in total of 384 samples. A) total ASV counts B) total unique ASVs.

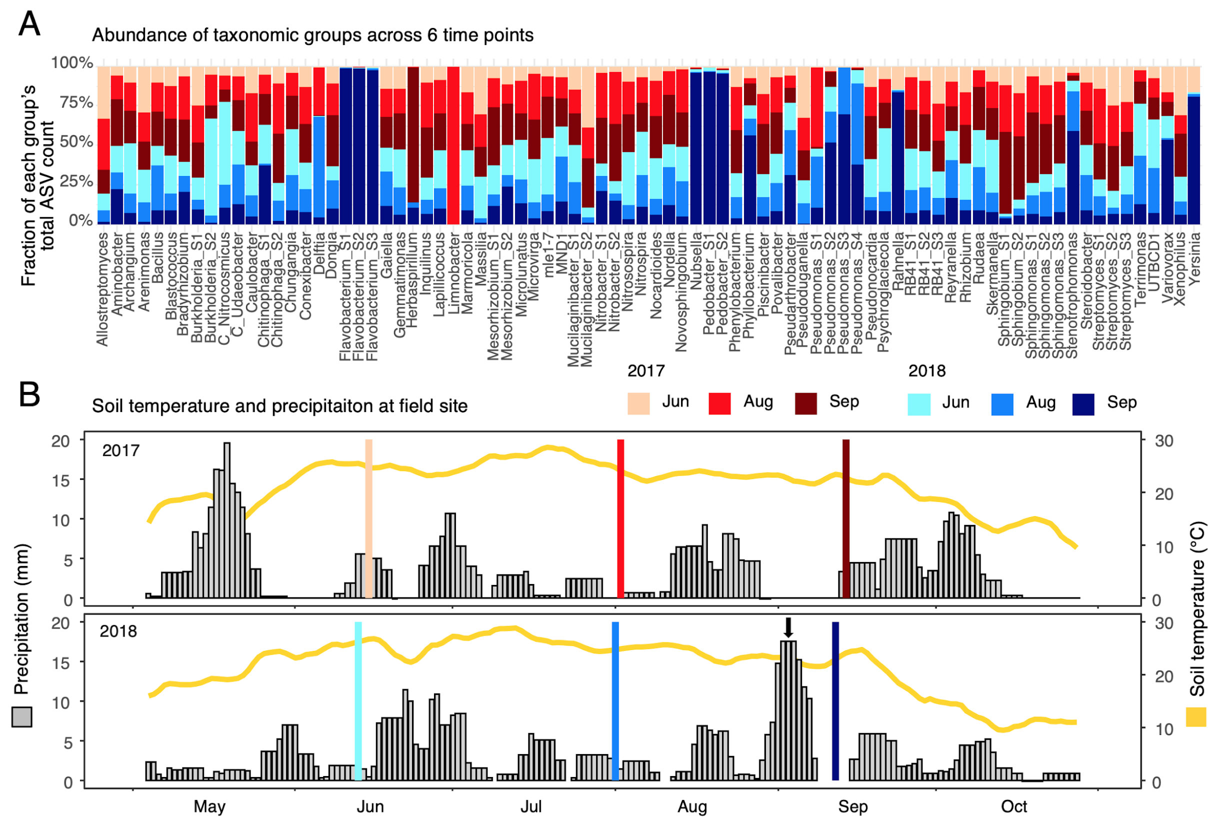

Fig. S2) **Microbial groups vary in abundance over time. Variation can be partly explained by weather effects.** A) Percentage of total ASV count per taxonomic group broken down by time point. B) Soil temperature (yellow line) and precipitation data (grey bars) at USDA field site near Memphis NE. Vertical bars indicate sampling days. These data reveal unusually high counts of several taxa (*Phyllobacterium*, *Flavobacterium*, *Nubsella*, *Pedobacter*, *Pseudomonas*, *Rahnella*, *Stenotrophomonas*, *Variovorax*, *Yersinia*) in September 2018. This is possibly an environmental effect due to a heavy rainfall event only days before sample collection (arrow).

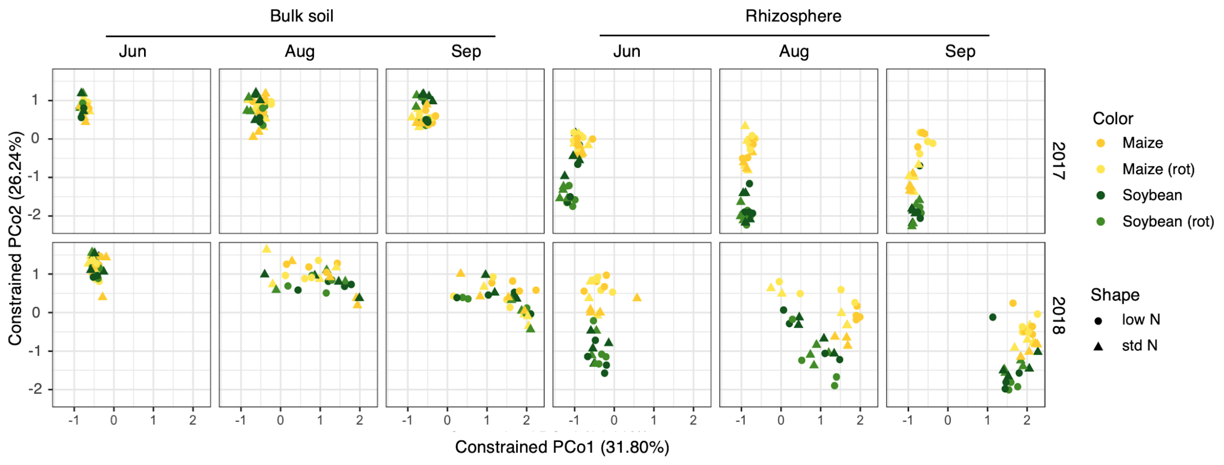

Fig. S3: **Principle Coordinates Analysis constrained by environmental and experimental factors**

Panels are separated by year, month, and sample fraction (bulk soil and rhizosphere). Shades of yellow denote maize samples and shades of green soybean samples. Lighter shades indicate rotated fields (rot). Shapes indicate low N (circles) and std N treatments (triangles), respectively.

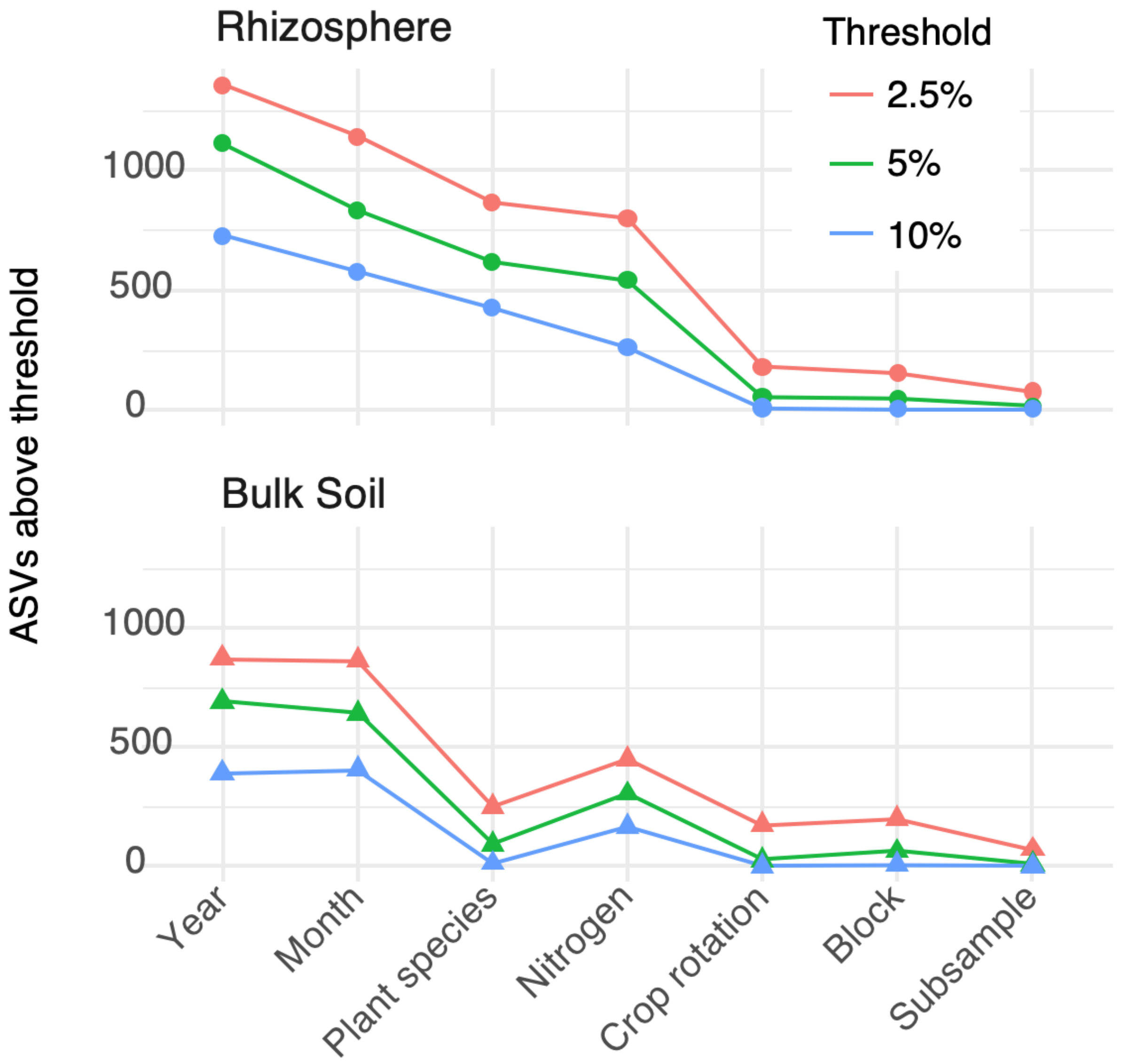

Fig. S4) Halving or doubling the chosen 5% arbitrary threshold reveals the same ranking of treatment factors in rhizosphere and bulk soil.

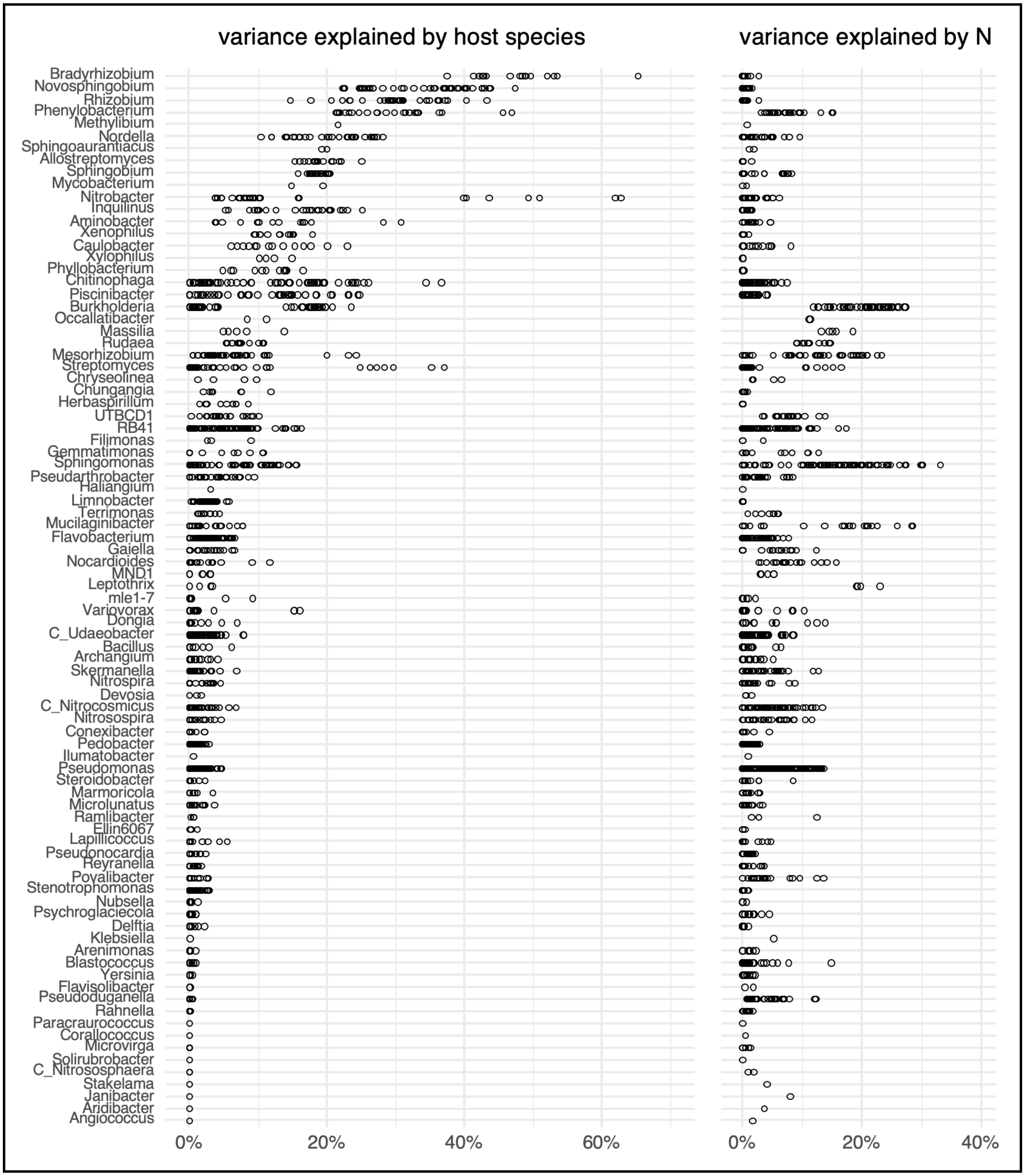
variance explained by host species
variance explained by N
Bradyrhizobium
Novosphingobium
Rhizobium
Phenylobacterium
Methylibium
Nordella
Sphingoaurantiacus
Allostreptomyces
Sphingobium
Mycobacterium
Nitrobacter
Inquilinus
Aminobacter
Xenophilus
Caulobacter
Xylophilus
Phyllobacterium
Chitinophaga
Piscinibacter
Burkholderia
Occallatibacter
Massilia
Rudaea
Mesorhizobium
Streptomyces
Chryseolinea
Chungangia
Herbaspirillum
UTBCD1
RB41
Filimonas
Gemmatimonas
Sphingomonas
Pseudarthrobacter
Haliangium
Limnobacter
Terrimonas
Mucilaginibacter
Flavobacterium
Gaiella
Nocardioides
MND1
Leptothrix
mle1-7
Variovorax
Dongia
C_Udaeobacter
Bacillus
Archangium
Skermanella
Nitrospira
Devosia
C_Nitrocosmicus
Nitrosospira
Conexibacter
Pedobacter
Ilumatobacter
Pseudomonas
Steroidobacter
Marmoricola
Microlunatus
Ramlibacter
Ellin6067
Lapillicoccus
Pseudonocardia
Reyranella
Povalibacter
Stenotrophomonas
Nubsella
Psychroglaciecola
Delftia
Klebsiella
Arenimonas
Blastococcus
Yersinia
Flavisolibacter
Pseudoduganella
Rahnella
Paracraurococcus
Corallococcus
Microvirga
Solirubrobacter
C_Nitrososphaera
Stakelama
Janibacter
Aridibacter
Angiococcus
0%
20%
40%
60%
0%
20%
40%

Fig. S5) Rhizosphere Variability explained by plant species (left) and N treatment (right) for each genus. Variation in this plot may indicate that subgroups in each genus respond differently to treatments.

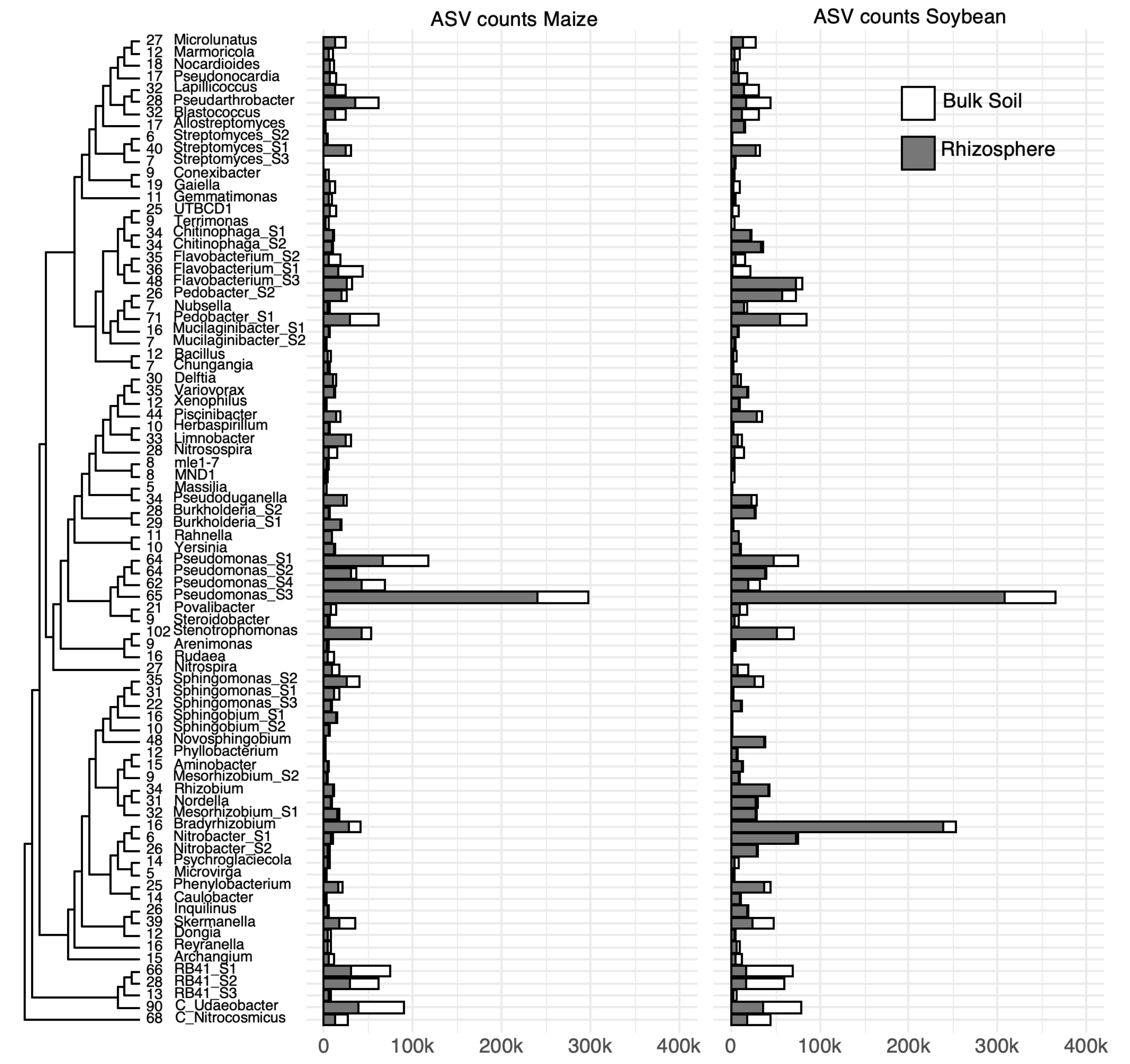

Fig. S6) Total ASV counts in maize and soybean bulk soil and rhizosphere for all 82 taxonomic groups. Numbers above cladogram tips indicate the number of unique ASVs observed in each taxonomic group. *Pseudomonas_S3* and in soybean *Bradyrhizobium* are the most abundant, mainly in the rhizosphere.

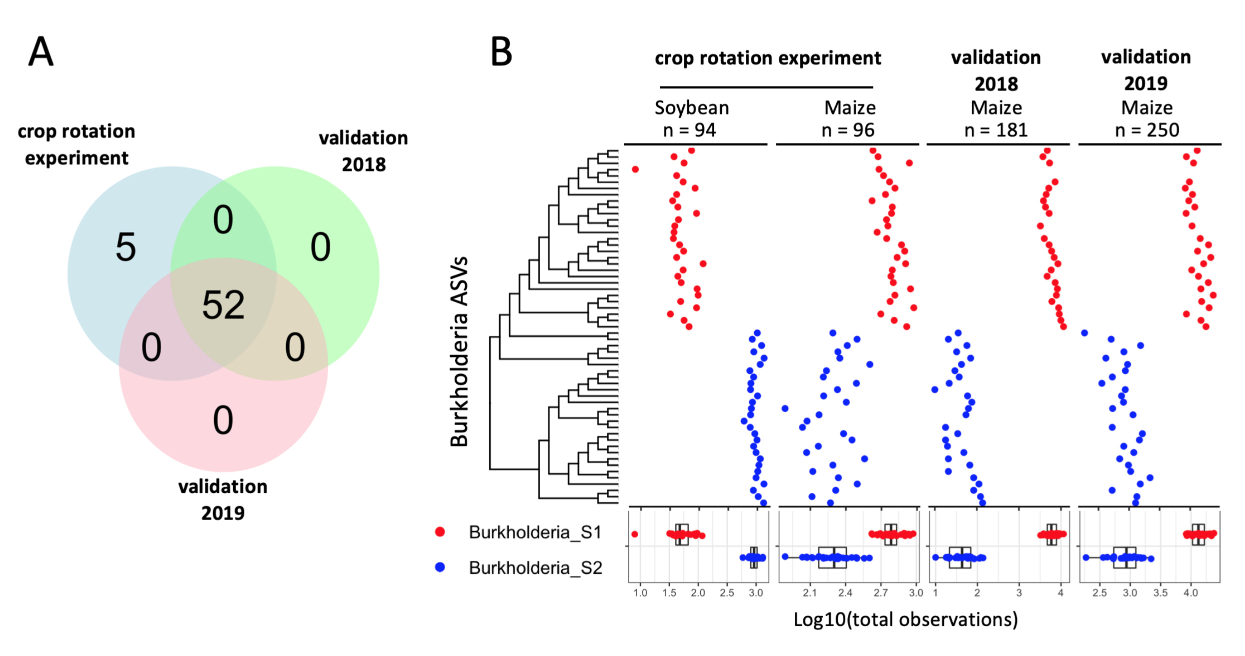

Fig. S7: **Sub-genus groups can be reproduced in independent experiments.**

A) Overlap of unique Burkholderia ASVs observed in this study and in two external experiments.

B) Total observations of ASVs in Burkholderia genus. Color indicates sub-genus groups.

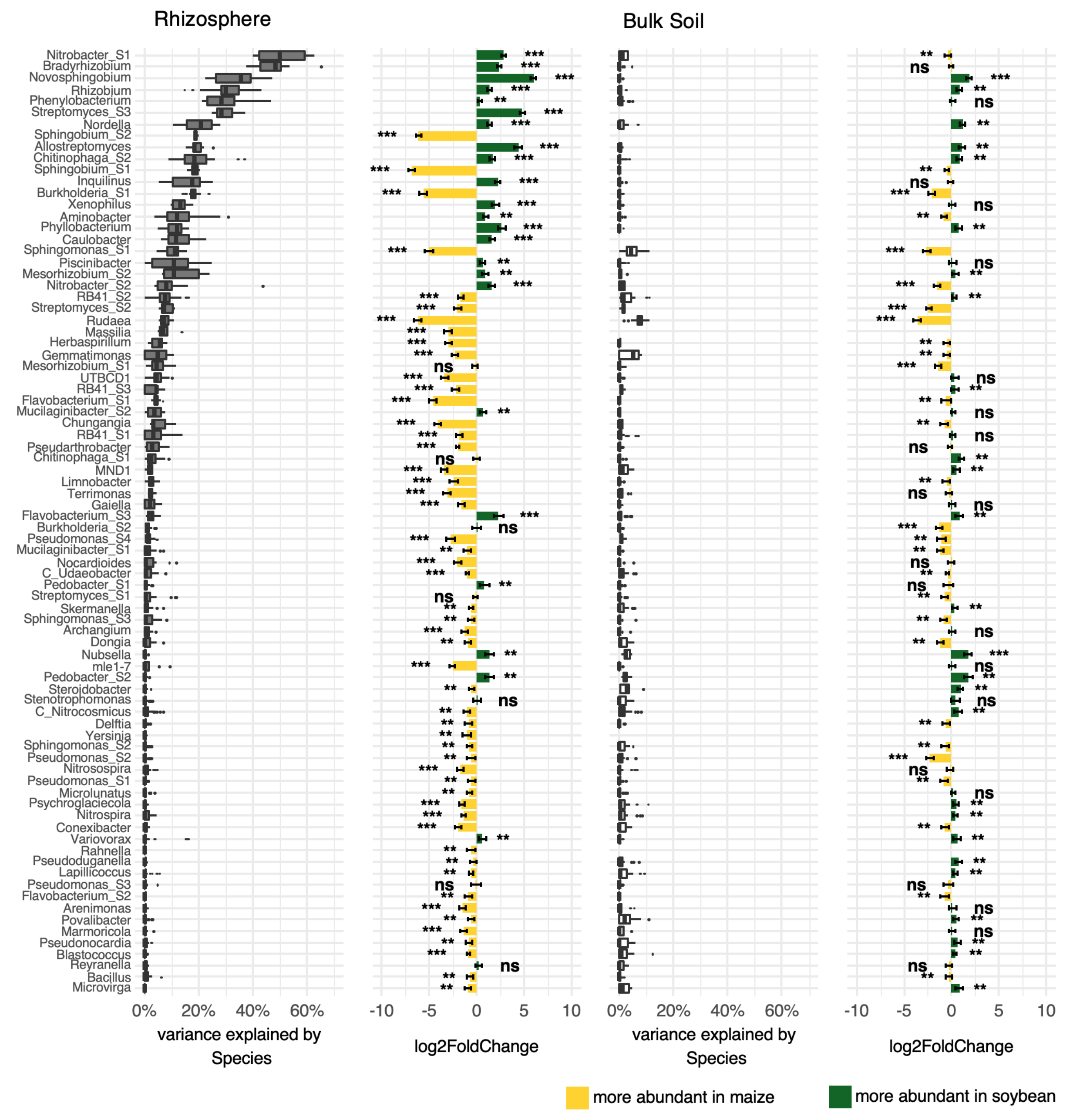

Fig. S8) Variance explained by host plant species, all 82 taxonomic groups. Notice that the host plant effect on bulk soil is minor.

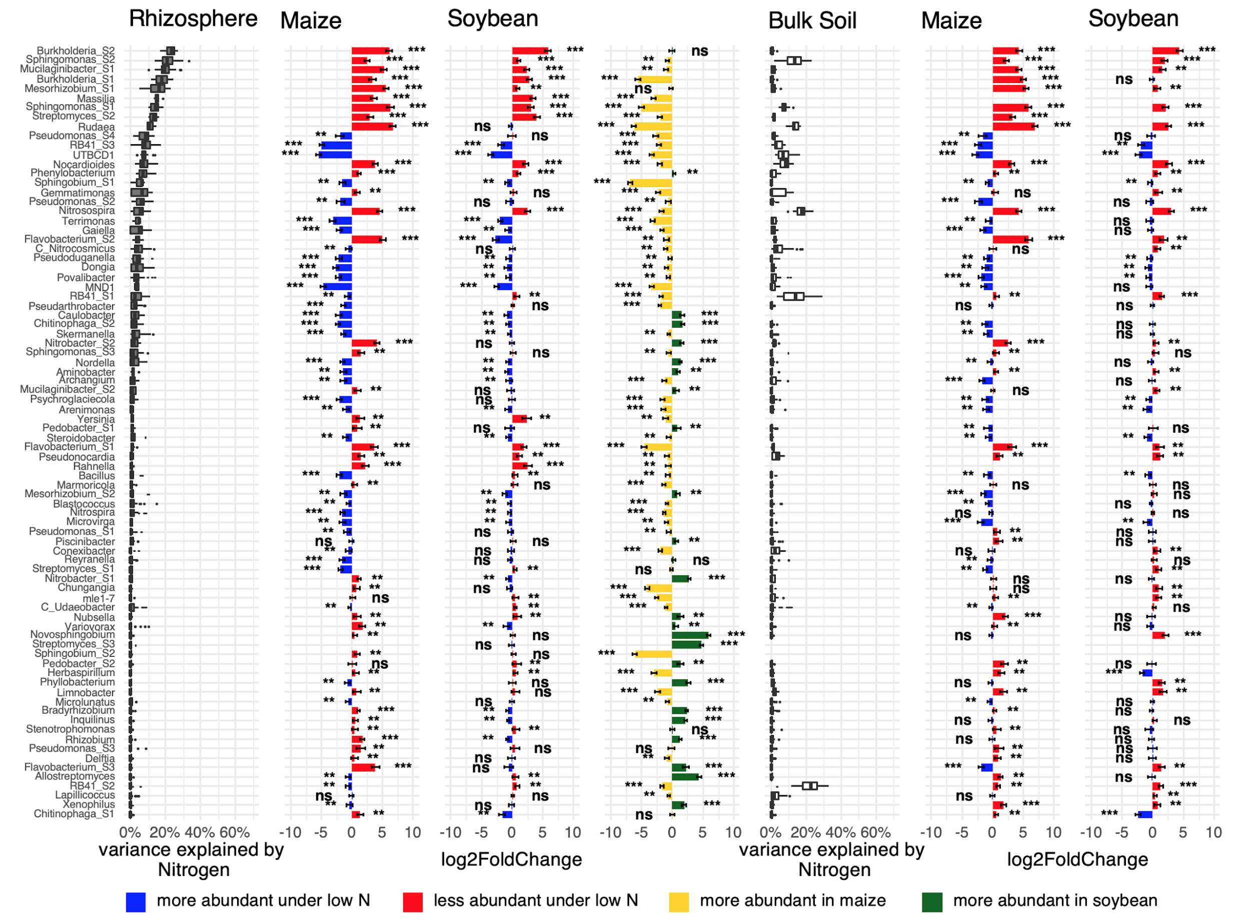

Fig. S9) Variance explained by N treatment, all 82 taxonomic groups. Notice that ranking of groups is different in bulk soil.

Genus	Subgroups identified by response to treatments	97% OTUs generated from ASVs
Burkholderia	2	1*
Chitinophaga	2	3
Flavobacterium	3	4
Mesorhizobium	2	3
Mucilaginibacter	2	2
Nitrobacter	2	1*
Pedobacter	2	2
Pseudomonas	4	3
RB41	3	7
Sphingobium	2	1*
Sphingomonas	3	4
Streptomyces	3	3

Table S1) **OTUs clustered from ASVs in each genus do not reproduce genus subgroups identified through variance partitioning data.**

OTU clustering at a fixed 97% identity threshold may fail to identify groups that respond to treatment factors as a unit. In some cases, 97% OTUs do not offer sufficient resolution to distinguish sub-genus groups at all (*).

Supplementary File1 :

Identification of sub-genus groups

- Phylogenetic tree of 64 genera with >5 ASVs plotted against variance explained by host species and nitrogen
- Sub-genus groups were identified for 12 genera

Genera with sub-genus groups

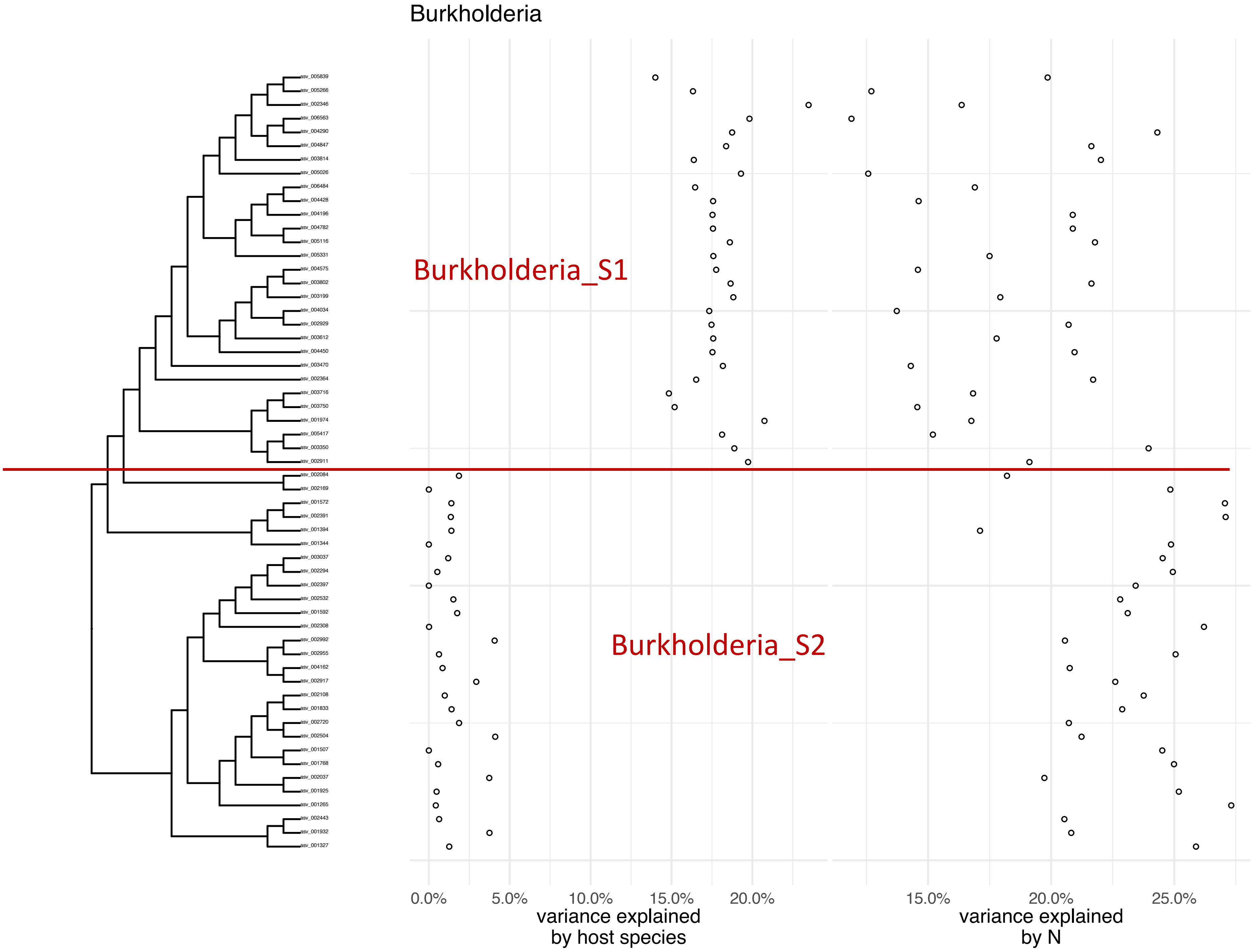
Burkholderia
asv_005839
asv_005266
asv_002346
asv_006563
asv_004290
asv_004847
asv_003814
asv_005026
asv_006484
asv_004428
asv_004196
asv_004782
asv_005116
asv_005331
asv_004575
asv_003802
asv_003199
asv_004034
asv_002929
asv_003612
asv_004450
asv_003470
asv_002364
asv_003716
asv_003750
asv_001974
asv_005417
asv_003350
asv_002911
asv_002084
asv_002169
asv_001572
asv_002391
asv_001394
asv_001344
asv_003037
asv_002294
asv_002397
asv_002532
asv_001592
asv_002308
asv_002992
asv_002955
asv_004162
asv_002917
asv_002108
asv_001833
asv_002720
asv_002504
asv_001507
asv_001768
asv_002037
asv_001925
asv_001265
asv_002443
asv_001932
asv_001327
Burkholderia_S1
Burkholderia_S2
0.0%
5.0%
10.0%
15.0%
20.0%
variance explained
by host species
15.0%
20.0%
25.0%
variance explained
by N

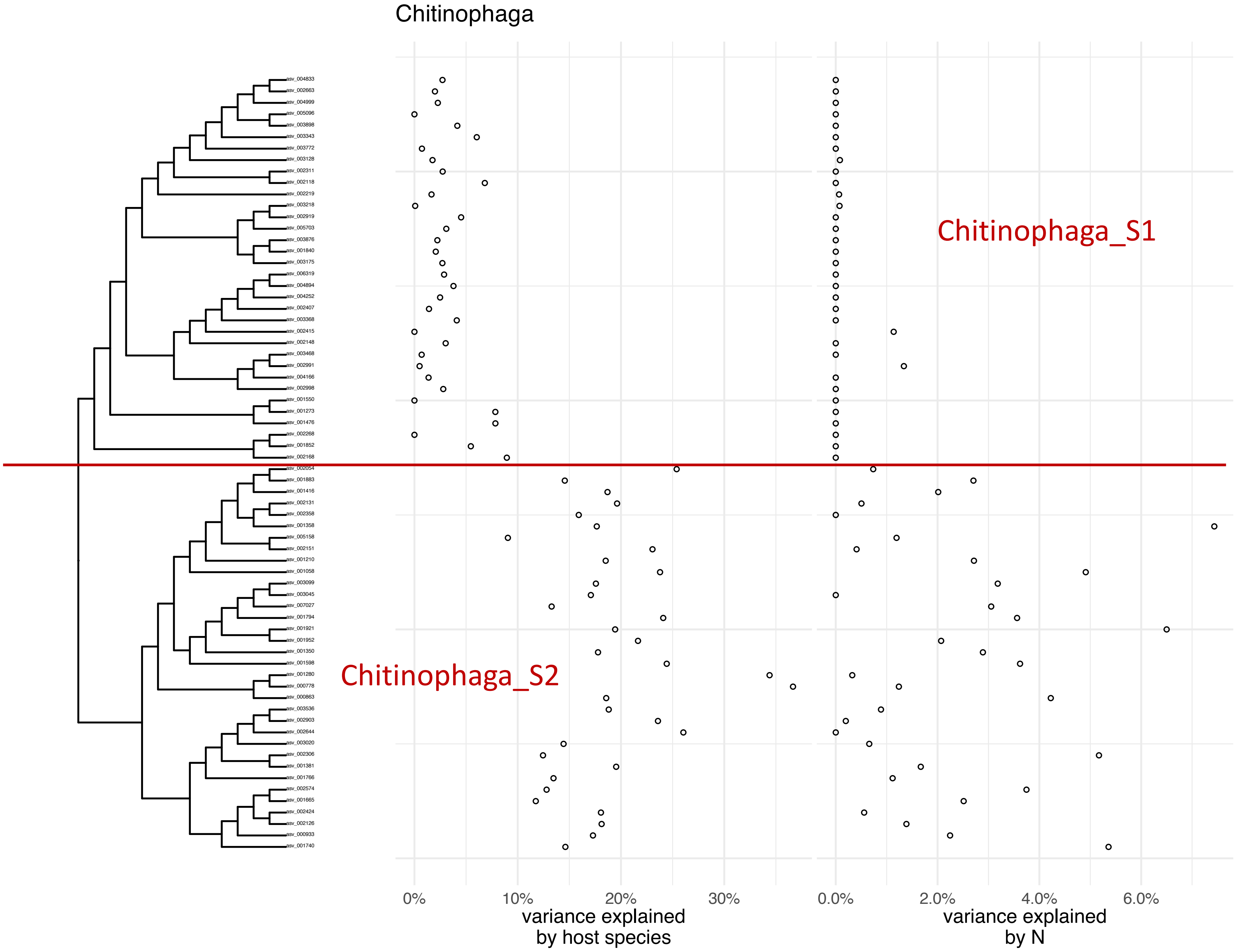
Chitinophaga
Chitinophaga_S1
Chitinophaga_S2
0%
10%
20%
30%
variance explained
by host species
0.0%
2.0%
4.0%
6.0%
variance explained
by N

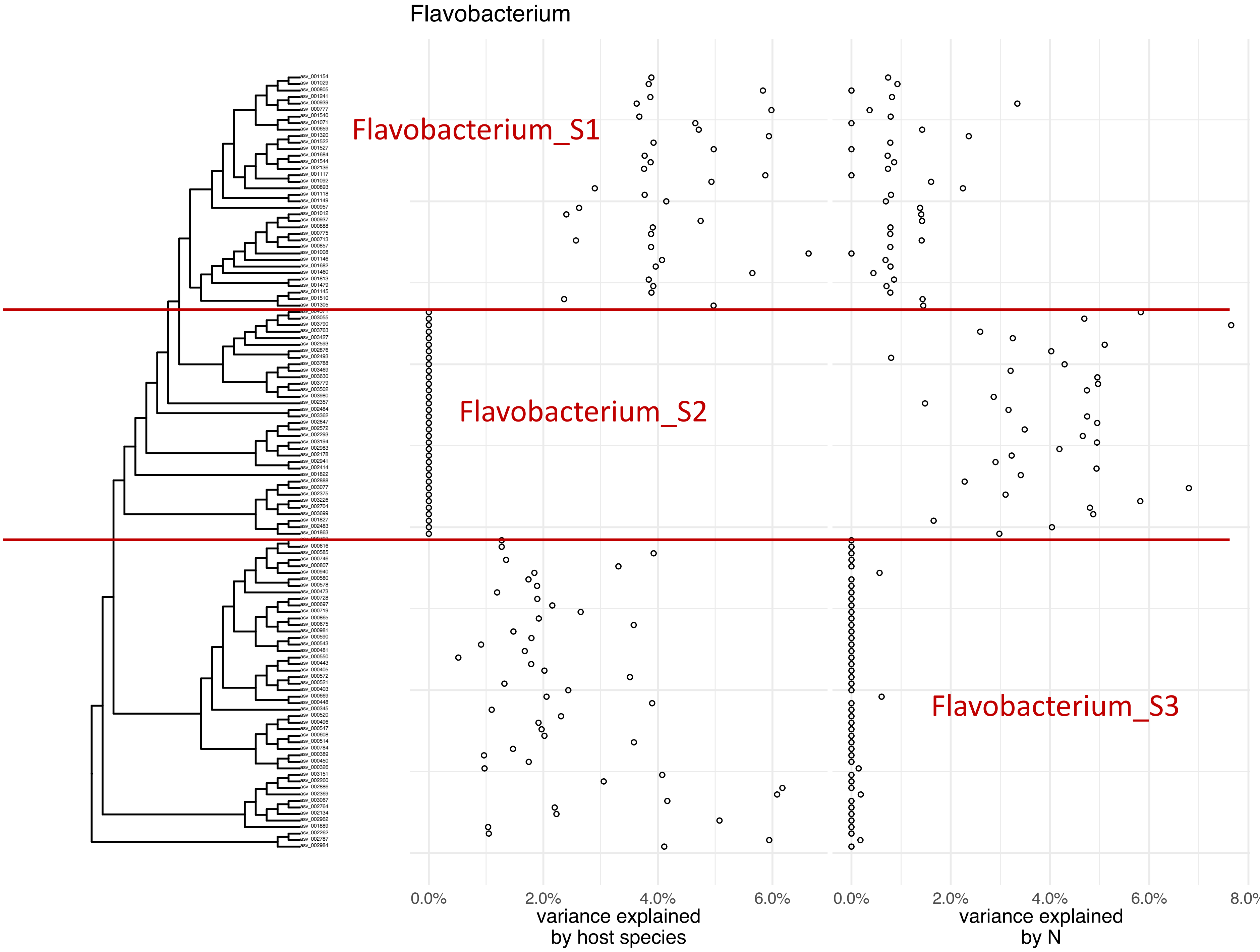
Flavobacterium
Flavobacterium_S1
Flavobacterium_S2
Flavobacterium_S3
0.0%
2.0%
4.0%
6.0%
variance explained by host species
0.0%
2.0%
4.0%
6.0%
8.0%
variance explained by N

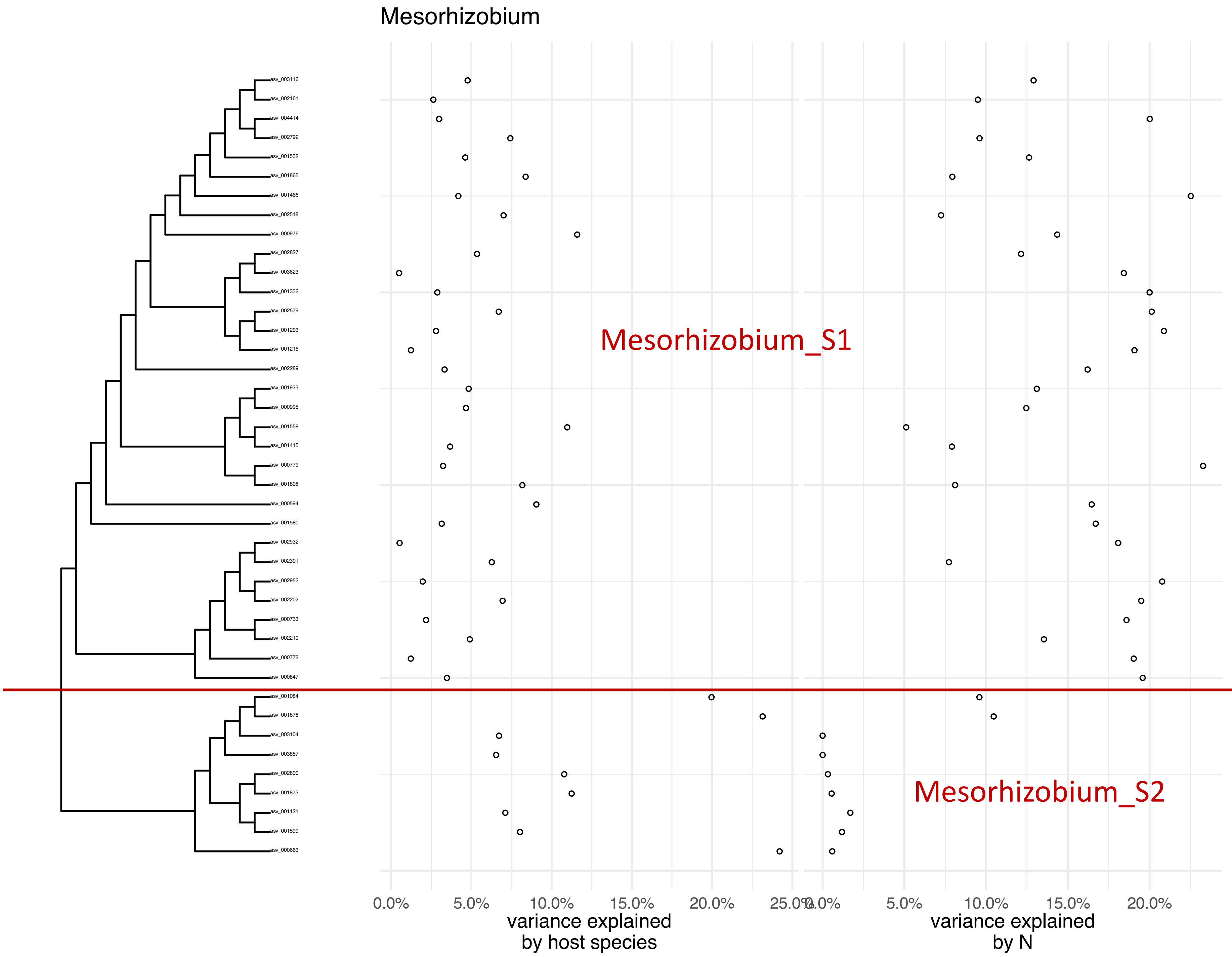

Mesorhizobium
asv_003116
asv_002161
asv_004414
asv_002792
asv_001532
asv_001865
asv_001466
asv_002518
asv_000976
asv_002827
asv_003623
asv_001332
asv_002579
asv_001203
asv_001215
asv_002289
asv_001933
asv_000995
asv_001558
asv_001415
asv_000779
asv_001808
asv_000594
asv_001580
asv_002932
asv_002301
asv_002952
asv_002202
asv_000733
asv_002210
asv_000772
asv_000847
asv_001084
asv_001878
asv_003104
asv_003857
asv_002800
asv_001873
asv_001121
asv_001599
asv_000663
Mesorhizobium_S1
Mesorhizobium_S2
0.0%
5.0%
10.0%
15.0%
20.0%
25.0%
variance explained
by host species
0.0%
5.0%
10.0%
15.0%
20.0%
variance explained
by N

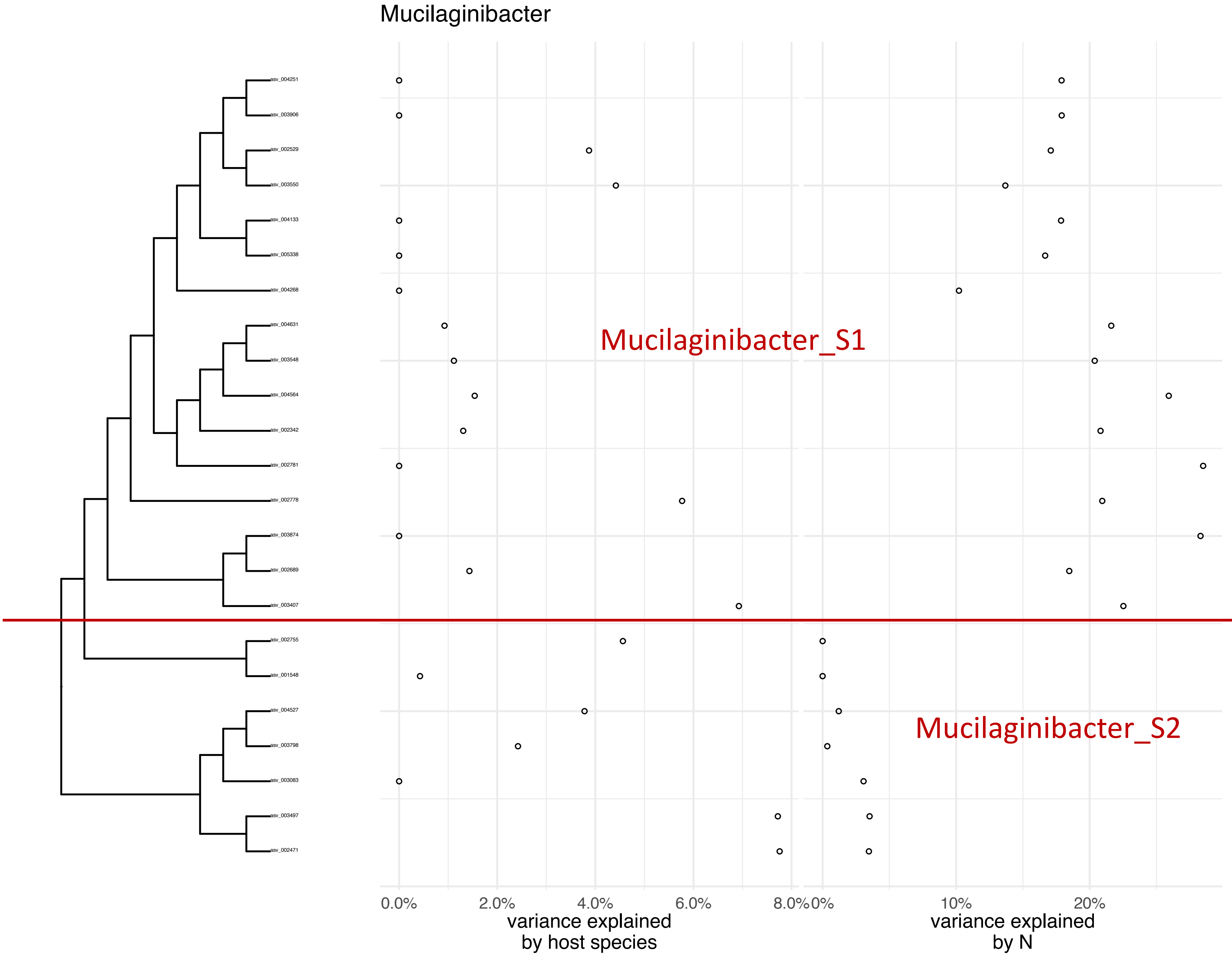
Mucilaginibacter
asv_004251
asv_003906
asv_002529
asv_003550
asv_004133
asv_005338
asv_004268
asv_004631
asv_003548
asv_004564
asv_002342
asv_002781
asv_002778
asv_003874
asv_002689
asv_003407
asv_002755
asv_001548
asv_004527
asv_003798
asv_003083
asv_003497
asv_002471
Mucilaginibacter_S1
Mucilaginibacter_S2
0.0%
2.0%
4.0%
6.0%
8.0%
variance explained
by host species
0%
10%
20%
variance explained
by N

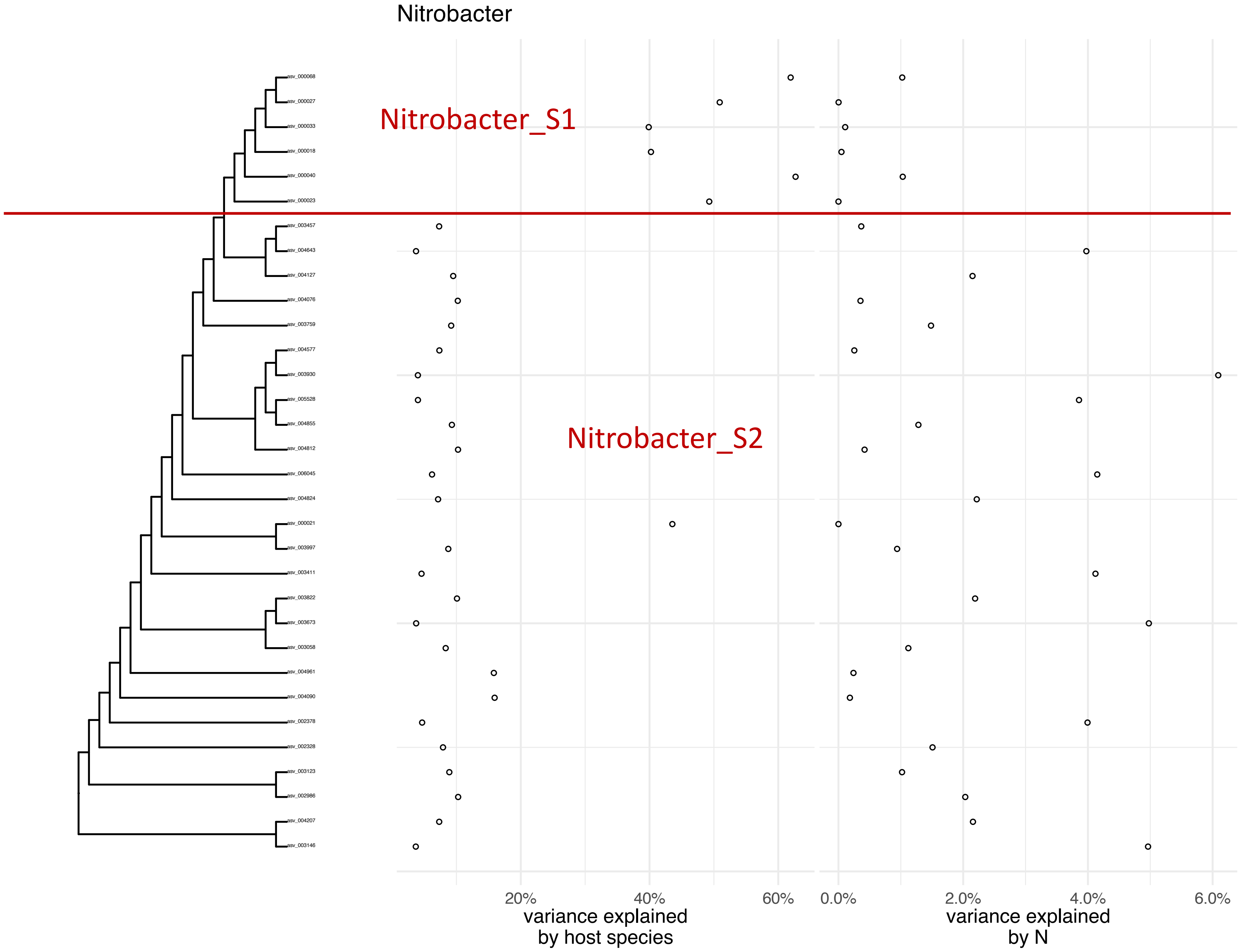
Nitrobacter
Nitrobacter_S1
Nitrobacter_S2
asv_000068
asv_000027
asv_000033
asv_000018
asv_000040
asv_000023
asv_003457
asv_004643
asv_004127
asv_004076
asv_003759
asv_004577
asv_003930
asv_005528
asv_004855
asv_004812
asv_006045
asv_004824
asv_000021
asv_003997
asv_003411
asv_003822
asv_003673
asv_003058
asv_004961
asv_004090
asv_002378
asv_002328
asv_003123
asv_002986
asv_004207
asv_003146
20%
40%
60%
variance explained by host species
0.0%
2.0%
4.0%
6.0%
variance explained by N

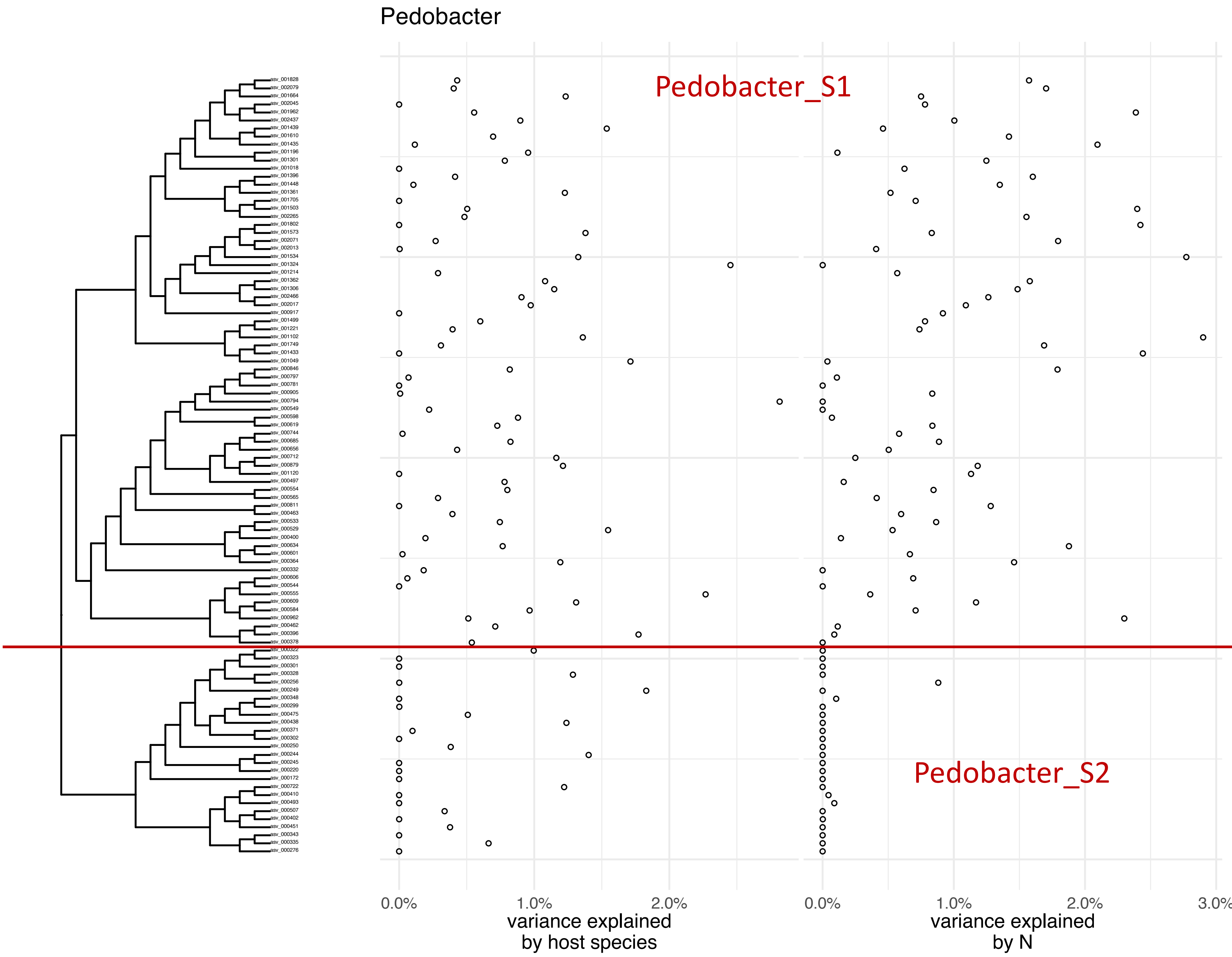
Pedobacter
Pedobacter_S1
Pedobacter_S2
0.0%
1.0%
2.0%
0.0%
1.0%
2.0%
3.0%
variance explained by host species
variance explained by N

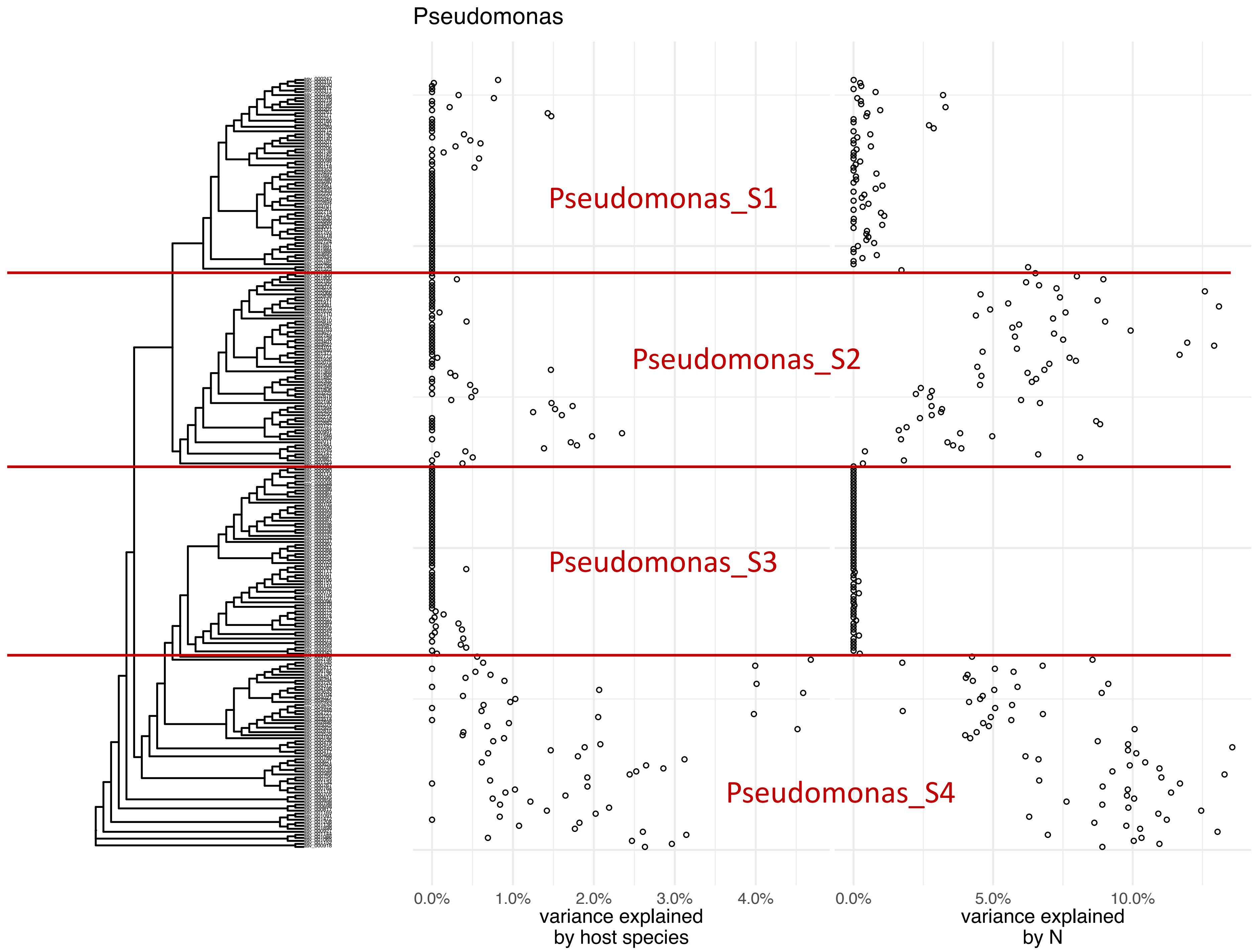
Pseudomonas
Pseudomonas_S1
Pseudomonas_S2
Pseudomonas_S3
Pseudomonas_S4
0.0%
1.0%
2.0%
3.0%
4.0%
variance explained by host species
0.0%
5.0%
10.0%
variance explained by N

RB41

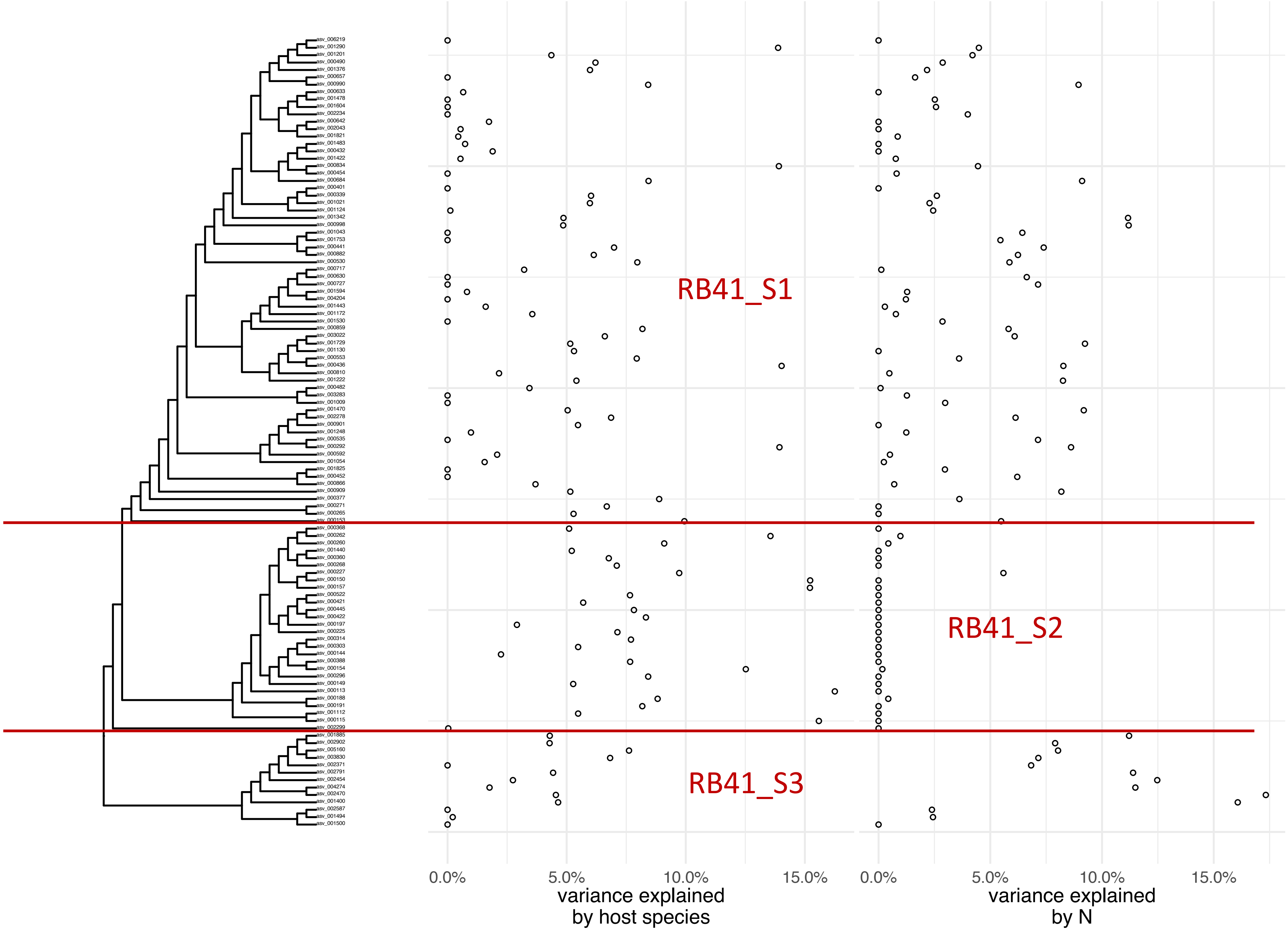
RB41_S1
RB41_S2
RB41_S3
0.0%
5.0%
10.0%
15.0%
variance explained by host species
variance explained by N

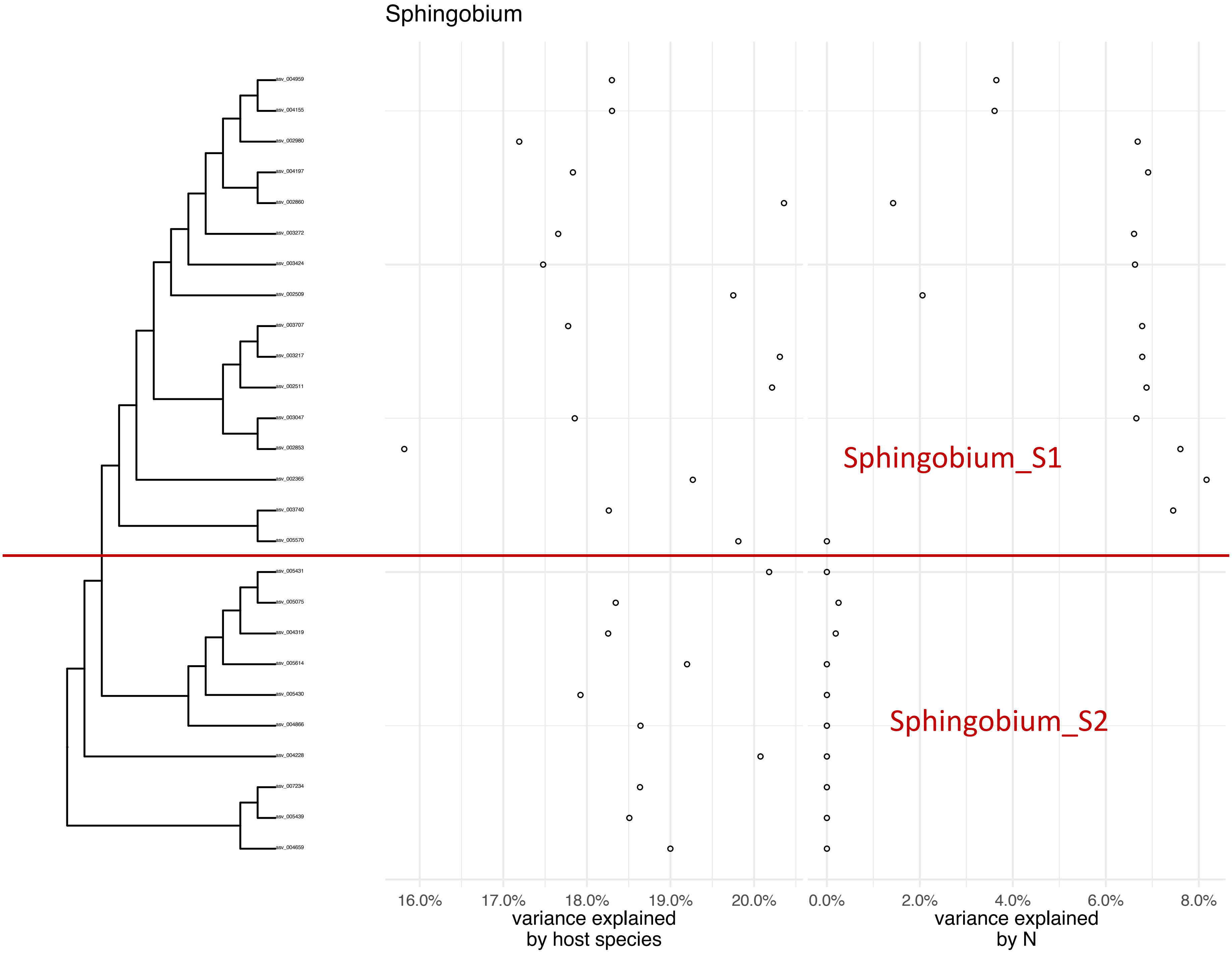

Sphingobium
asv_004959
asv_004155
asv_002980
asv_004197
asv_002860
asv_003272
asv_003424
asv_002509
asv_003707
asv_003217
asv_002511
asv_003047
asv_002853
asv_002365
asv_003740
asv_005570
asv_005431
asv_005075
asv_004319
asv_005614
asv_005430
asv_004866
asv_004228
asv_007234
asv_005439
asv_004659
Sphingobium_S1
Sphingobium_S2
16.0%
17.0%
18.0%
19.0%
20.0%
variance explained by host species
0.0%
2.0%
4.0%
6.0%
8.0%
variance explained by N

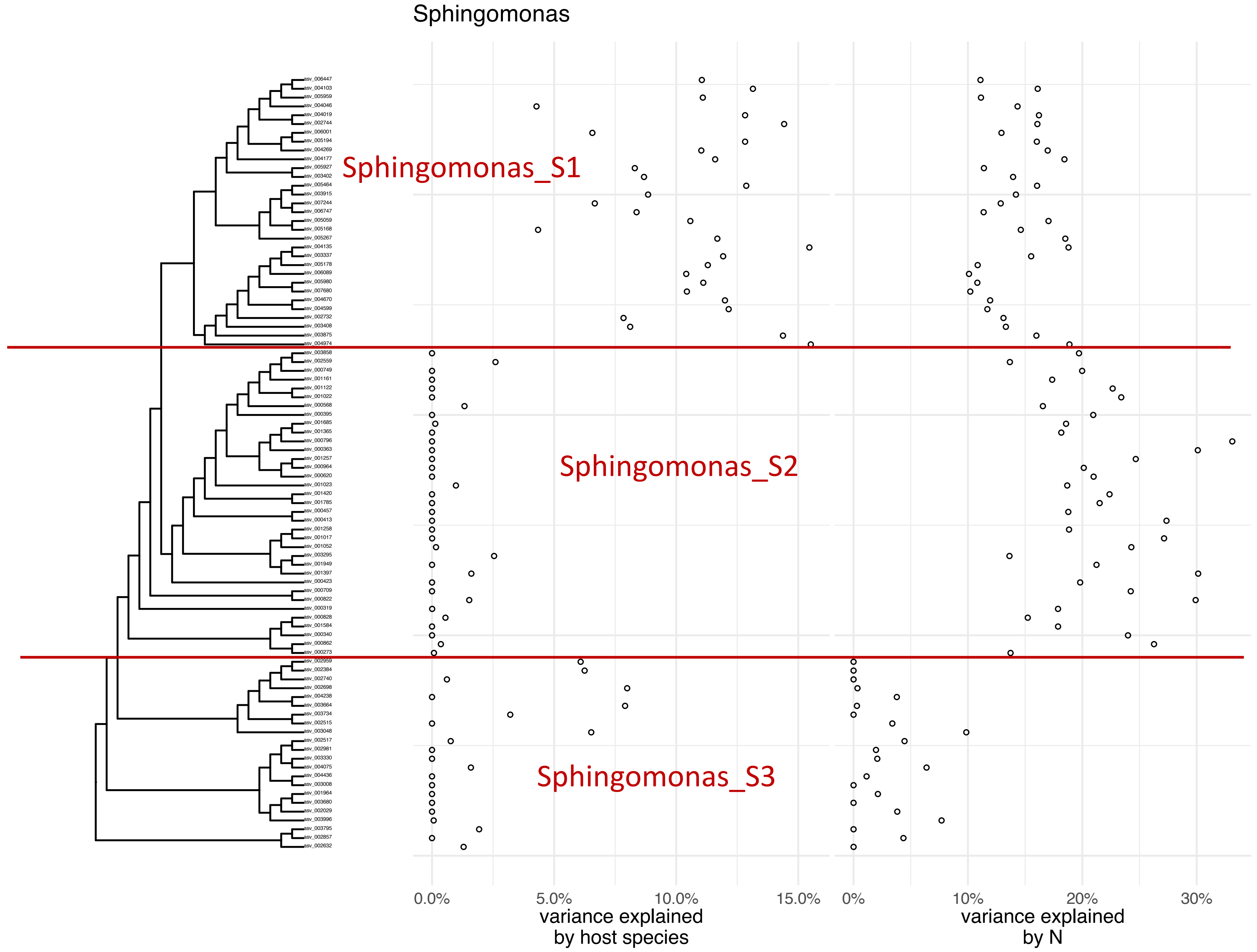
Sphingomonas
Sphingomonas_S1
Sphingomonas_S2
Sphingomonas_S3
0.0%
5.0%
10.0%
15.0%
variance explained
by host species
0%
10%
20%
30%
variance explained
by N

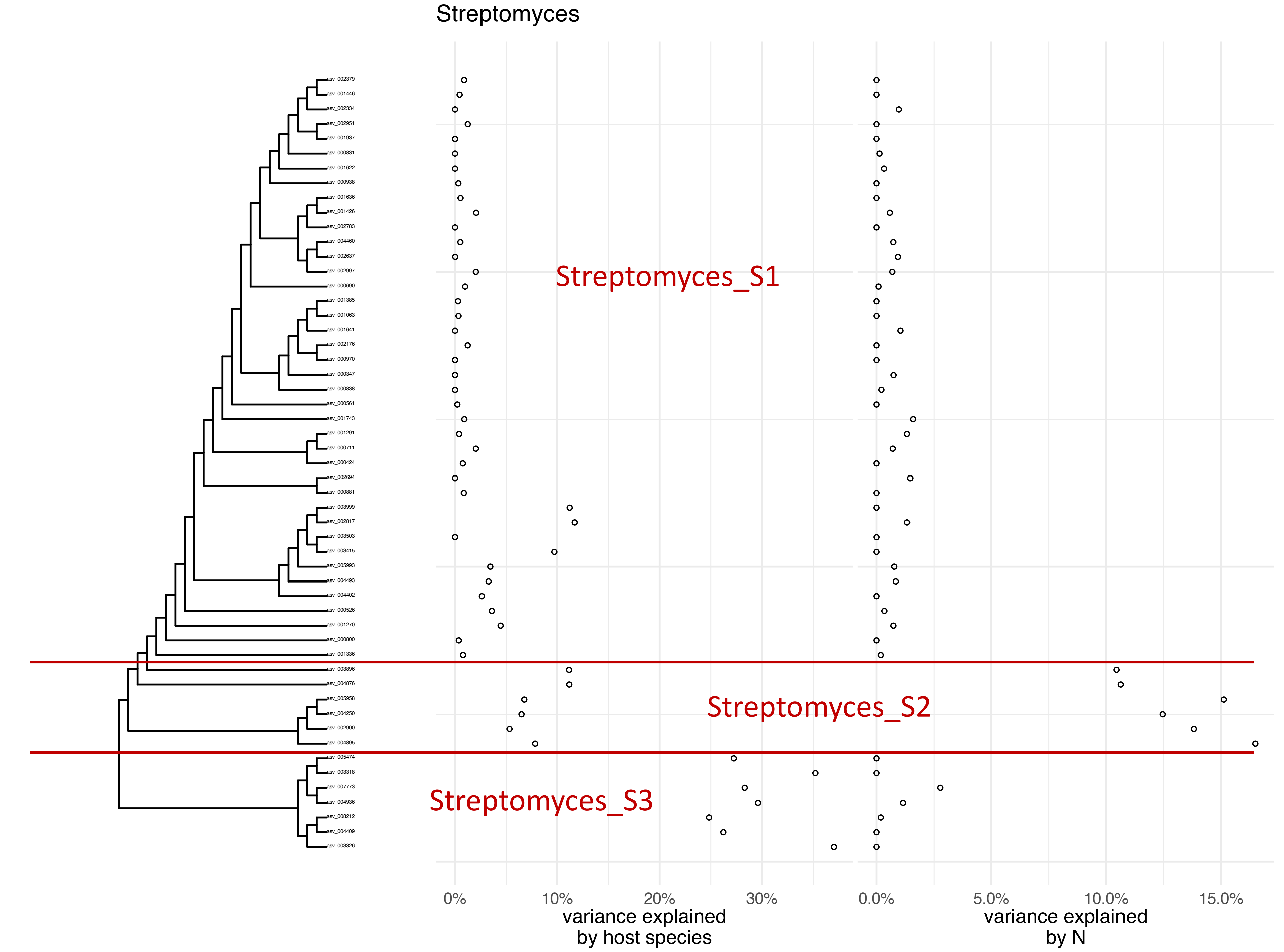

Streptomyces
Streptomyces_S1
Streptomyces_S2
Streptomyces_S3
0%
10%
20%
30%
variance explained
by host species
0.0%
5.0%
10.0%
15.0%
variance explained
by N

Genera without sub-genus groups

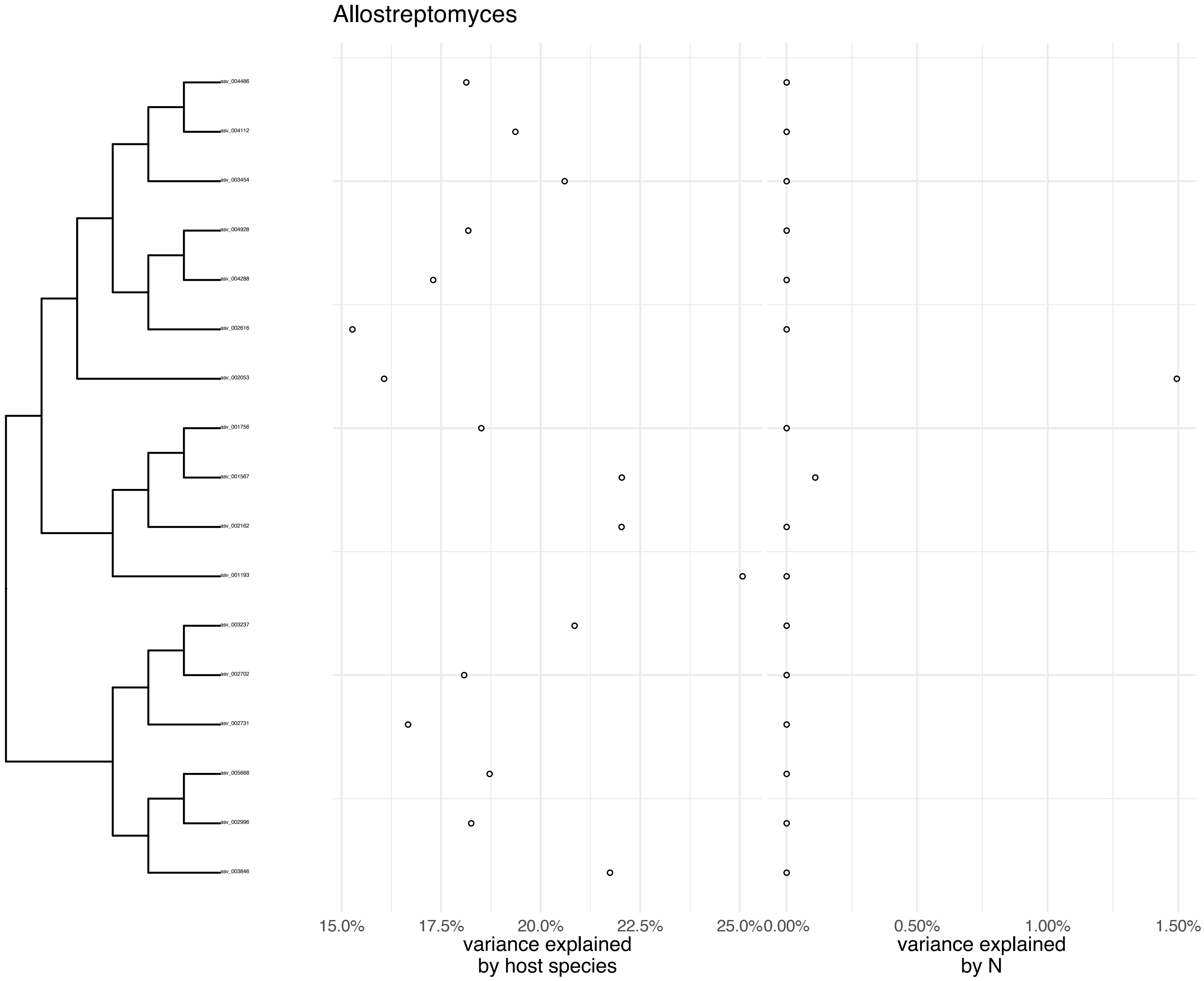

Allostreptomyces
asv_004486
asv_004112
asv_003454
asv_004928
asv_004288
asv_002616
asv_002053
asv_001756
asv_001567
asv_002162
asv_001193
asv_003237
asv_002702
asv_002731
asv_005668
asv_002996
asv_003846
15.0%
17.5%
20.0%
22.5%
25.0%
0.00%
0.50%
1.00%
1.50%
variance explained by host species
variance explained by N

Aminobacter

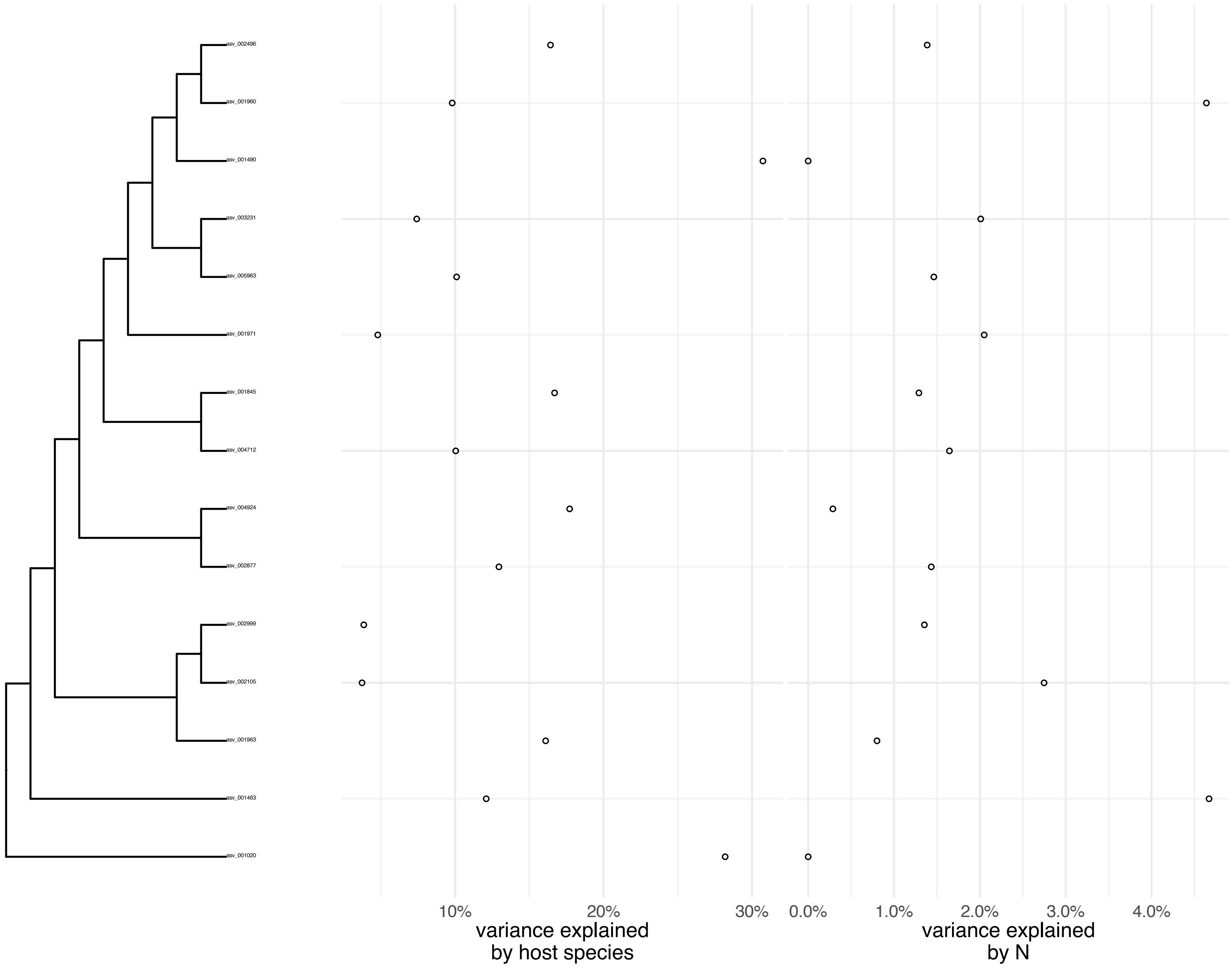

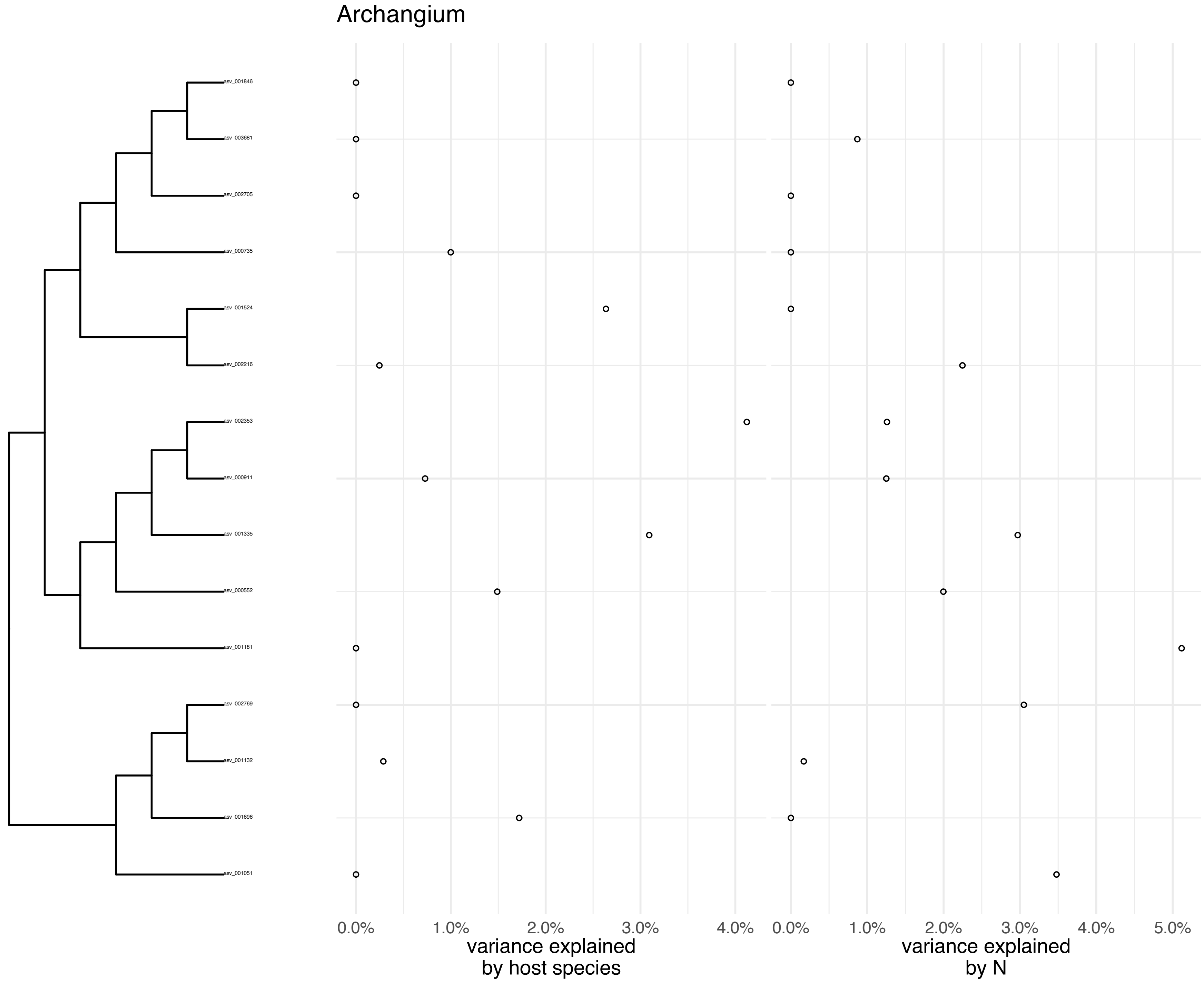

Archangium
asv_001846
asv_003681
asv_002705
asv_000735
asv_001524
asv_002216
asv_002353
asv_000911
asv_001335
asv_000552
asv_001181
asv_002769
asv_001132
asv_001696
asv_001051
0.0%
1.0%
2.0%
3.0%
4.0%
variance explained
by host species
0.0%
1.0%
2.0%
3.0%
4.0%
5.0%
variance explained
by N

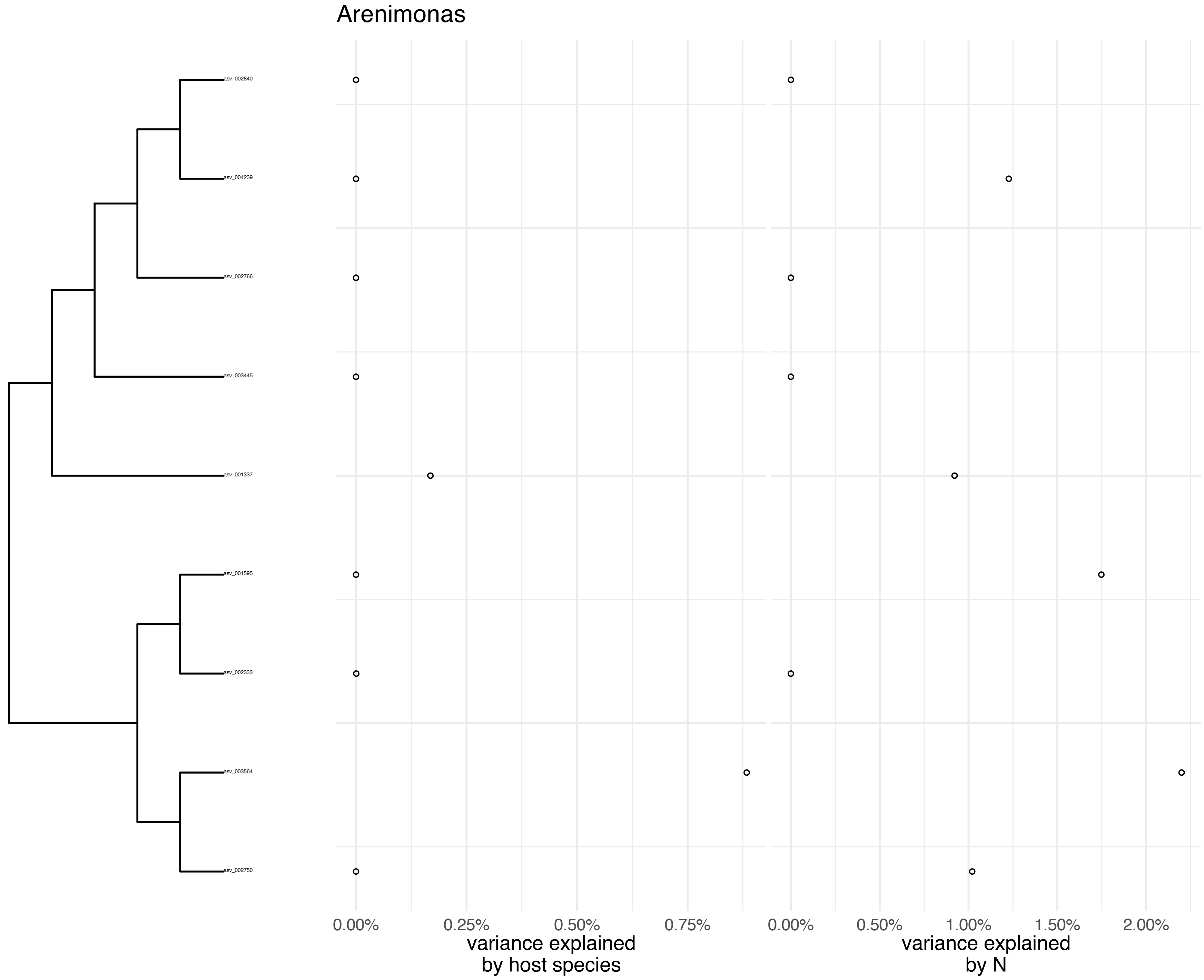

Arenimonas
asv_002840
asv_004239
asv_002766
asv_003445
asv_001337
asv_001595
asv_002333
asv_003564
asv_002750
0.00%
0.25%
0.50%
0.75%
variance explained by host species
0.00%
0.50%
1.00%
1.50%
2.00%
variance explained by N

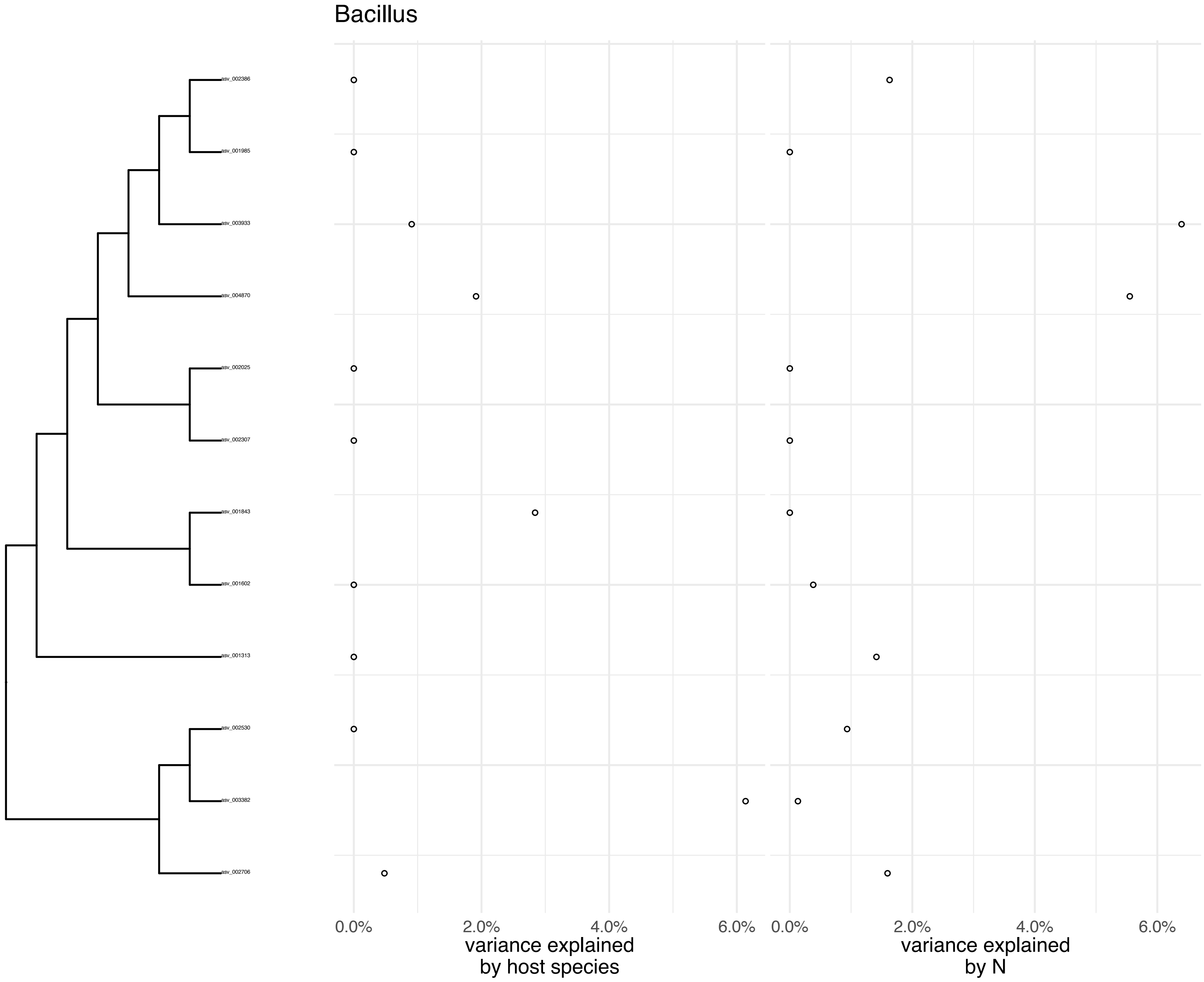

Bacillus
asv_002386
asv_001985
asv_003933
asv_004870
asv_002025
asv_002307
asv_001843
asv_001602
asv_001313
asv_002530
asv_003382
asv_002706
0.0%
2.0%
4.0%
6.0%
variance explained by host species
0.0%
2.0%
4.0%
6.0%
variance explained by N

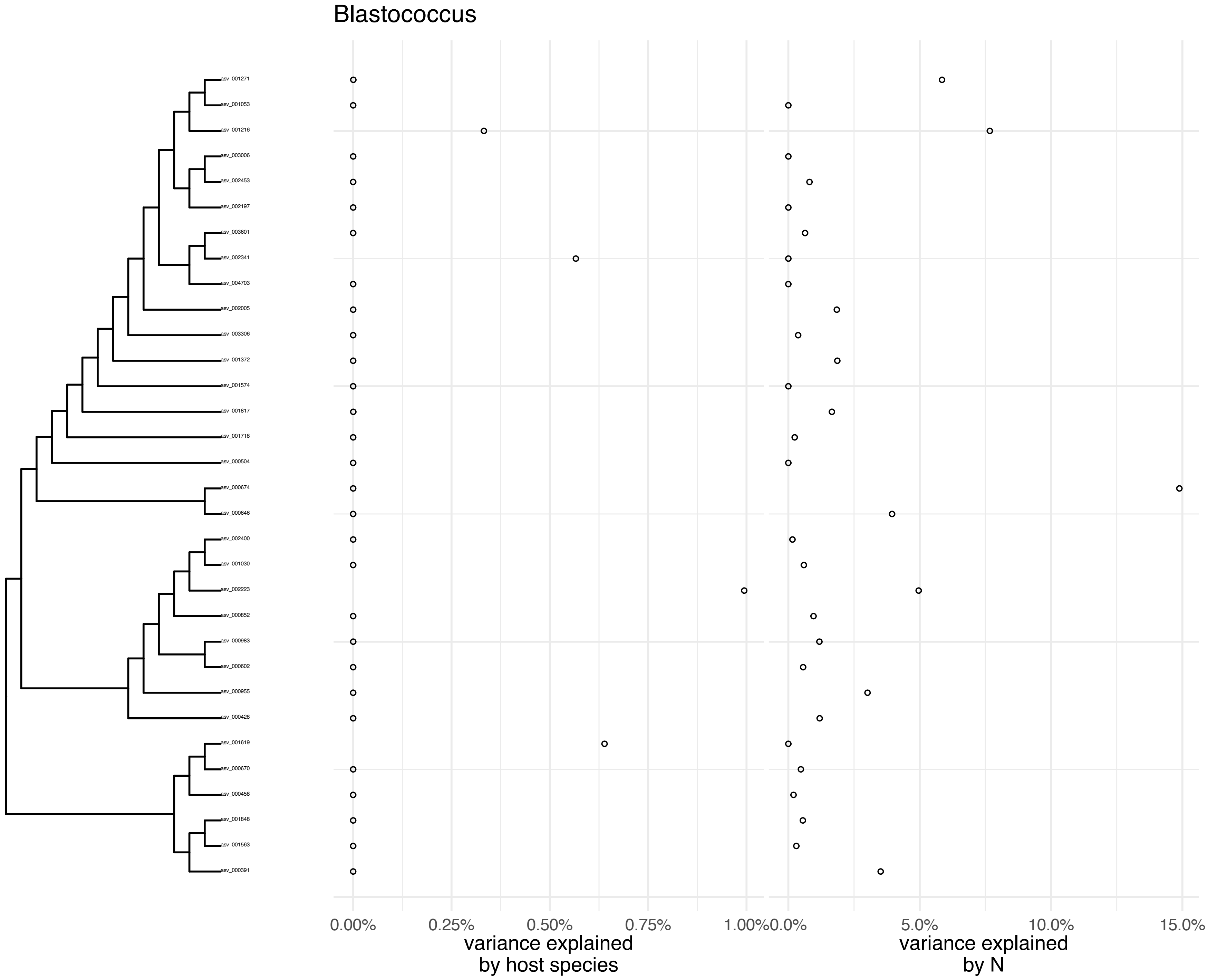
Blastococcus
asv_001271
asv_001053
asv_001216
asv_003006
asv_002453
asv_002197
asv_003601
asv_002341
asv_004703
asv_002005
asv_003306
asv_001372
asv_001574
asv_001817
asv_001718
asv_000504
asv_000674
asv_000646
asv_002400
asv_001030
asv_002223
asv_000852
asv_000983
asv_000602
asv_000955
asv_000428
asv_001619
asv_000670
asv_000458
asv_001848
asv_001563
asv_000391
0.00%
0.25%
0.50%
0.75%
1.00%
variance explained by host species
0.0%
5.0%
10.0%
15.0%
variance explained by N

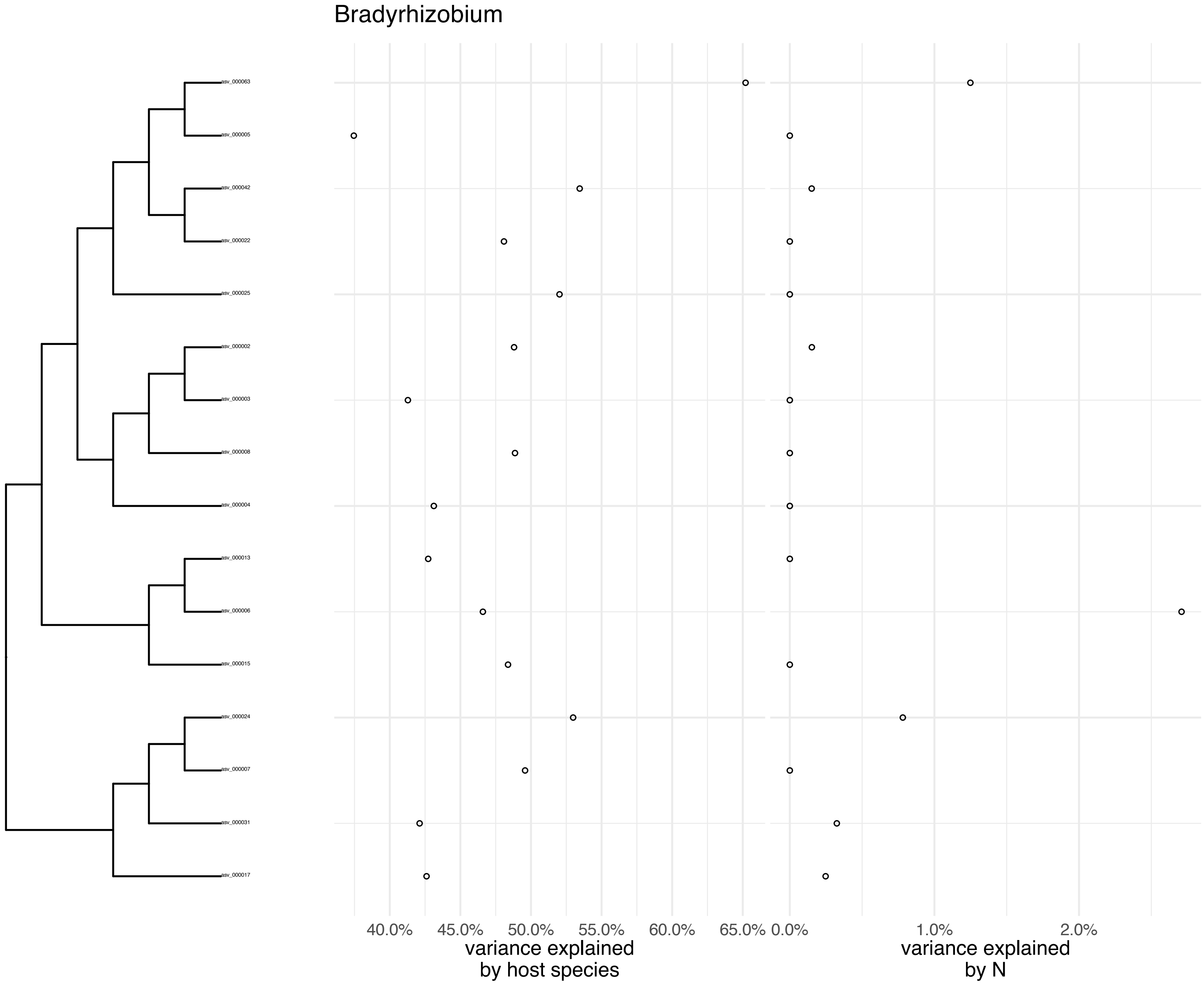
Bradyrhizobium
asv_000063
asv_000005
asv_000042
asv_000022
asv_000025
asv_000002
asv_000003
asv_000008
asv_000004
asv_000013
asv_000006
asv_000015
asv_000024
asv_000007
asv_000031
asv_000017
40.0%
45.0%
50.0%
55.0%
60.0%
65.0%
variance explained
by host species
0.0%
1.0%
2.0%
variance explained
by N

Candidatus_Nitrocosmicus

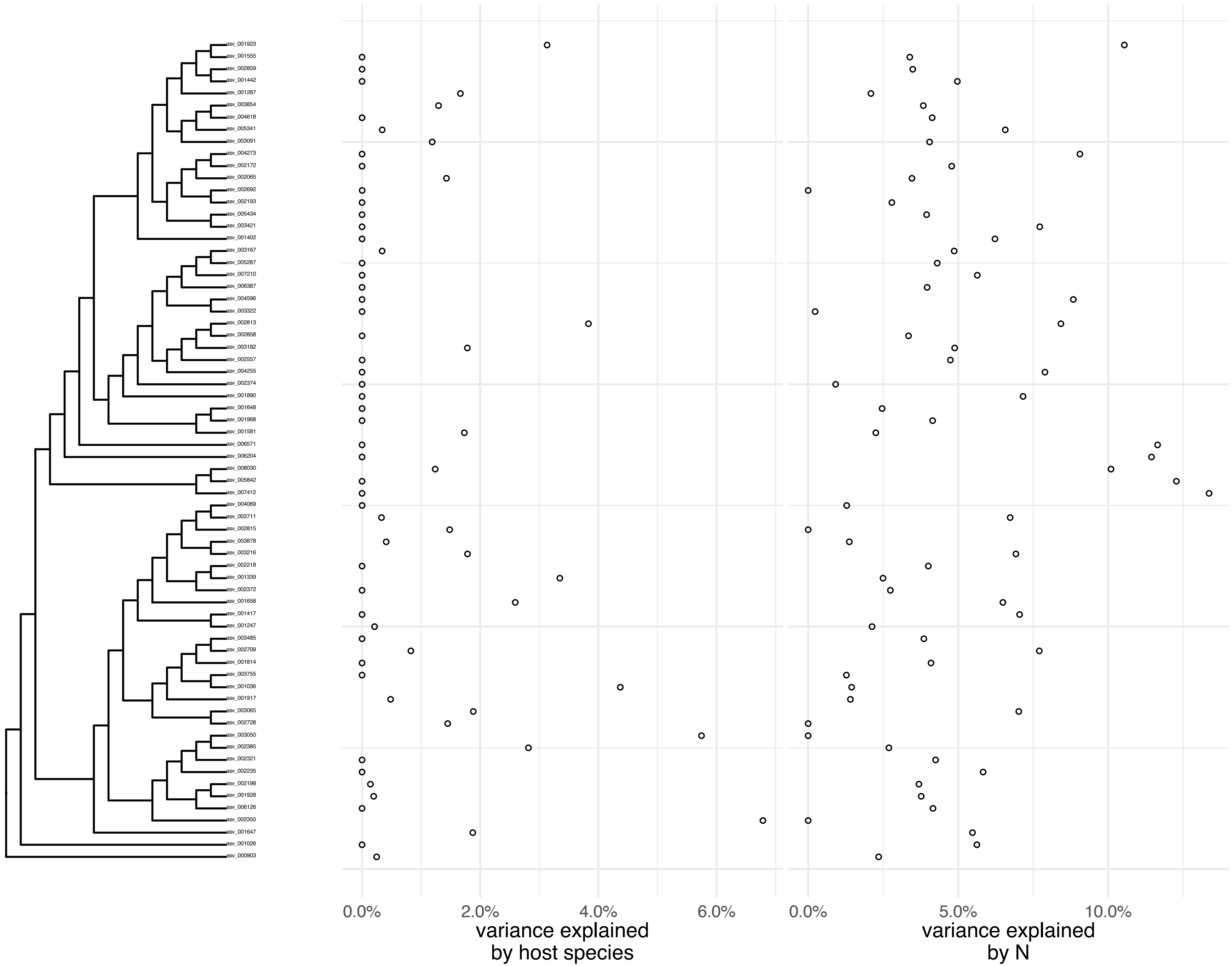

Candidatus_Udaeobacter

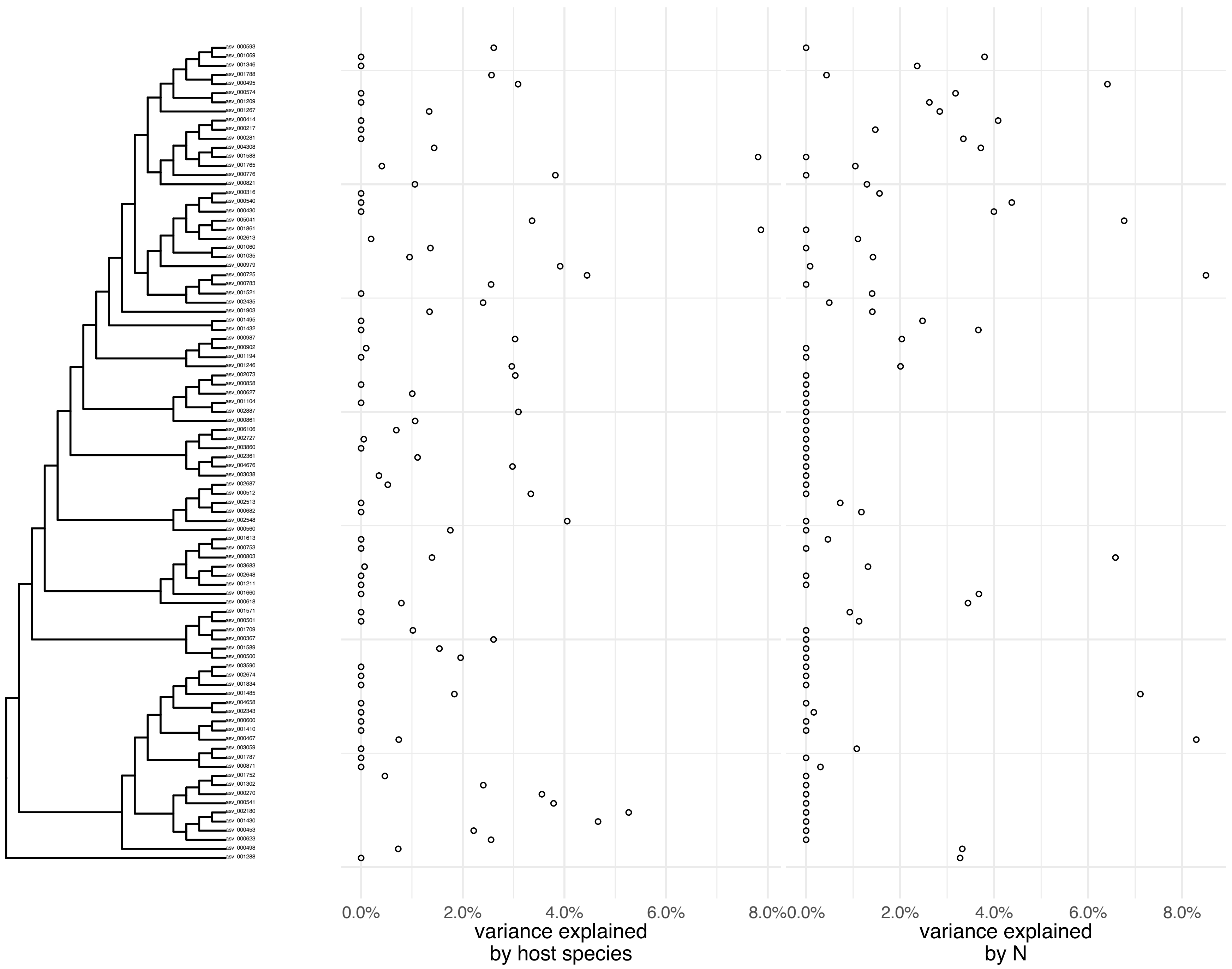

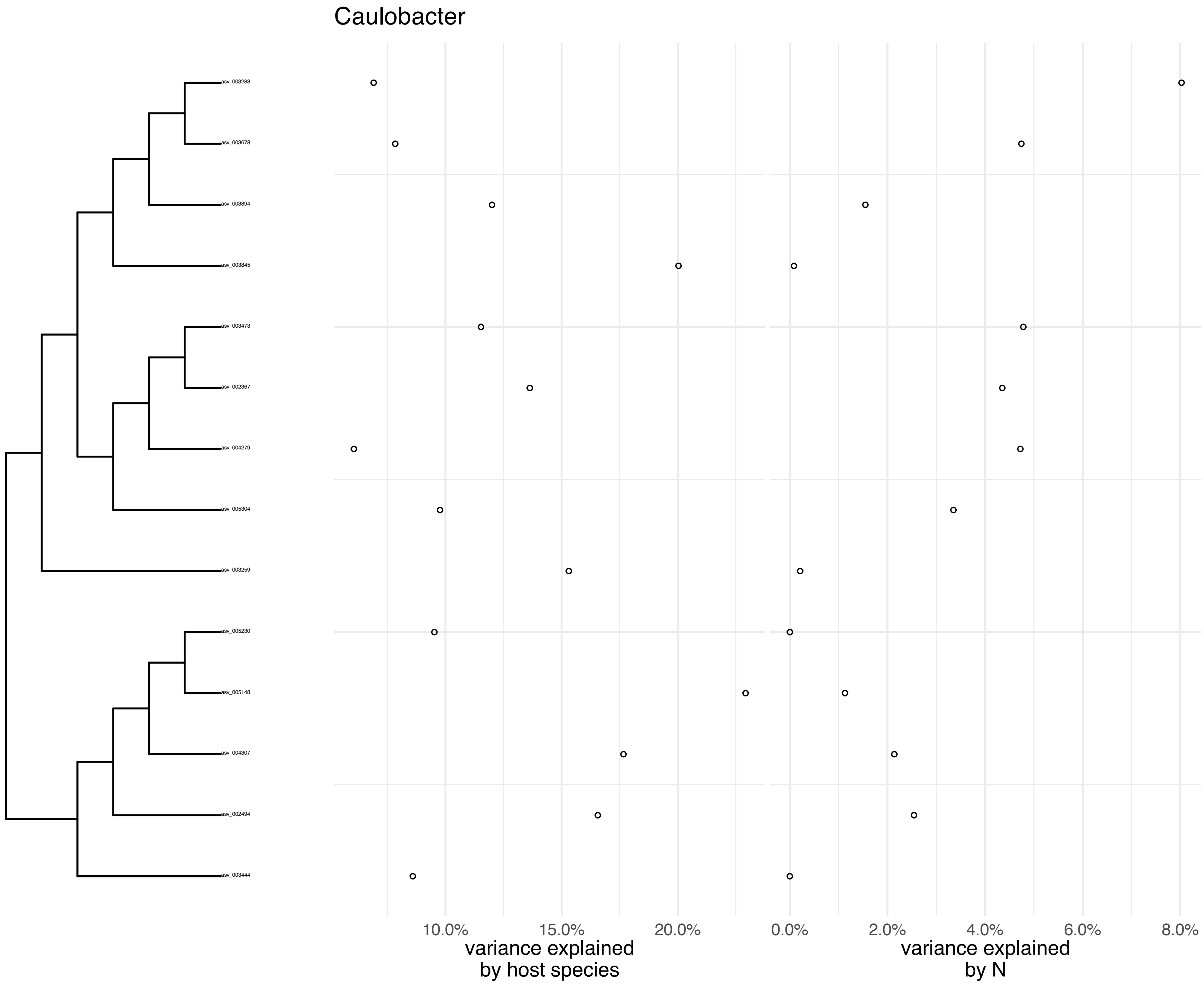

Caulobacter
asv_003288
asv_003678
asv_003894
asv_003845
asv_003473
asv_002367
asv_004279
asv_005304
asv_003259
asv_005230
asv_005148
asv_004307
asv_002494
asv_003444
10.0%
15.0%
20.0%
variance explained
by host species
0.0%
2.0%
4.0%
6.0%
8.0%
variance explained
by N

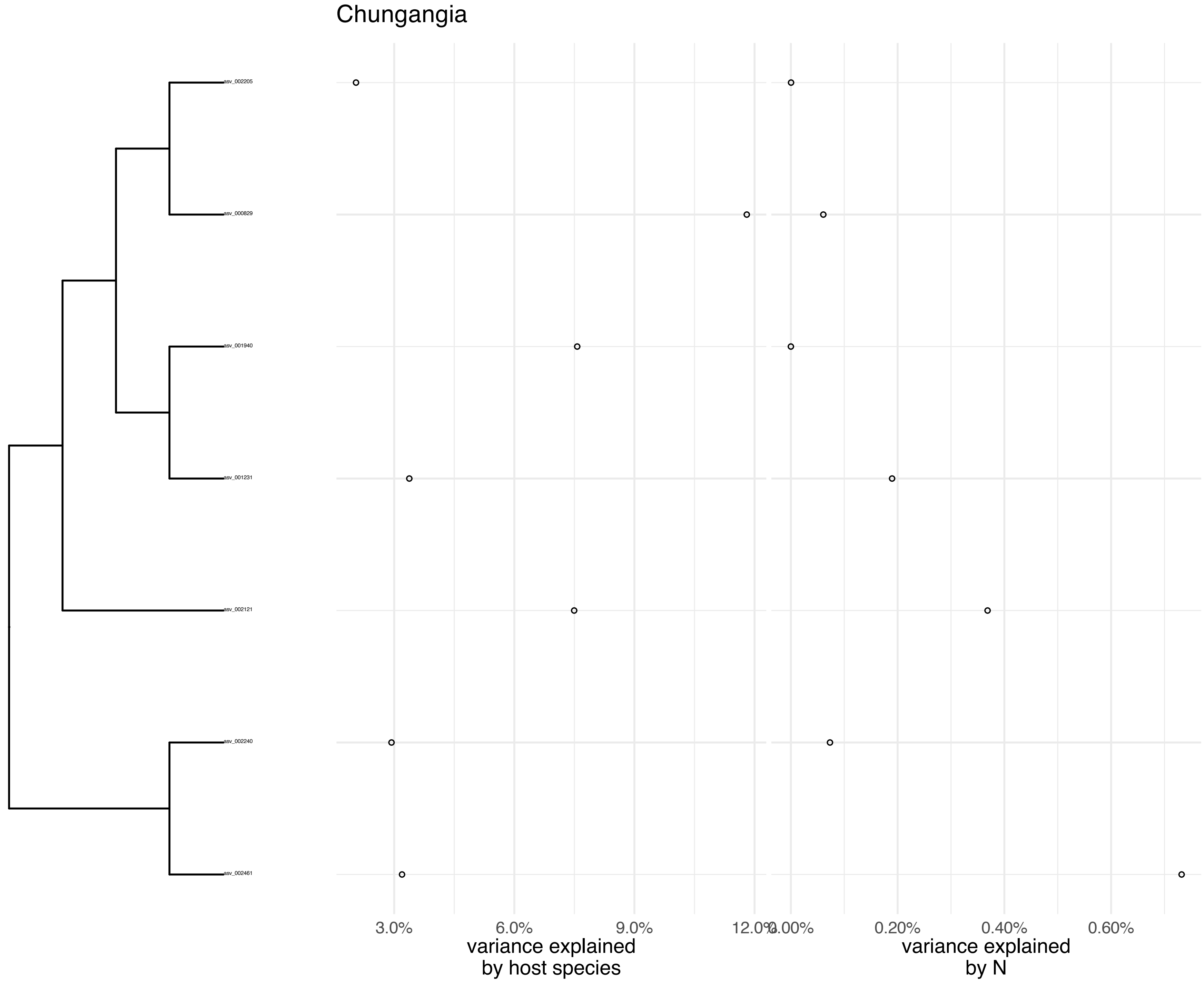

Chungangia
asv_002205
asv_000829
asv_001940
asv_001231
asv_002121
asv_002240
asv_002461
3.0%
6.0%
9.0%
12.0%
0.00%
0.20%
0.40%
0.60%
variance explained by host species
variance explained by N

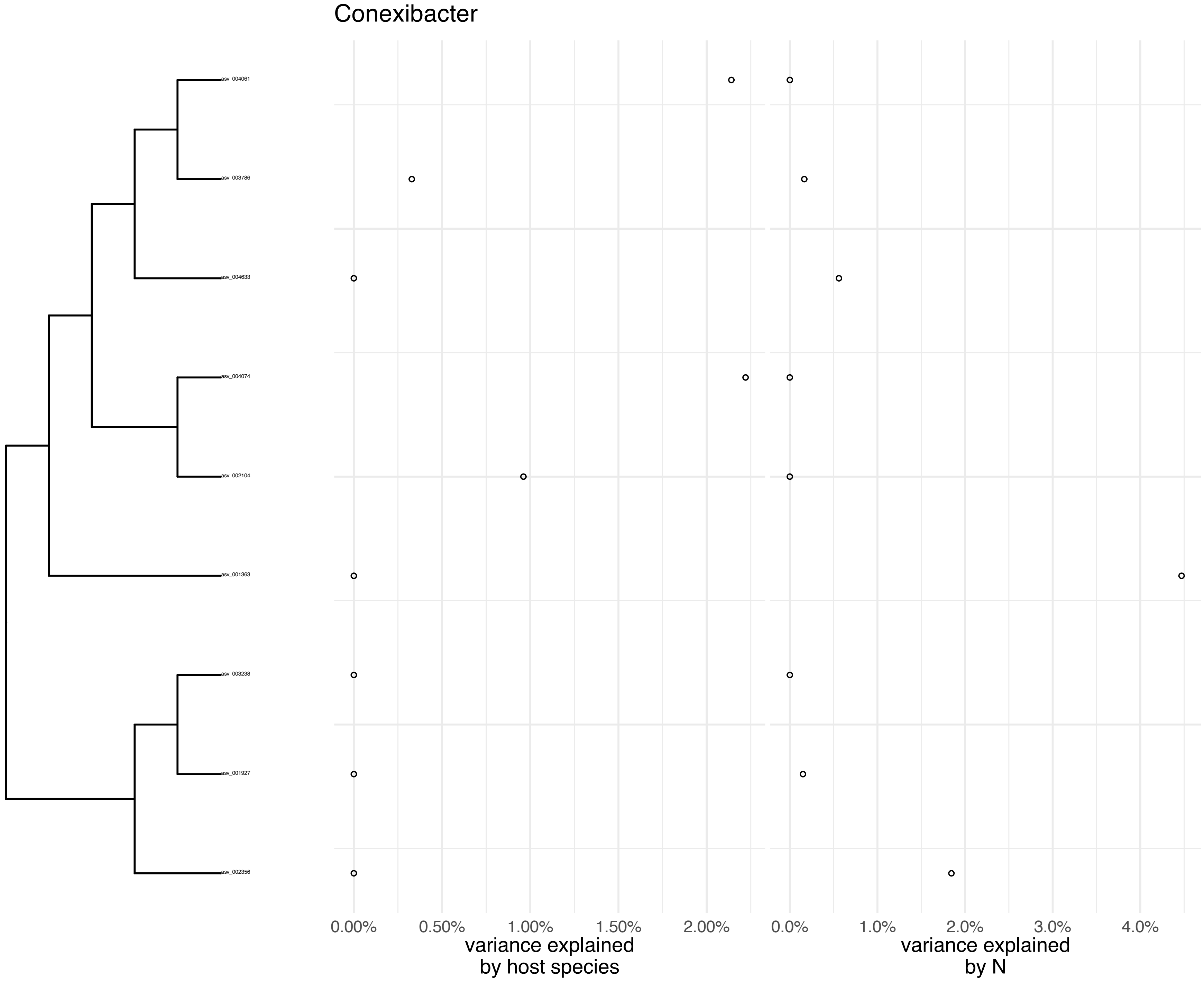
Conexibacter
asv_004061
asv_003786
asv_004633
asv_004074
asv_002104
asv_001363
asv_003238
asv_001927
asv_002356
0.00%
0.50%
1.00%
1.50%
2.00%
variance explained
by host species
0.0%
1.0%
2.0%
3.0%
4.0%
variance explained
by N

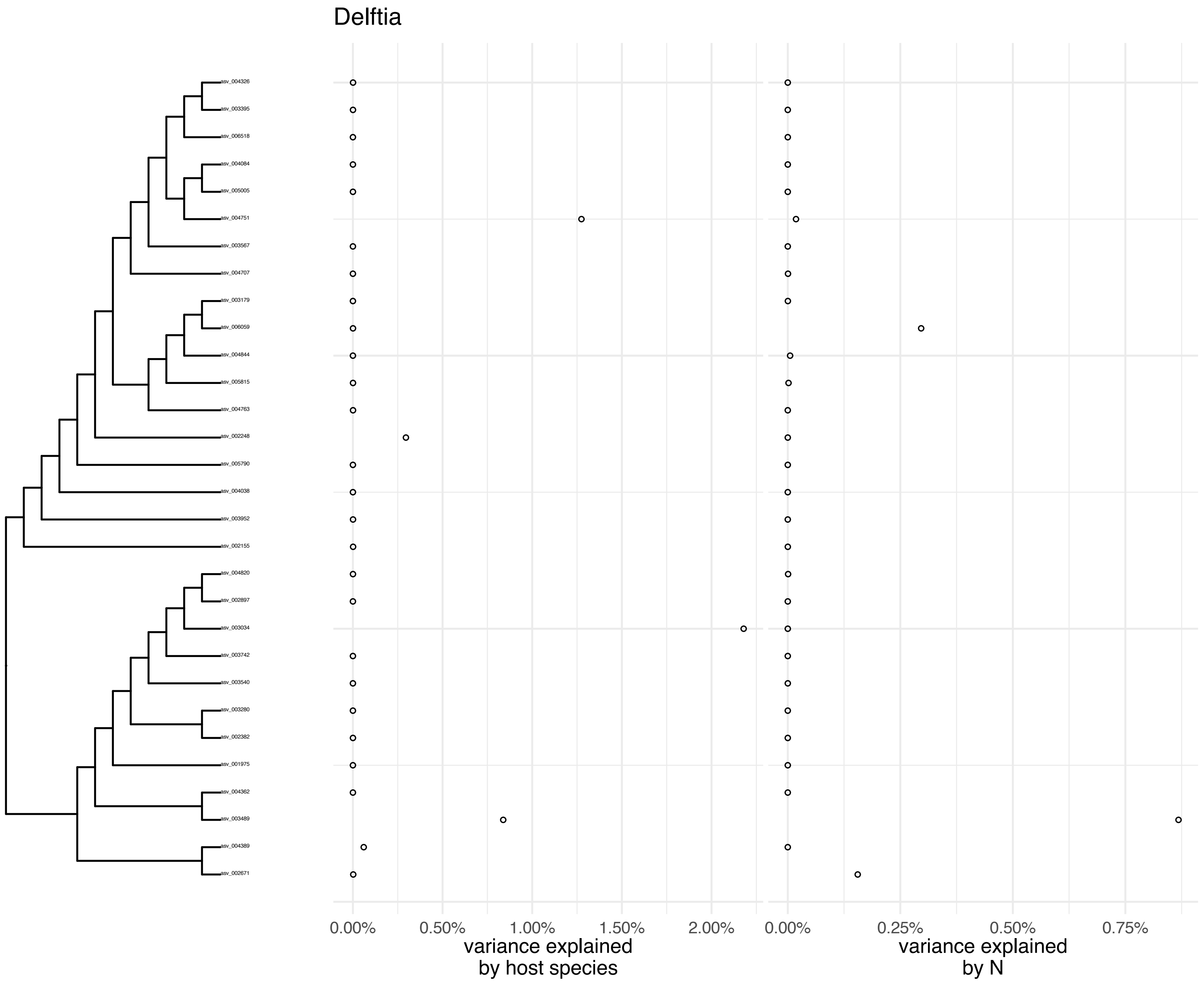
Delftia
asv_004326
asv_003395
asv_006518
asv_004084
asv_005005
asv_004751
asv_003567
asv_004707
asv_003179
asv_006059
asv_004844
asv_005815
asv_004763
asv_002248
asv_005790
asv_004038
asv_003952
asv_002155
asv_004820
asv_002897
asv_003034
asv_003742
asv_003540
asv_003280
asv_002382
asv_001975
asv_004362
asv_003489
asv_004389
asv_002671
0.00%
0.50%
1.00%
1.50%
2.00%
variance explained by host species
0.00%
0.25%
0.50%
0.75%
variance explained by N

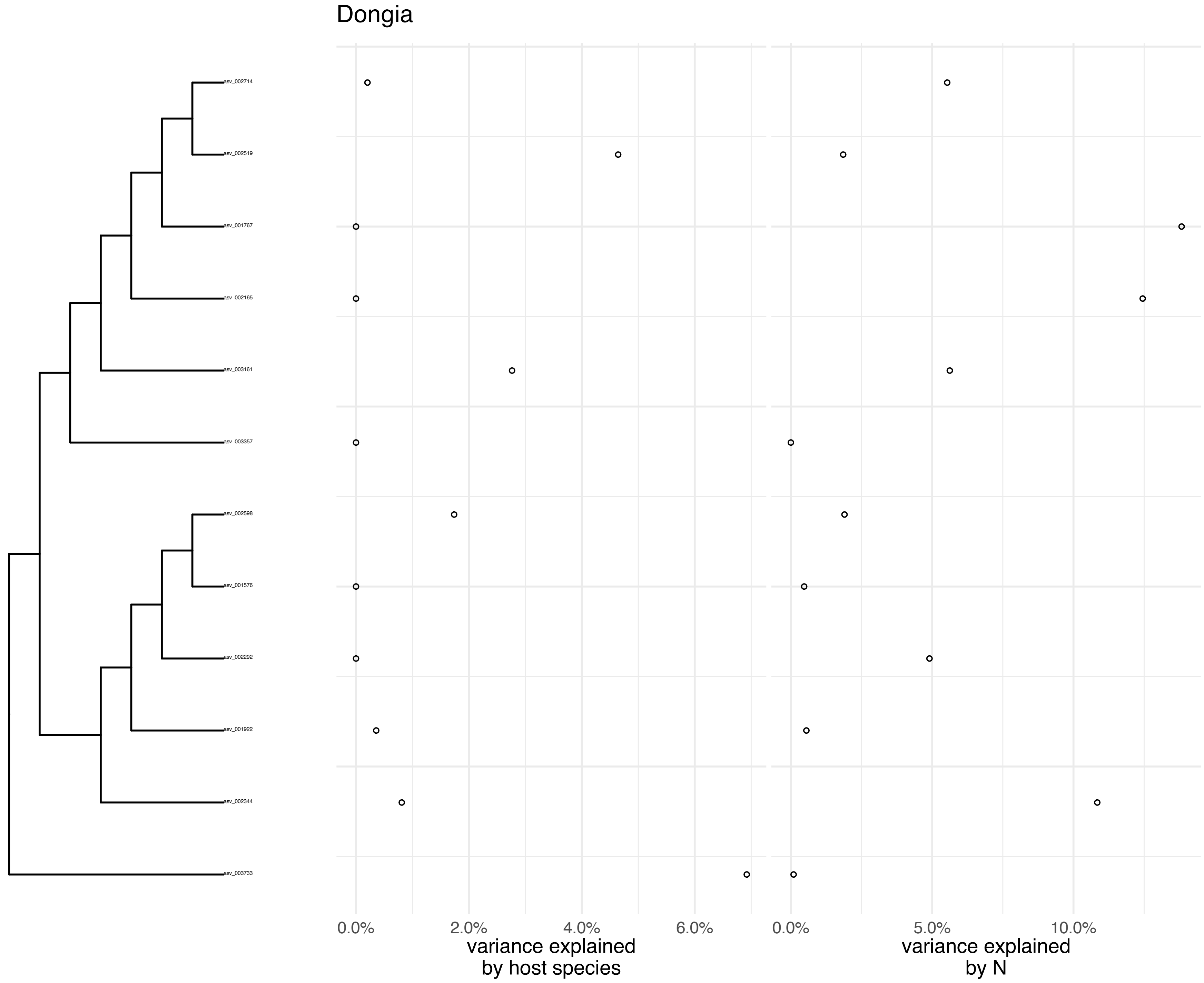
Dongia
asv_002714
asv_002519
asv_001767
asv_002165
asv_003161
asv_003357
asv_002598
asv_001576
asv_002292
asv_001922
asv_002344
asv_003733
0.0%
2.0%
4.0%
6.0%
variance explained
by host species
0.0%
5.0%
10.0%
variance explained
by N

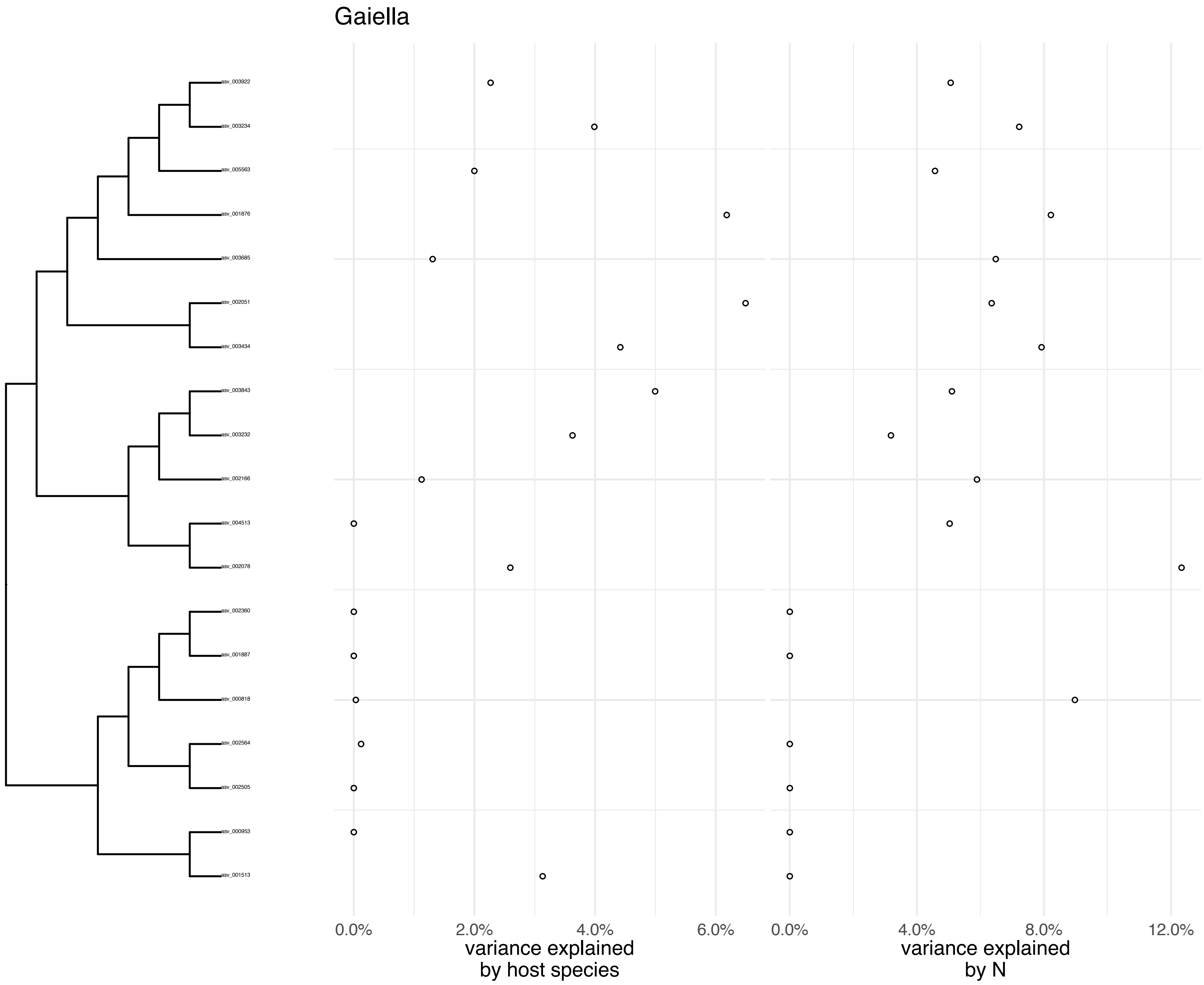

Gaiella
asv_003922
asv_003234
asv_005563
asv_001876
asv_003685
asv_002051
asv_003434
asv_003843
asv_003232
asv_002166
asv_004513
asv_002078
asv_002360
asv_001887
asv_000818
asv_002564
asv_002505
asv_000953
asv_001513
0.0%
2.0%
4.0%
6.0%
variance explained by host species
0.0%
4.0%
8.0%
12.0%
variance explained by N

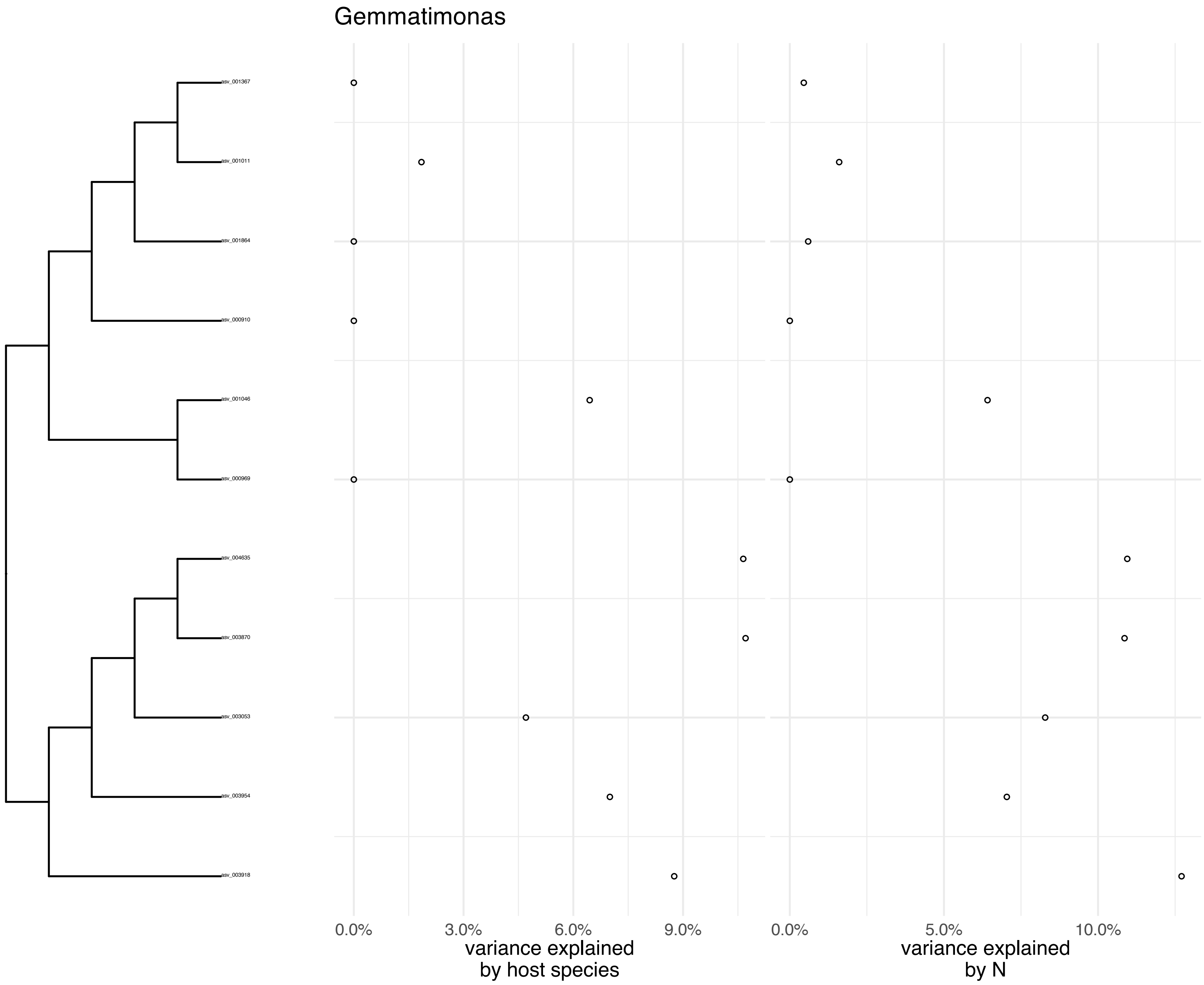
Gemmatimonas
asv_001367
asv_001011
asv_001864
asv_000910
asv_001046
asv_000969
asv_004635
asv_003870
asv_003053
asv_003954
asv_003918
0.0%
3.0%
6.0%
9.0%
variance explained by host species
0.0%
5.0%
10.0%
variance explained by N

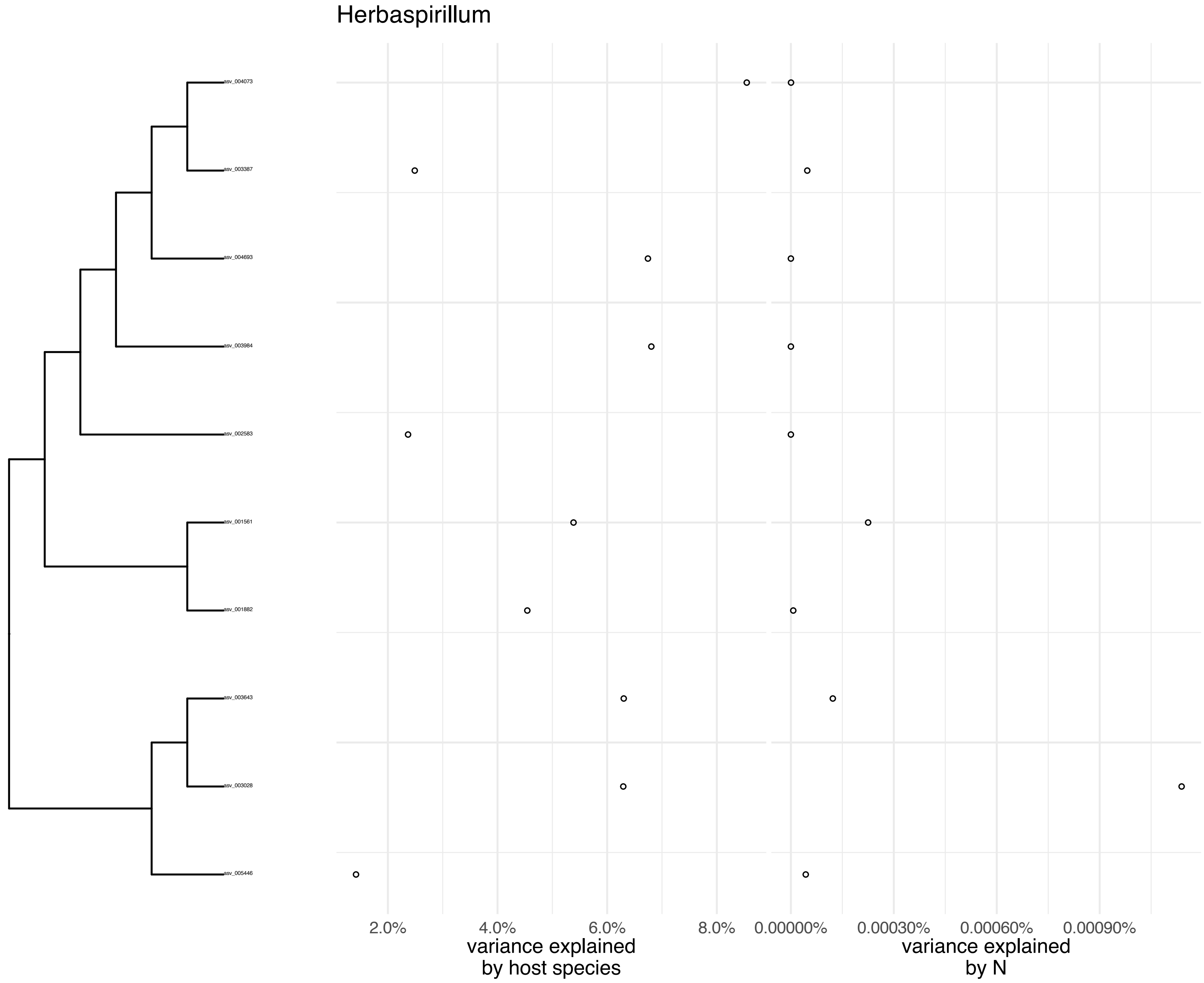
Herbaspirillum
asv_004073
asv_003387
asv_004693
asv_003984
asv_002583
asv_001561
asv_001882
asv_003643
asv_003028
asv_005446
2.0%
4.0%
6.0%
8.0%
0.00000%
0.00030%
0.00060%
0.00090%
variance explained by host species
variance explained by N

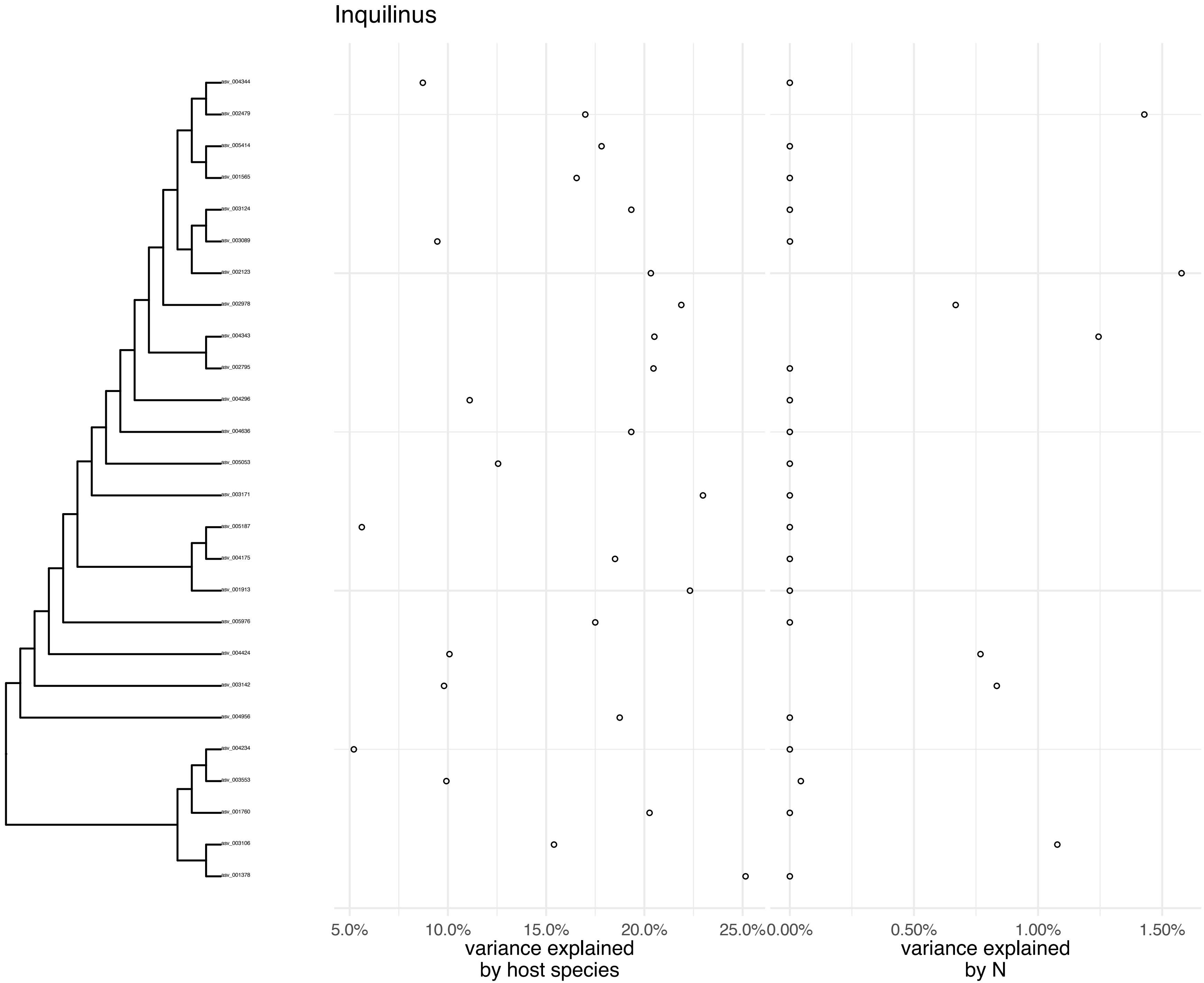
Inquilinus
asv_004344
asv_002479
asv_005414
asv_001565
asv_003124
asv_003089
asv_002123
asv_002978
asv_004343
asv_002795
asv_004296
asv_004636
asv_005053
asv_003171
asv_005187
asv_004175
asv_001913
asv_005976
asv_004424
asv_003142
asv_004956
asv_004234
asv_003553
asv_001760
asv_003106
asv_001378
5.0%
10.0%
15.0%
20.0%
25.0%
variance explained
by host species
0.00%
0.50%
1.00%
1.50%
variance explained
by N

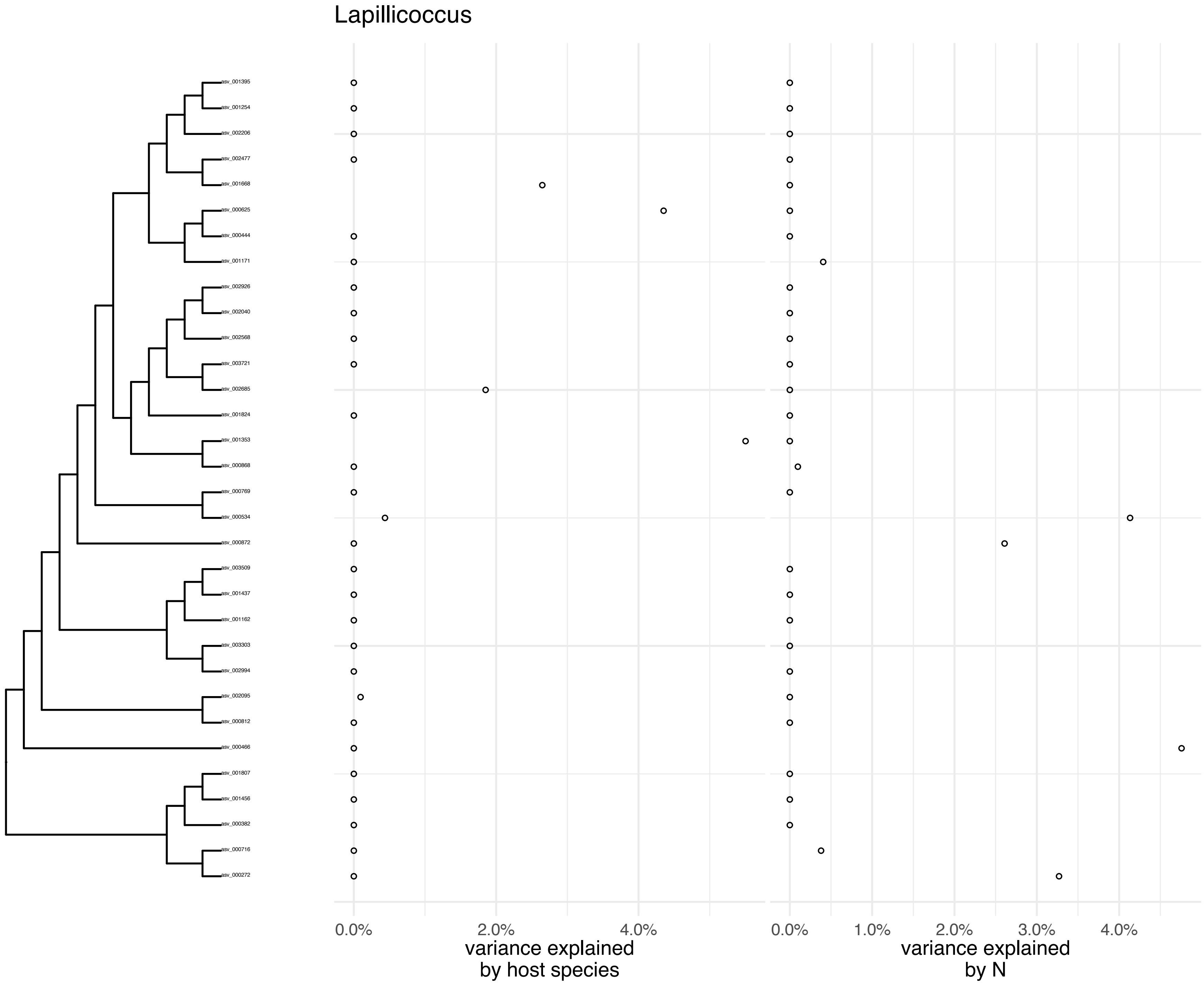

Lapillicoccus
asv_001395
asv_001254
asv_002206
asv_002477
asv_001668
asv_000625
asv_000444
asv_001171
asv_002926
asv_002040
asv_002568
asv_003721
asv_002685
asv_001824
asv_001353
asv_000868
asv_000769
asv_000534
asv_000872
asv_003509
asv_001437
asv_001162
asv_003303
asv_002994
asv_002095
asv_000812
asv_000466
asv_001807
asv_001456
asv_000382
asv_000716
asv_000272
0.0%
2.0%
4.0%
variance explained by host species
0.0%
1.0%
2.0%
3.0%
4.0%
variance explained by N

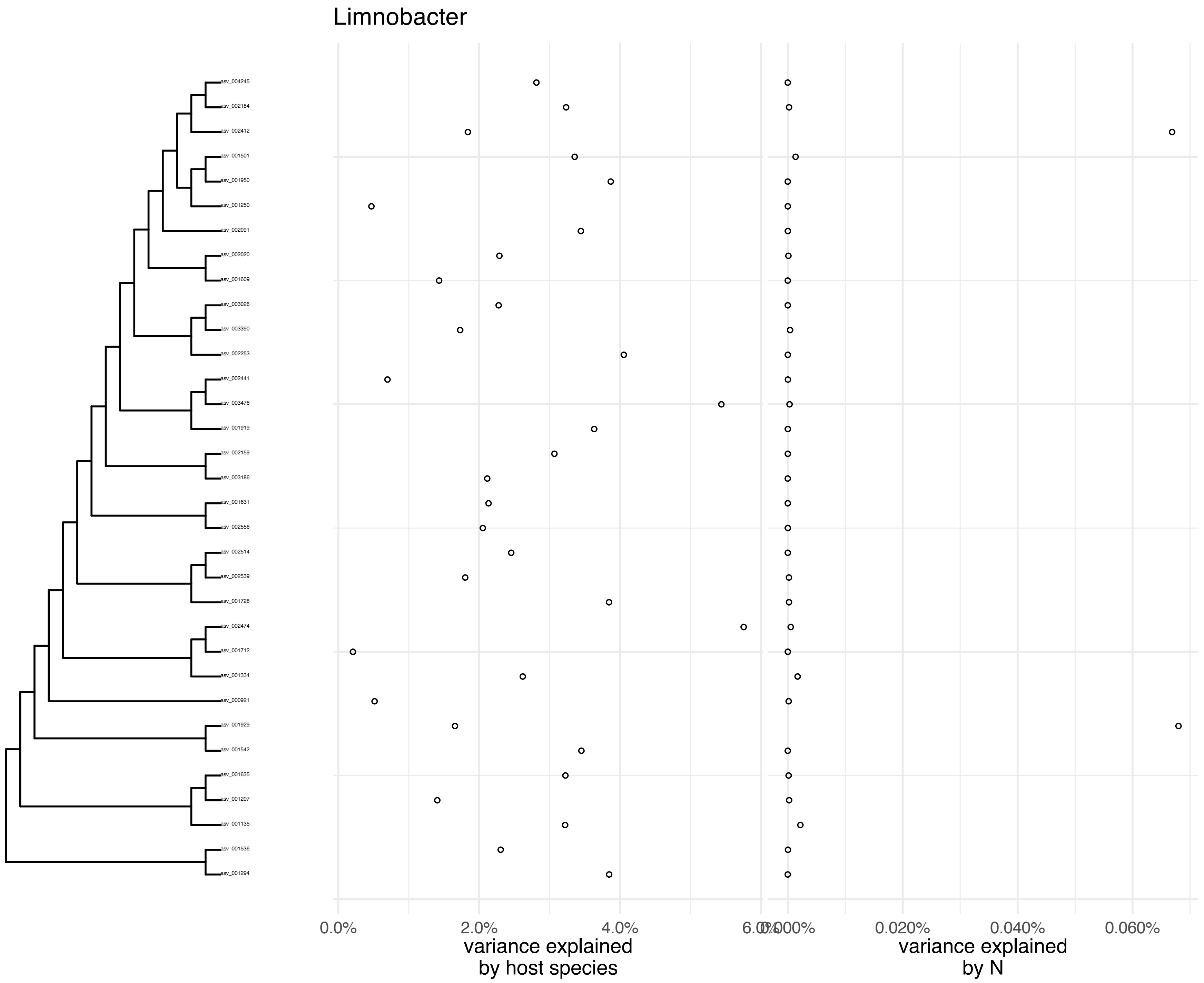
Limnobacter
asv_004245
asv_002184
asv_002412
asv_001501
asv_001950
asv_001250
asv_002091
asv_002020
asv_001609
asv_003026
asv_003390
asv_002253
asv_002441
asv_003476
asv_001919
asv_002159
asv_003186
asv_001631
asv_002556
asv_002514
asv_002539
asv_001728
asv_002474
asv_001712
asv_001334
asv_000921
asv_001929
asv_001542
asv_001635
asv_001207
asv_001135
asv_001536
asv_001294
0.0%
2.0%
4.0%
6.0%
variance explained
by host species
0.000%
0.020%
0.040%
0.060%
variance explained
by N

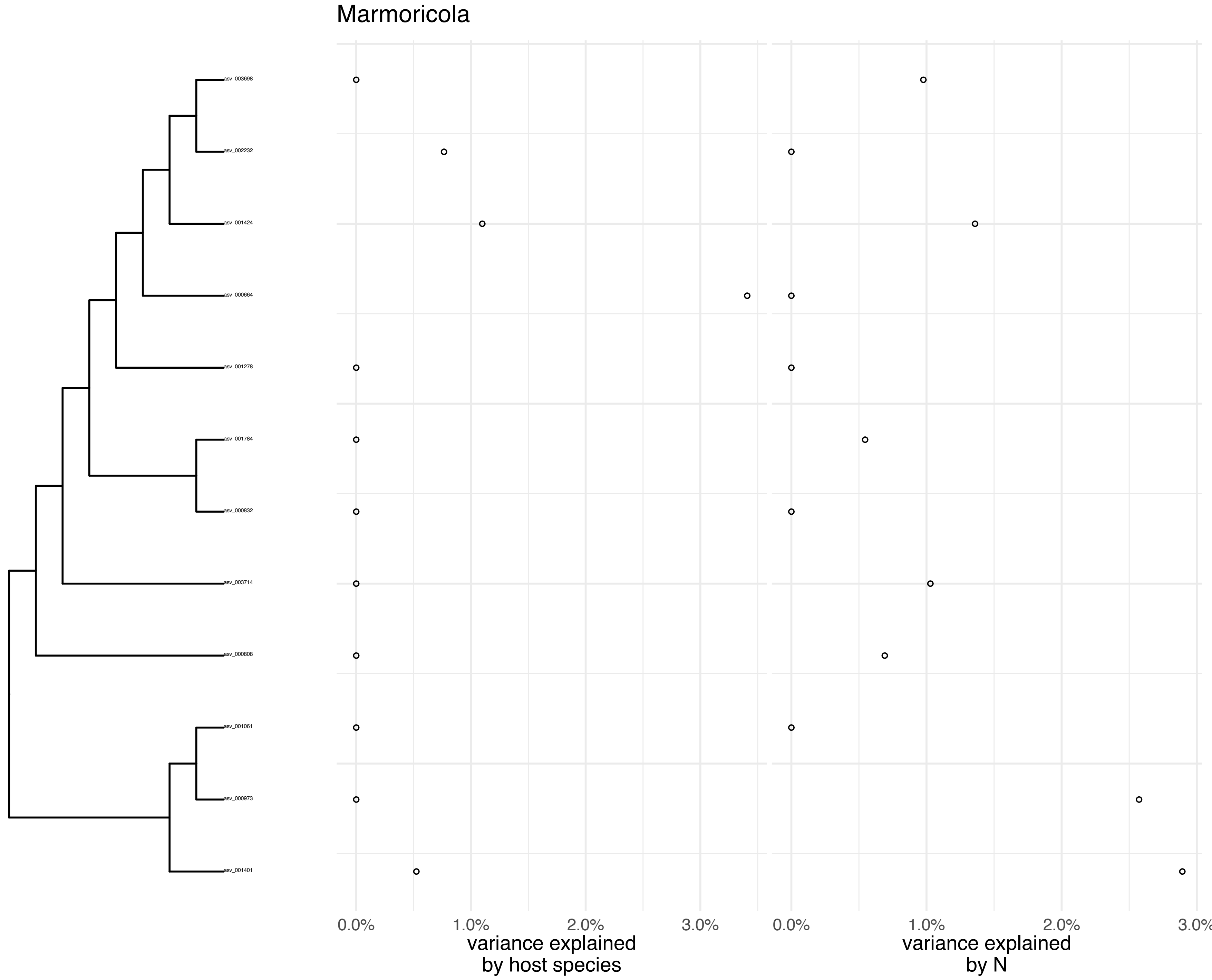

Marmoricola
asv_003698
asv_002232
asv_001424
asv_000664
asv_001278
asv_001784
asv_000832
asv_003714
asv_000808
asv_001061
asv_000973
asv_001401
0.0%
1.0%
2.0%
3.0%
variance explained by host species
0.0%
1.0%
2.0%
3.0%
variance explained by N

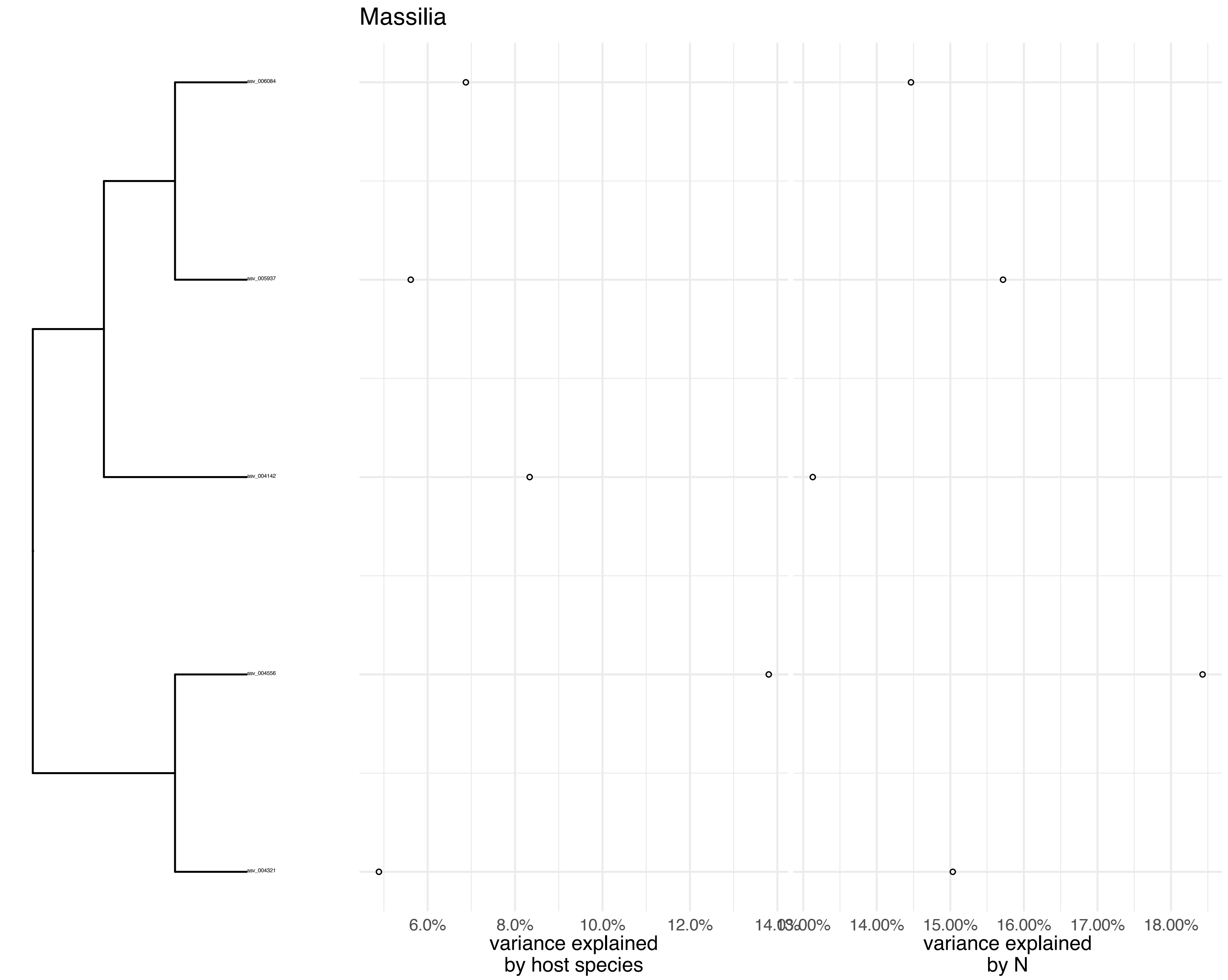
Massilia
asv_006084
asv_005937
asv_004142
asv_004556
asv_004321
6.0%
8.0%
10.0%
12.0%
14.0%
variance explained by host species
13.00%
14.00%
15.00%
16.00%
17.00%
18.00%
variance explained by N

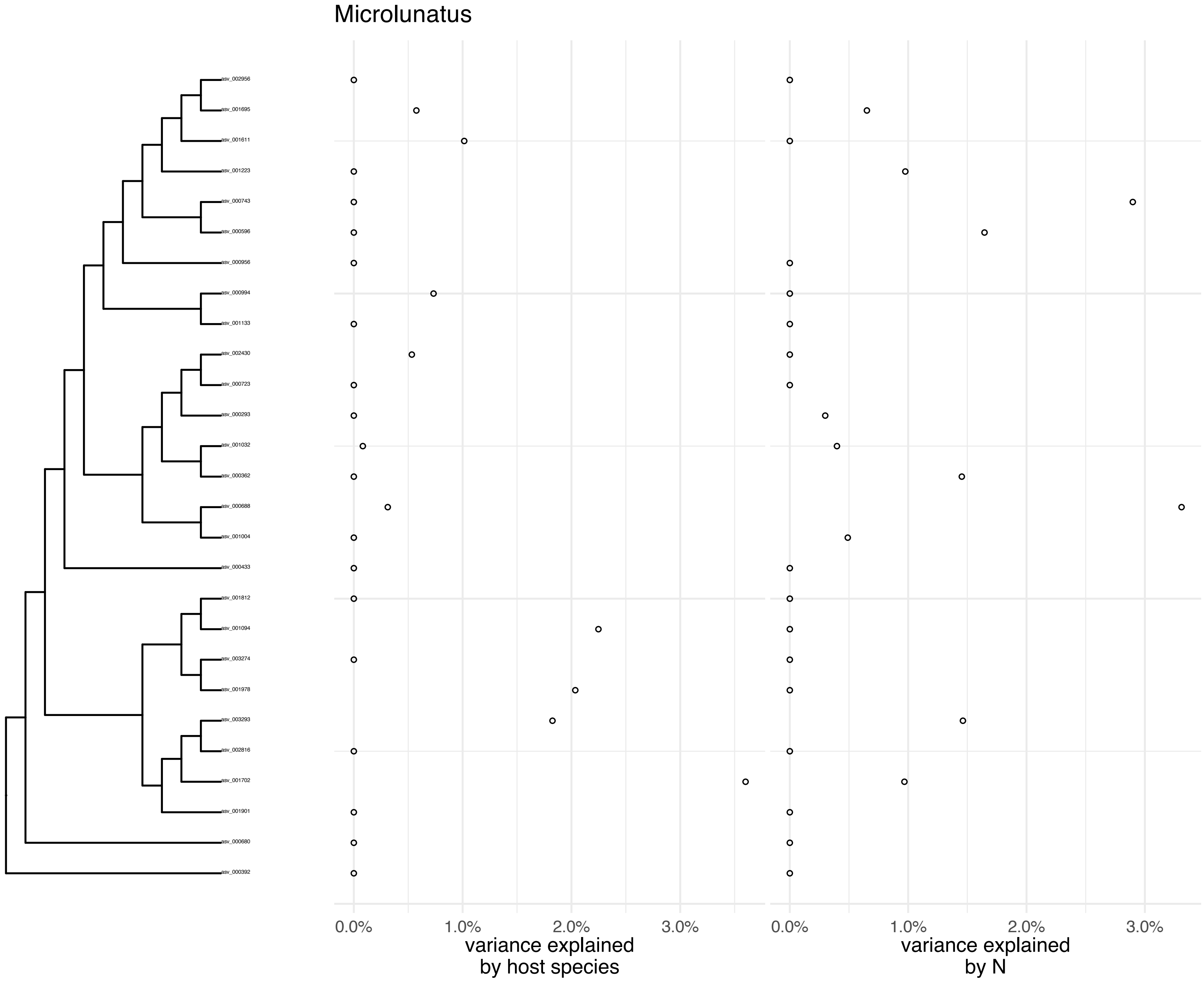

Microlunatus
asv_002956
asv_001695
asv_001611
asv_001223
asv_000743
asv_000596
asv_000956
asv_000994
asv_001133
asv_002430
asv_000723
asv_000293
asv_001032
asv_000362
asv_000688
asv_001004
asv_000433
asv_001812
asv_001094
asv_003274
asv_001978
asv_003293
asv_002816
asv_001702
asv_001901
asv_000680
asv_000392
0.0%
1.0%
2.0%
3.0%
variance explained
by host species
0.0%
1.0%
2.0%
3.0%
variance explained
by N

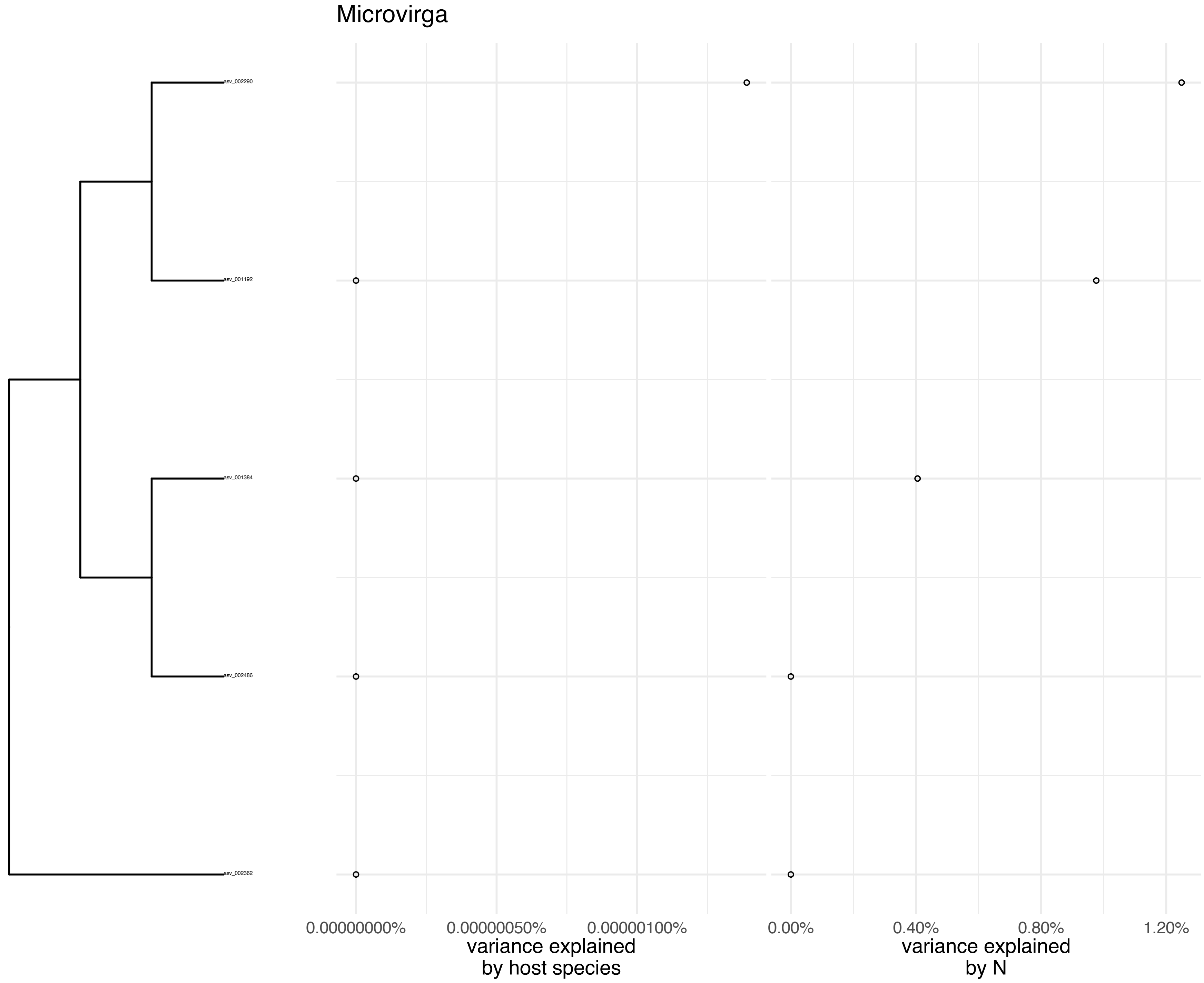

Microvirga
asv_002290
asv_001192
asv_001384
asv_002486
asv_002362
0.00000000%
0.00000050%
0.00000100%
variance explained
by host species
0.00%
0.40%
0.80%
1.20%
variance explained
by N

mle1-7

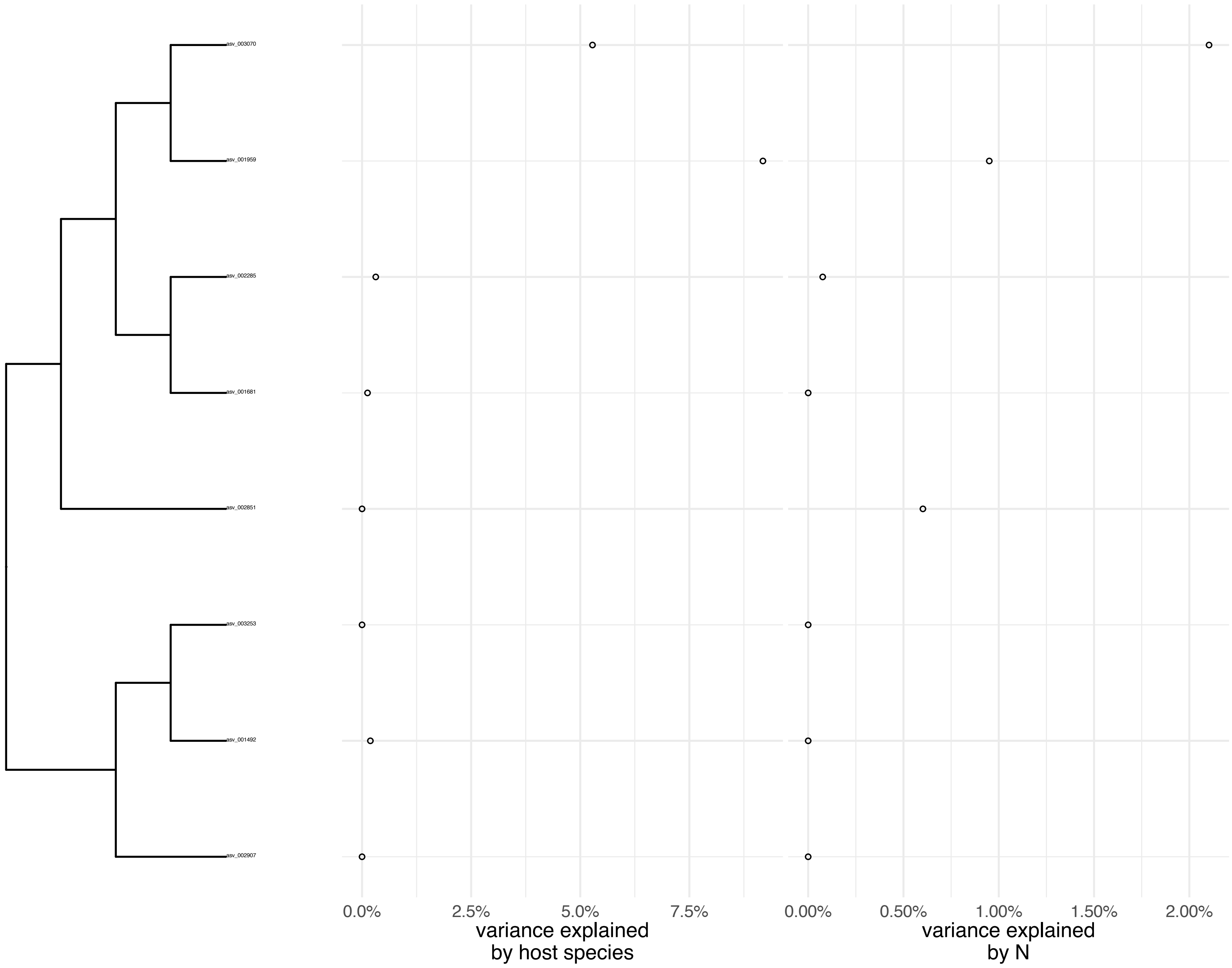

asv_003070
asv_001959
asv_002285
asv_001681
asv_002851
asv_003253
asv_001492
asv_002907
0.0%
2.5%
5.0%
7.5%
variance explained
by host species
0.00%
0.50%
1.00%
1.50%
2.00%
variance explained
by N

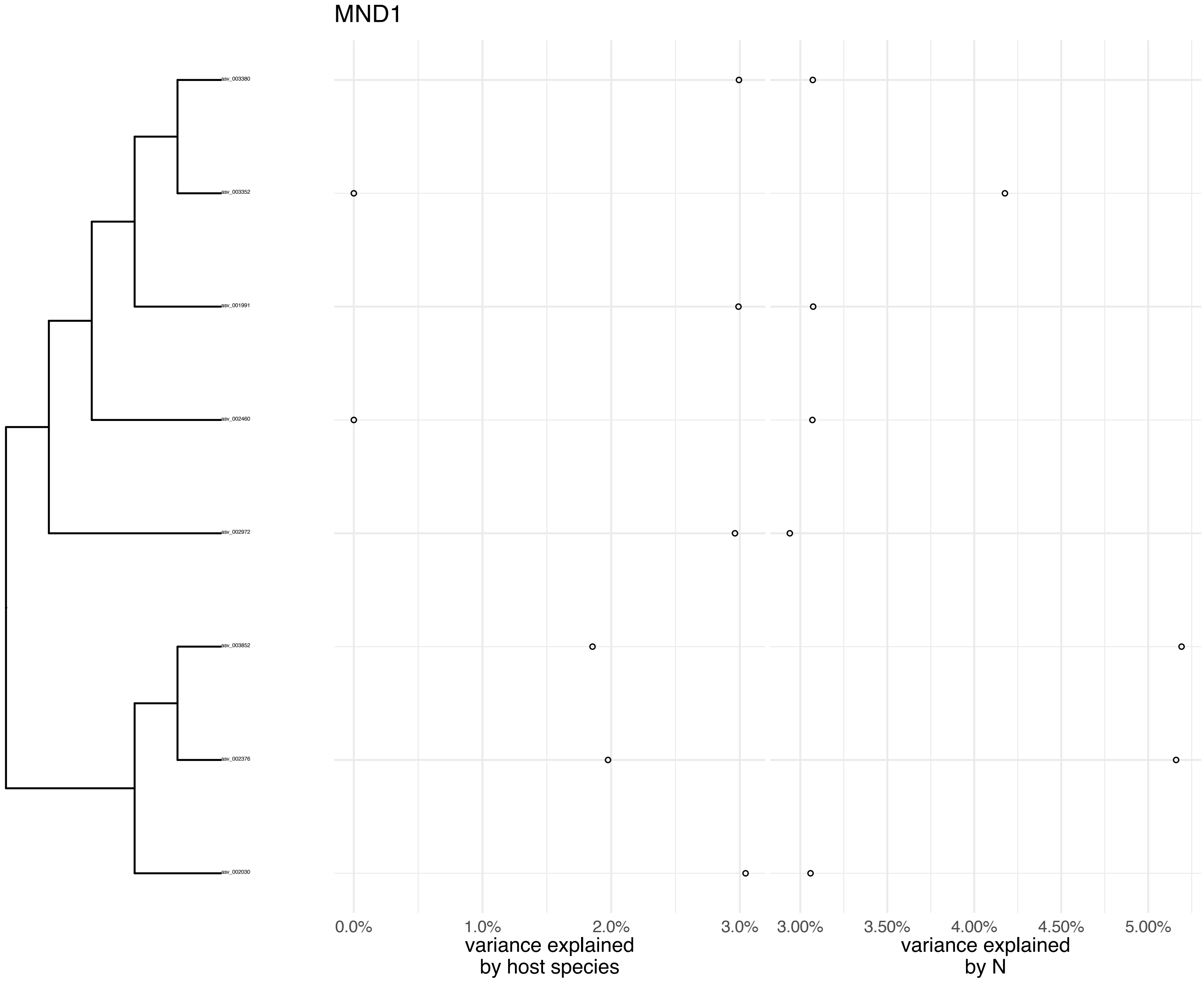

MND1
asv_003380
asv_003352
asv_001991
asv_002460
asv_002972
asv_003852
asv_002376
asv_002030
0.0%
1.0%
2.0%
3.0%
variance explained by host species
3.00%
3.50%
4.00%
4.50%
5.00%
variance explained by N

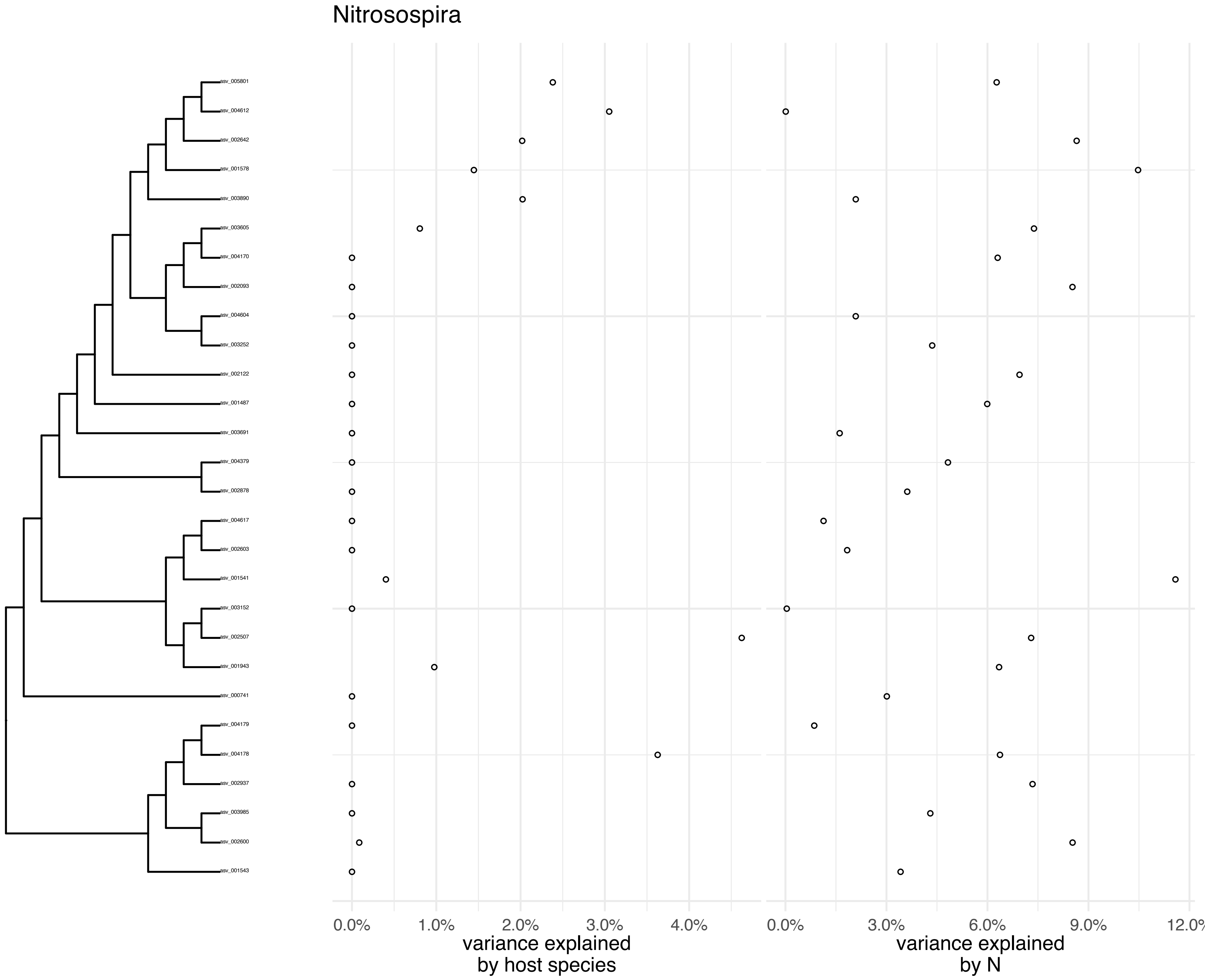
Nitrosospira
asv_005801
asv_004612
asv_002642
asv_001578
asv_003890
asv_003605
asv_004170
asv_002093
asv_004604
asv_003252
asv_002122
asv_001487
asv_003691
asv_004379
asv_002878
asv_004617
asv_002603
asv_001541
asv_003152
asv_002507
asv_001943
asv_000741
asv_004179
asv_004178
asv_002937
asv_003985
asv_002600
asv_001543
0.0%
1.0%
2.0%
3.0%
4.0%
variance explained by host species
0.0%
3.0%
6.0%
9.0%
12.0%
variance explained by N

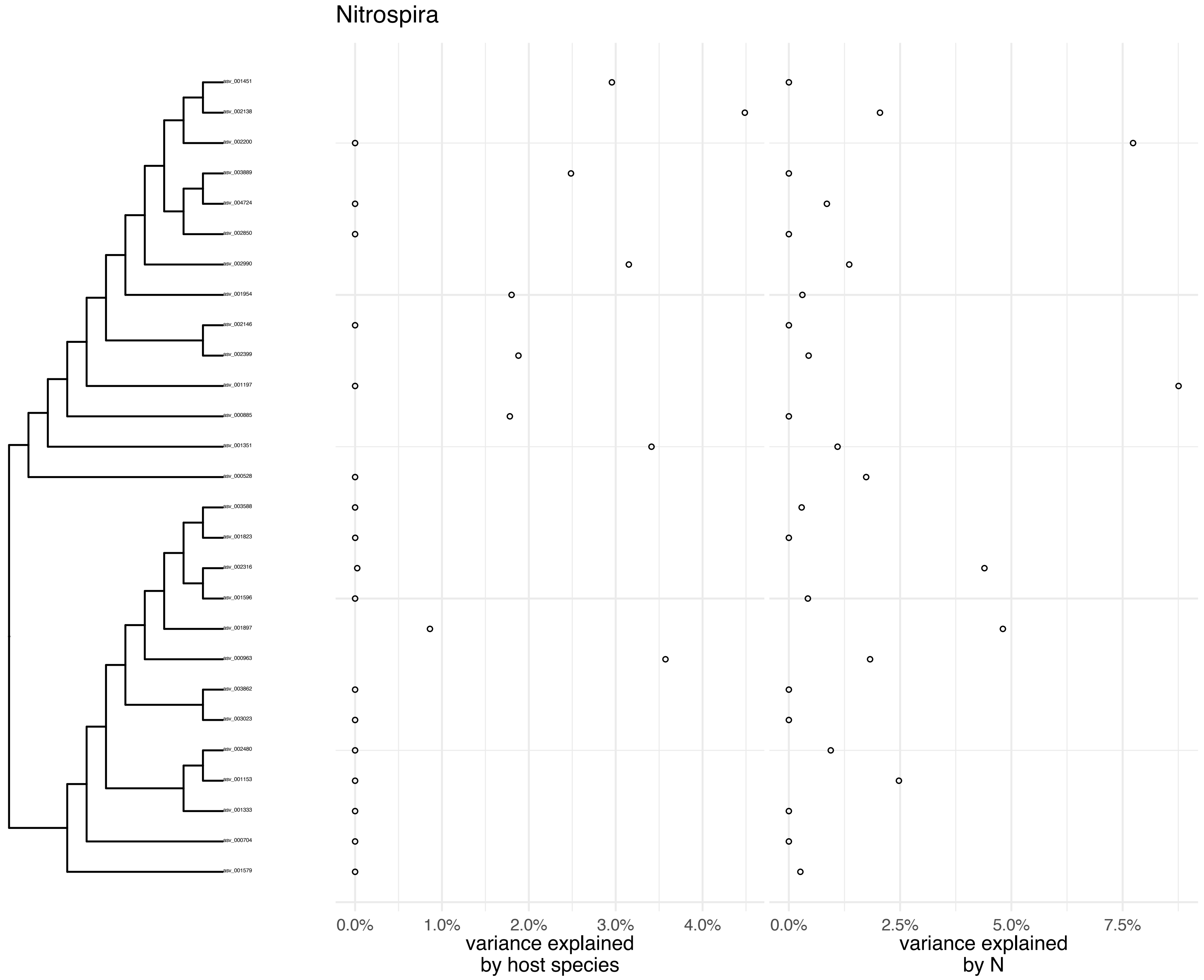

Nitrospira
asv_001451
asv_002138
asv_002200
asv_003889
asv_004724
asv_002850
asv_002990
asv_001954
asv_002146
asv_002399
asv_001197
asv_000885
asv_001351
asv_000528
asv_003588
asv_001823
asv_002316
asv_001596
asv_001897
asv_000963
asv_003862
asv_003023
asv_002480
asv_001153
asv_001333
asv_000704
asv_001579
0.0%
1.0%
2.0%
3.0%
4.0%
variance explained by host species
0.0%
2.5%
5.0%
7.5%
variance explained by N

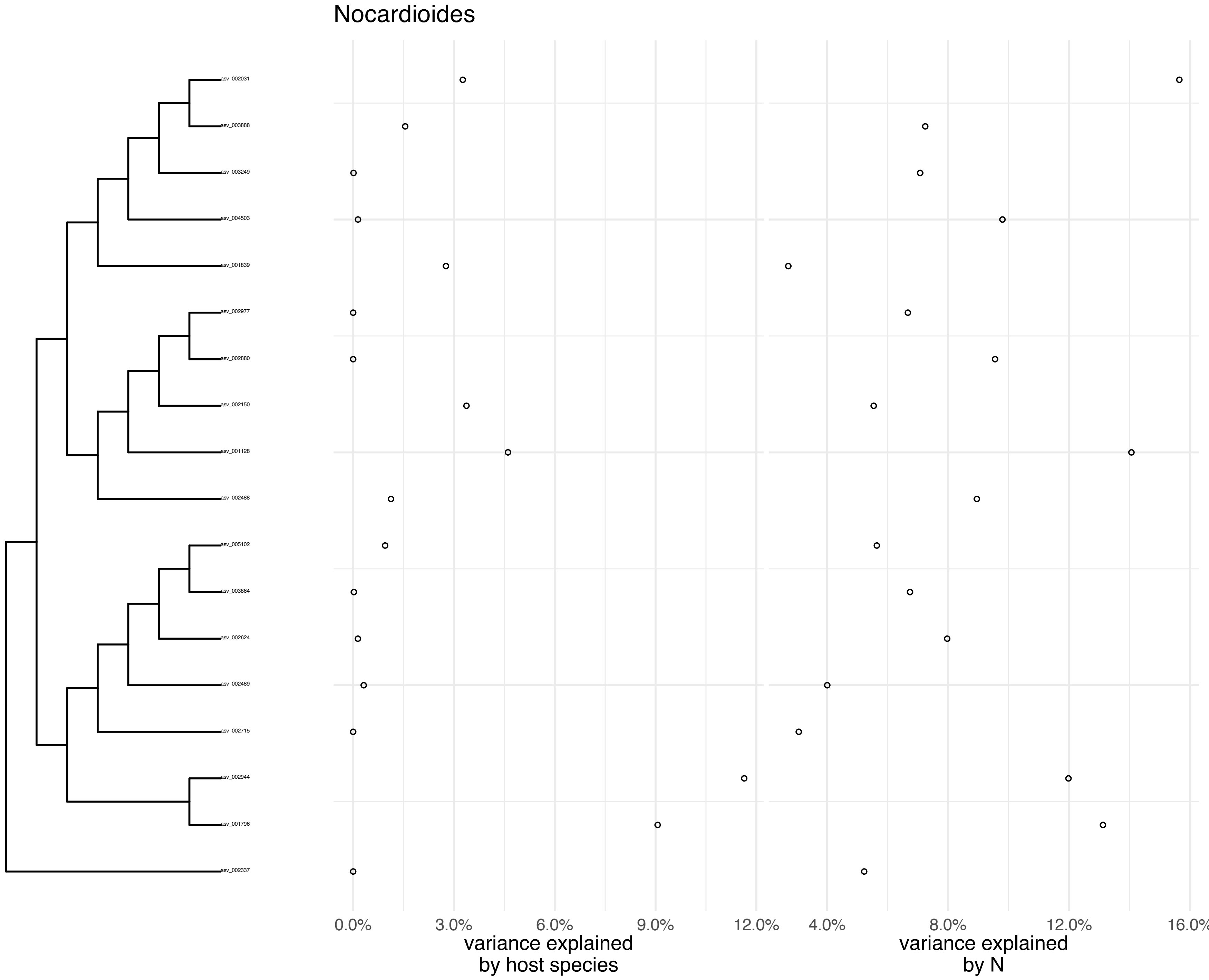

Nocardioides
asv_002031
asv_003888
asv_003249
asv_004503
asv_001839
asv_002977
asv_002880
asv_002150
asv_001128
asv_002488
asv_005102
asv_003864
asv_002624
asv_002489
asv_002715
asv_002944
asv_001796
asv_002337
0.0%
3.0%
6.0%
9.0%
12.0%
variance explained
by host species
4.0%
8.0%
12.0%
16.0%
variance explained
by N

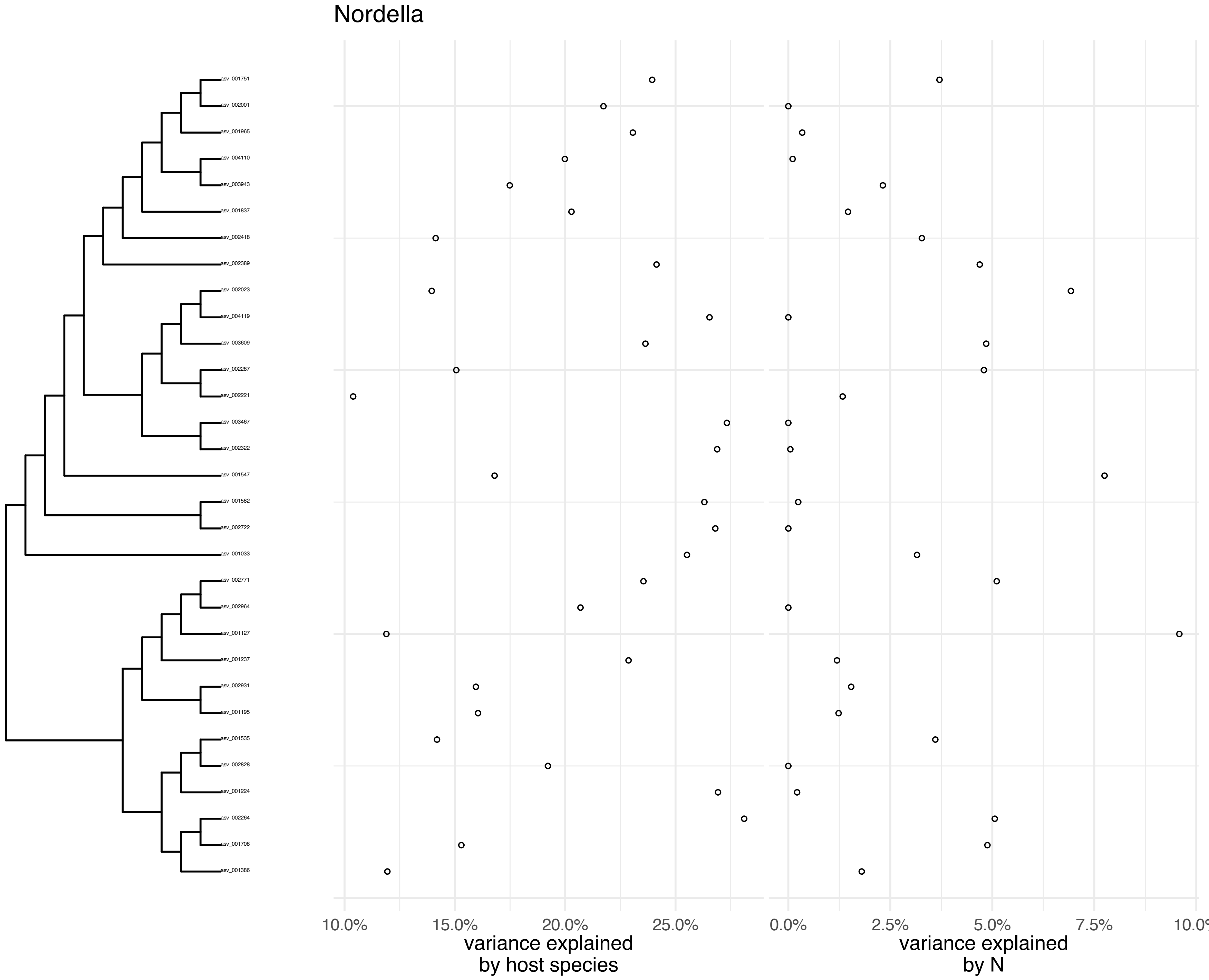
Nordella
asv_001751
asv_002001
asv_001965
asv_004110
asv_003943
asv_001837
asv_002418
asv_002389
asv_002023
asv_004119
asv_003609
asv_002287
asv_002221
asv_003467
asv_002322
asv_001547
asv_001582
asv_002722
asv_001033
asv_002771
asv_002964
asv_001127
asv_001237
asv_002931
asv_001195
asv_001535
asv_002828
asv_001224
asv_002264
asv_001708
asv_001386
10.0%
15.0%
20.0%
25.0%
variance explained
by host species
0.0%
2.5%
5.0%
7.5%
10.0%
variance explained
by N

Novosphingobium

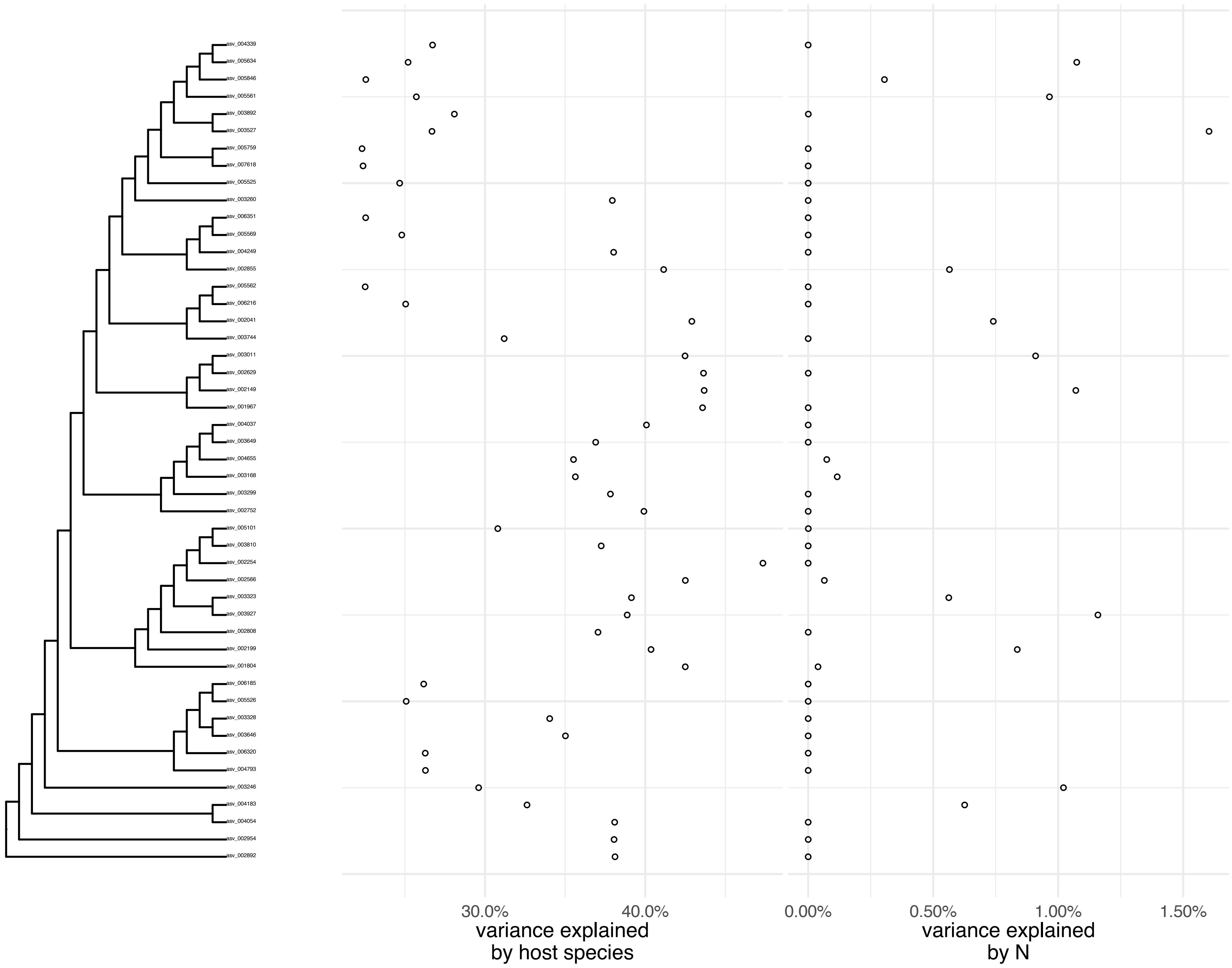

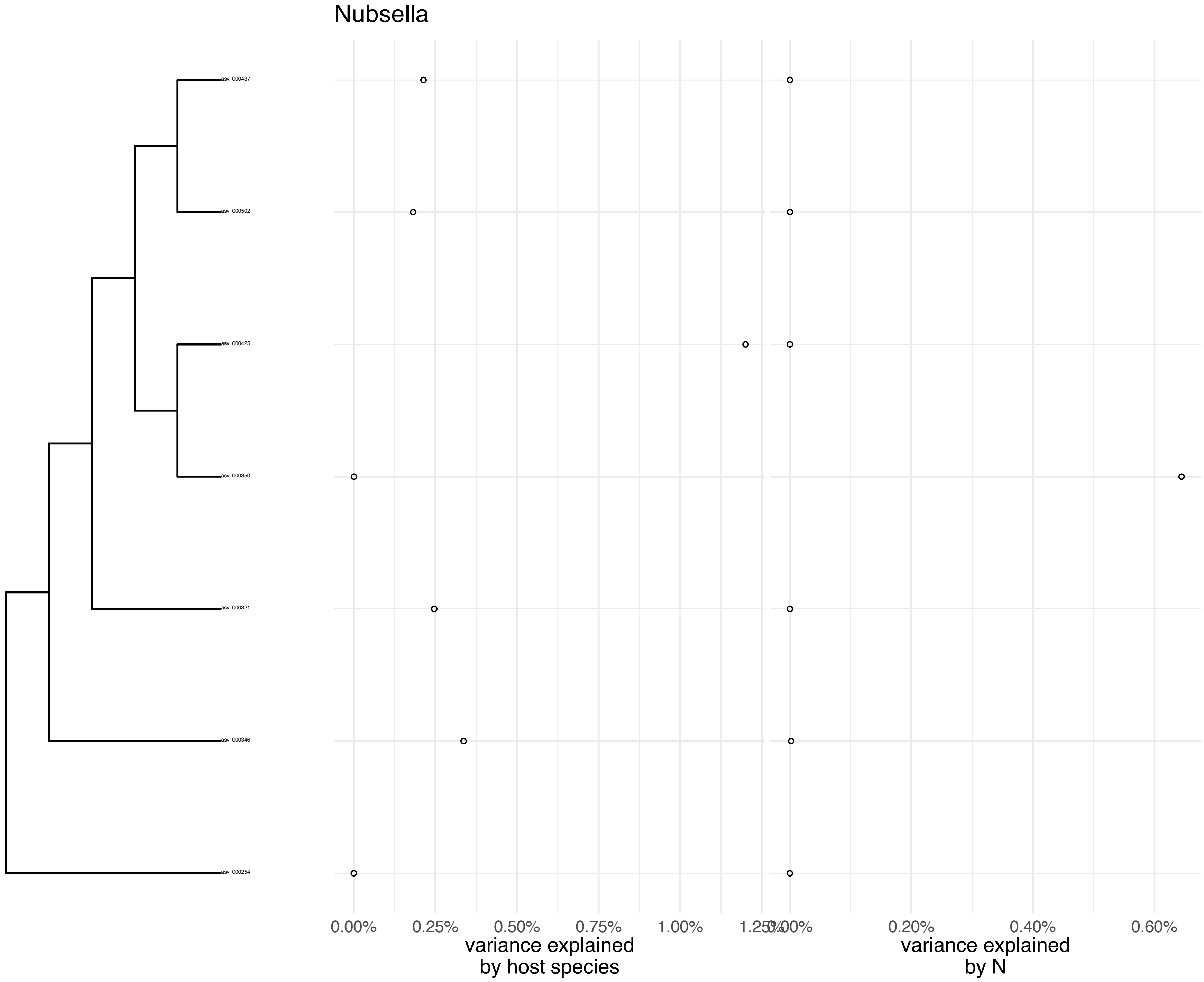

Nubsella
asv_000437
asv_000502
asv_000425
asv_000350
asv_000321
asv_000346
asv_000254
0.00%
0.25%
0.50%
0.75%
1.00%
1.25%
variance explained by host species
0.00%
0.20%
0.40%
0.60%
variance explained by N

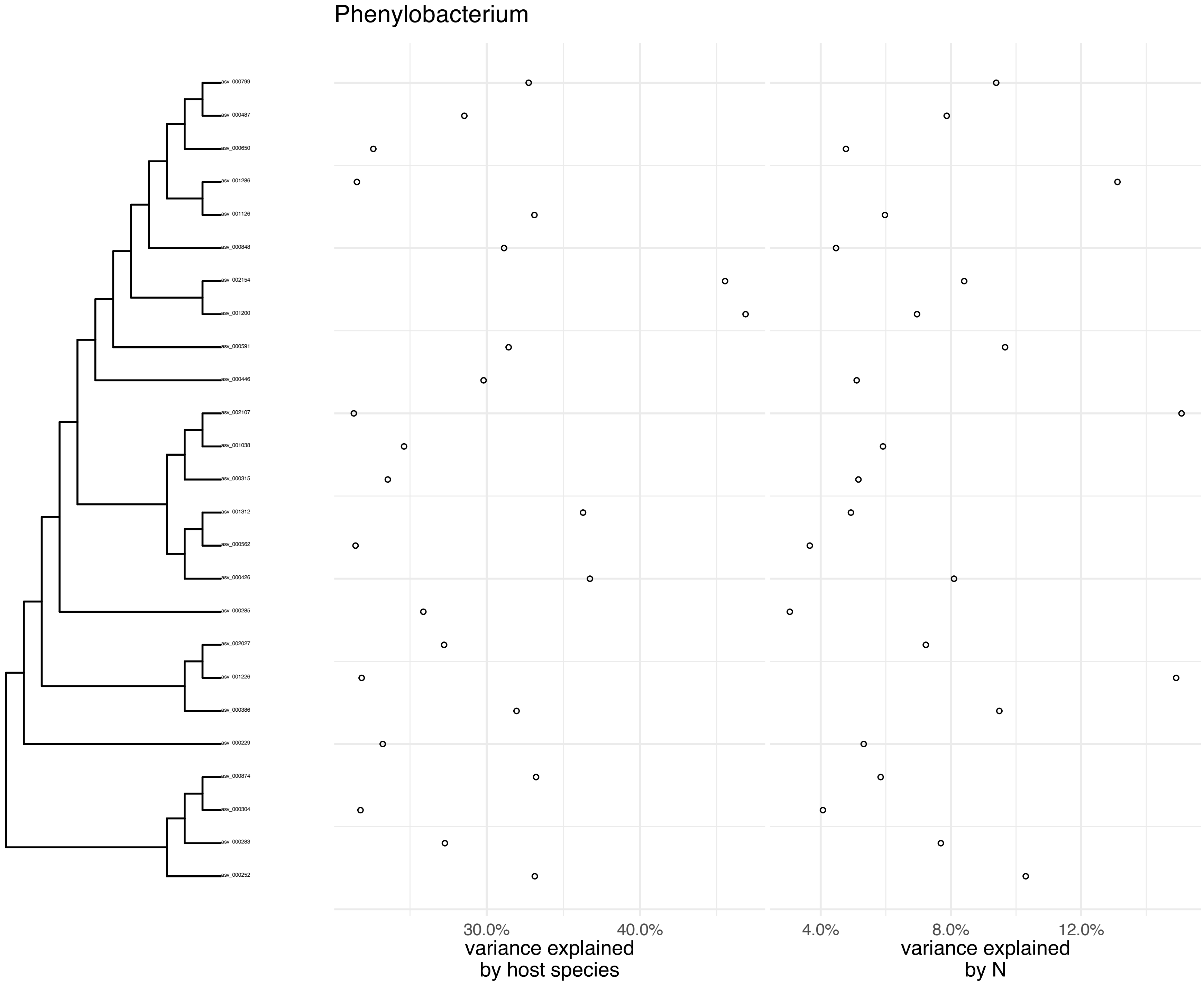
Phenylobacterium
asv_000799
asv_000487
asv_000650
asv_001286
asv_001126
asv_000848
asv_002154
asv_001200
asv_000591
asv_000446
asv_002107
asv_001038
asv_000315
asv_001312
asv_000562
asv_000426
asv_000285
asv_002027
asv_001226
asv_000386
asv_000229
asv_000874
asv_000304
asv_000283
asv_000252
30.0%
40.0%
variance explained
by host species
4.0%
8.0%
12.0%
variance explained
by N

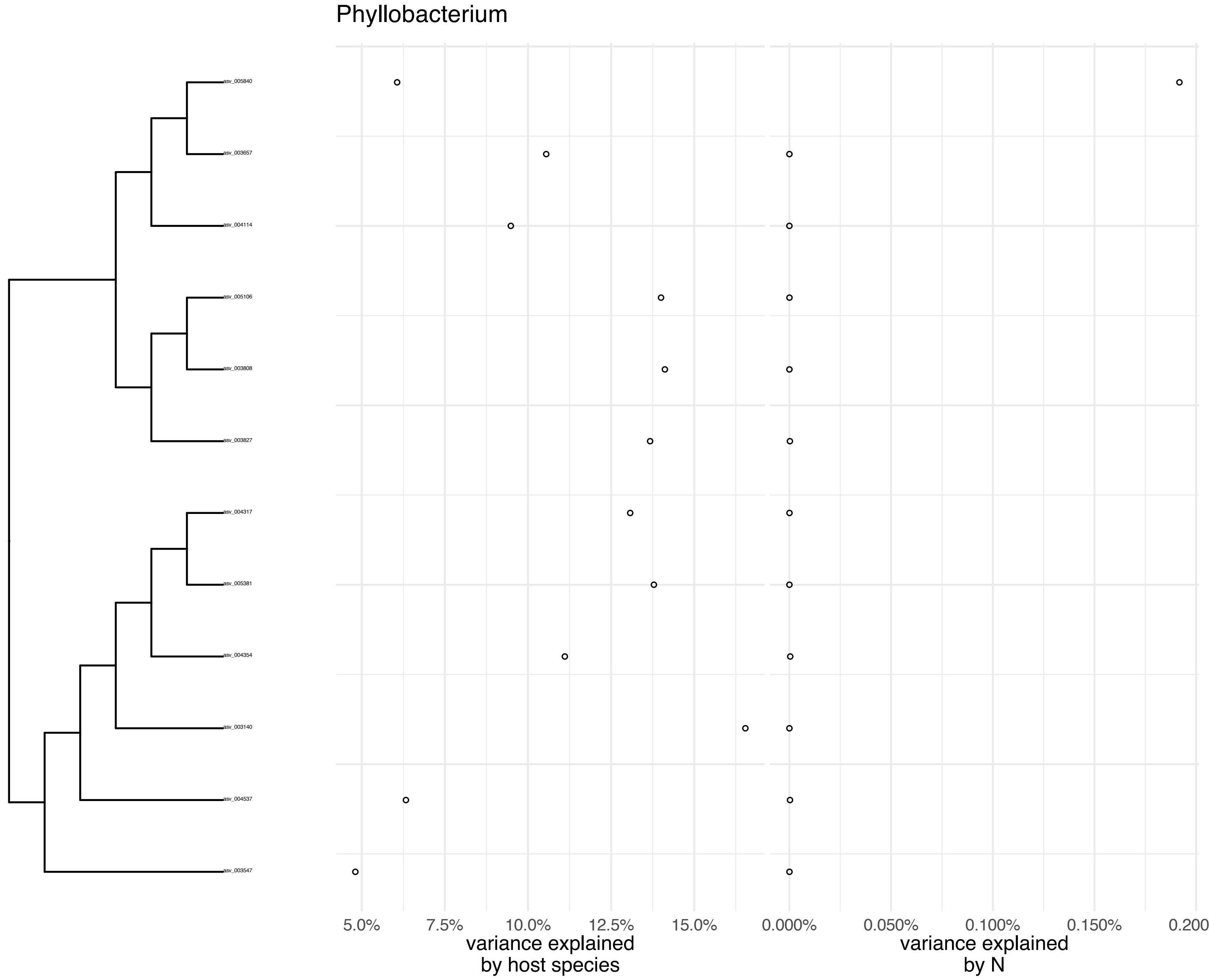
Phyllobacterium
asv_005840
asv_003657
asv_004114
asv_005106
asv_003808
asv_003827
asv_004317
asv_005381
asv_004354
asv_003140
asv_004537
asv_003547
5.0%
7.5%
10.0%
12.5%
15.0%
0.000%
0.050%
0.100%
0.150%
0.200
variance explained
by host species
variance explained
by N

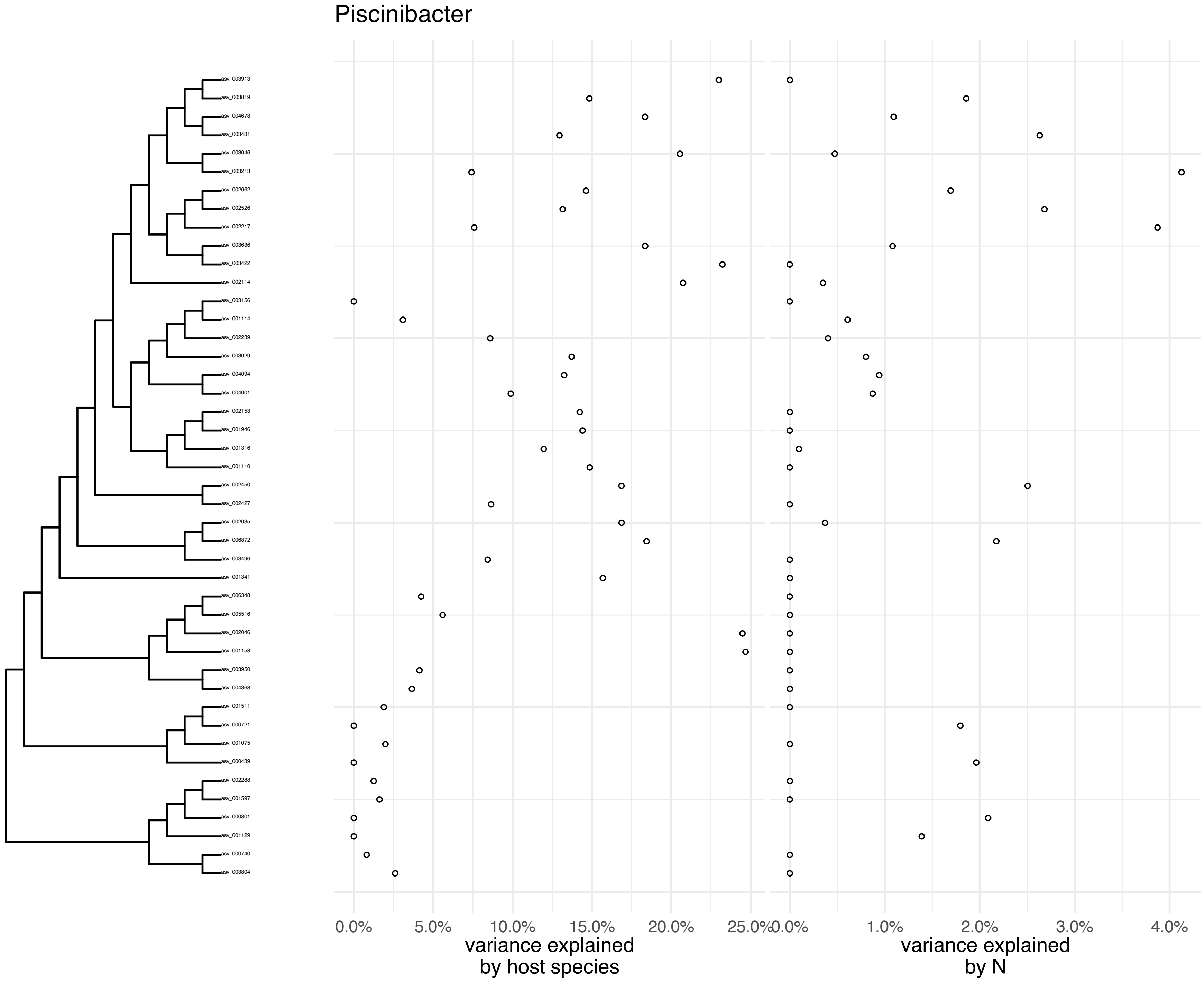
Piscinibacter
asv_003913
asv_003819
asv_004678
asv_003481
asv_003046
asv_003213
asv_002662
asv_002526
asv_002217
asv_003636
asv_003422
asv_002114
asv_003156
asv_001114
asv_002239
asv_003029
asv_004094
asv_004001
asv_002153
asv_001946
asv_001316
asv_001110
asv_002450
asv_002427
asv_002035
asv_006872
asv_003496
asv_001341
asv_006348
asv_005516
asv_002046
asv_001158
asv_003950
asv_004368
asv_001511
asv_000721
asv_001075
asv_000439
asv_002288
asv_001597
asv_000801
asv_001129
asv_000740
asv_003804
0.0%
5.0%
10.0%
15.0%
20.0%
25.0%
variance explained
by host species
0.0%
1.0%
2.0%
3.0%
4.0%
variance explained
by N

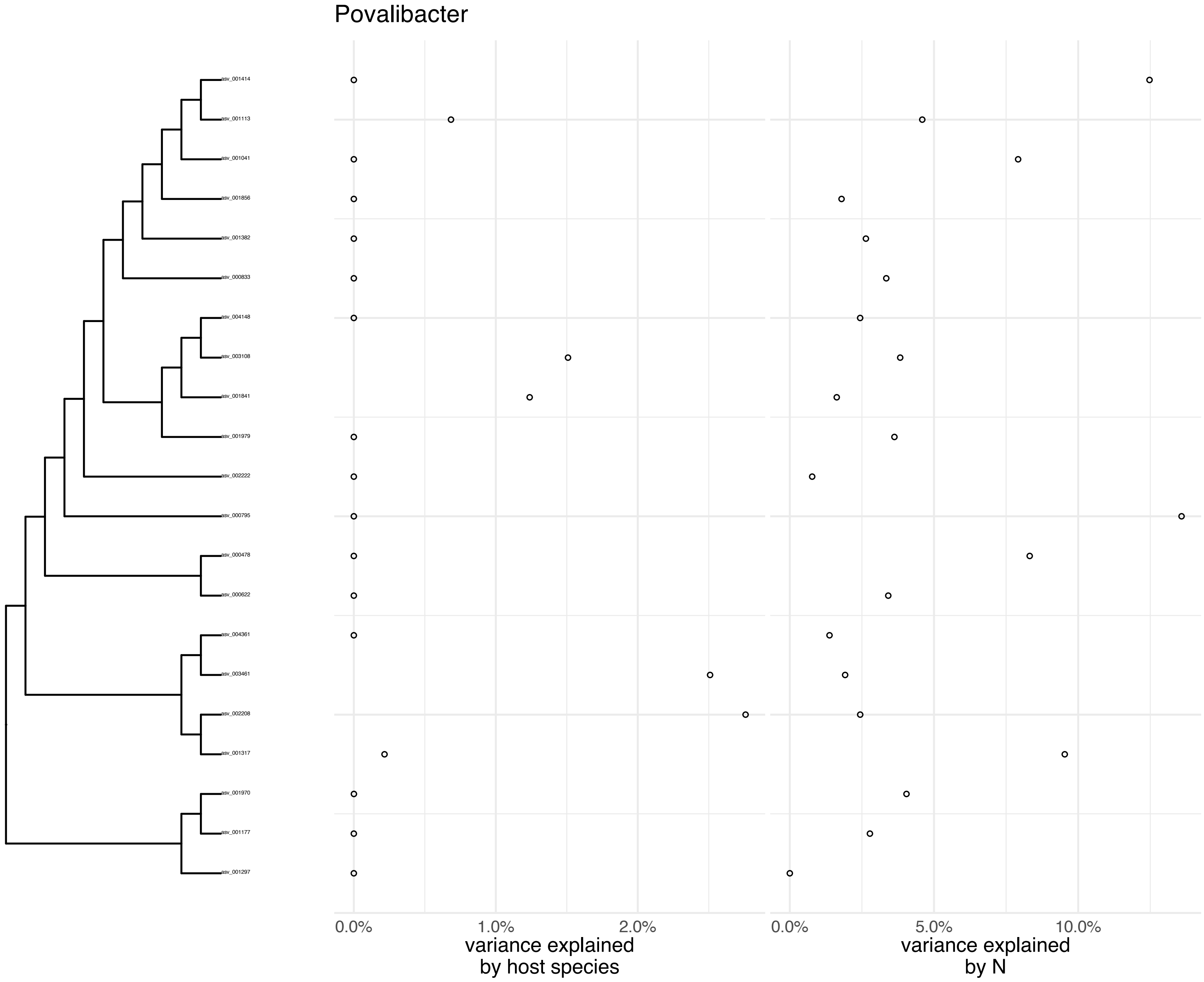
Povalibacter
asv_001414
asv_001113
asv_001041
asv_001856
asv_001382
asv_000833
asv_004148
asv_003108
asv_001841
asv_001979
asv_002222
asv_000795
asv_000478
asv_000622
asv_004361
asv_003461
asv_002208
asv_001317
asv_001970
asv_001177
asv_001297
0.0%
1.0%
2.0%
variance explained by host species
0.0%
5.0%
10.0%
variance explained by N

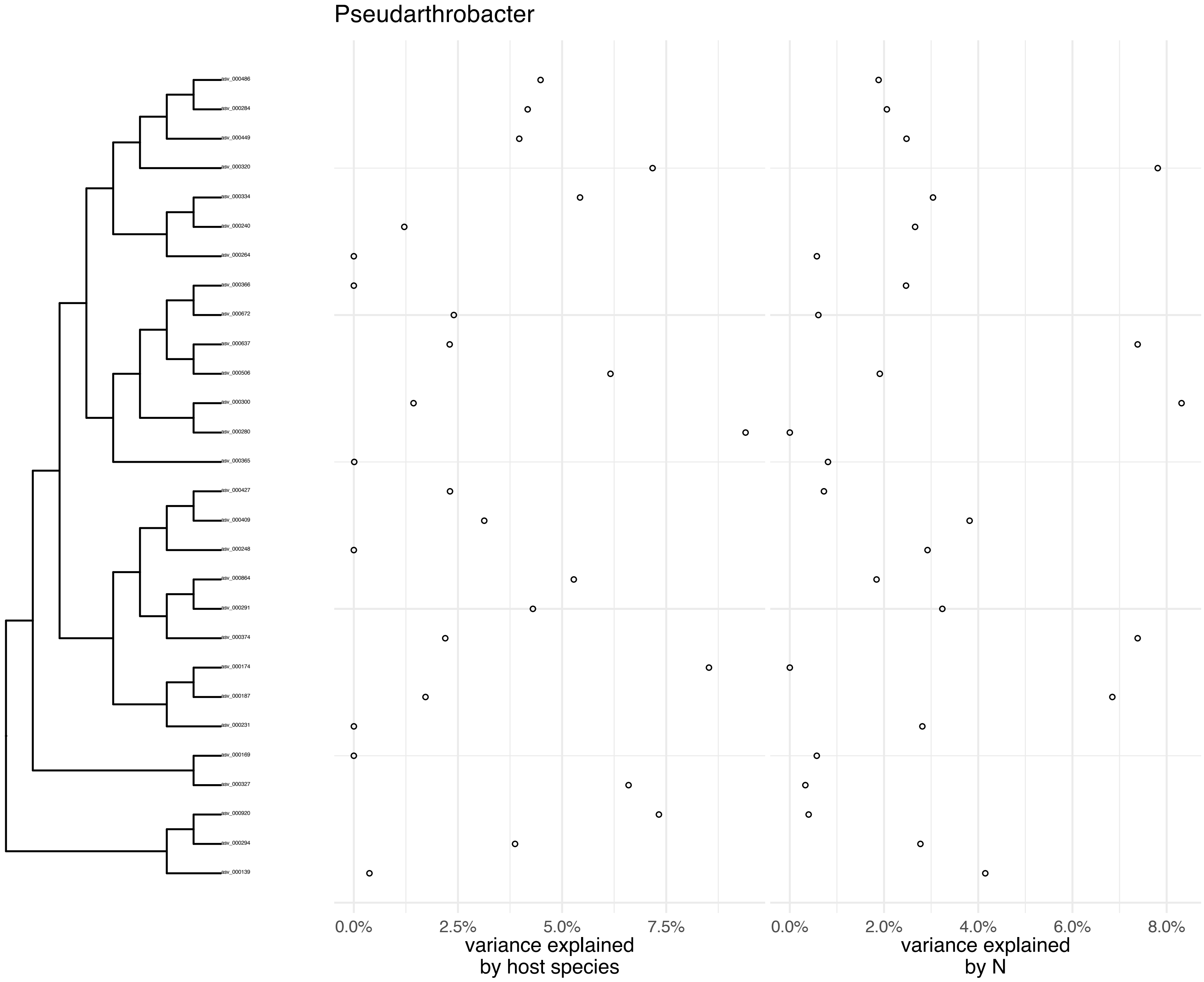
Pseudarthrobacter
asv_000486
asv_000284
asv_000449
asv_000320
asv_000334
asv_000240
asv_000264
asv_000366
asv_000672
asv_000637
asv_000506
asv_000300
asv_000280
asv_000365
asv_000427
asv_000409
asv_000248
asv_000864
asv_000291
asv_000374
asv_000174
asv_000187
asv_000231
asv_000169
asv_000327
asv_000920
asv_000294
asv_000139
0.0%
2.5%
5.0%
7.5%
variance explained by host species
0.0%
2.0%
4.0%
6.0%
8.0%
variance explained by N

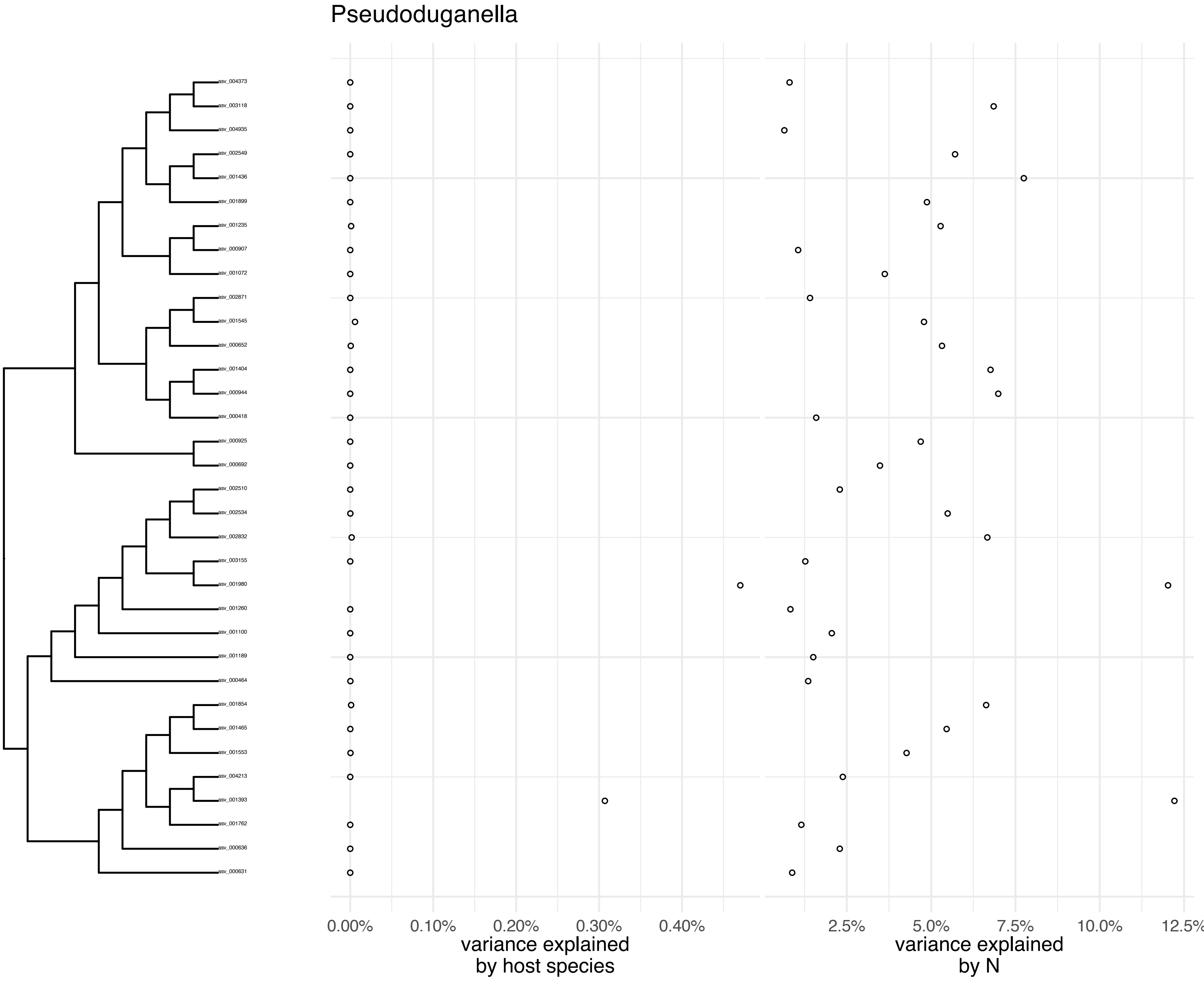
Pseudoduganella
asv_004373
asv_003118
asv_004935
asv_002549
asv_001436
asv_001899
asv_001235
asv_000907
asv_001072
asv_002871
asv_001545
asv_000652
asv_001404
asv_000944
asv_000418
asv_000925
asv_000692
asv_002510
asv_002534
asv_002832
asv_003155
asv_001980
asv_001260
asv_001100
asv_001189
asv_000464
asv_001854
asv_001465
asv_001553
asv_004213
asv_001393
asv_001762
asv_000636
asv_000631
0.00%
0.10%
0.20%
0.30%
0.40%
variance explained by host species
2.5%
5.0%
7.5%
10.0%
12.5%
variance explained by N

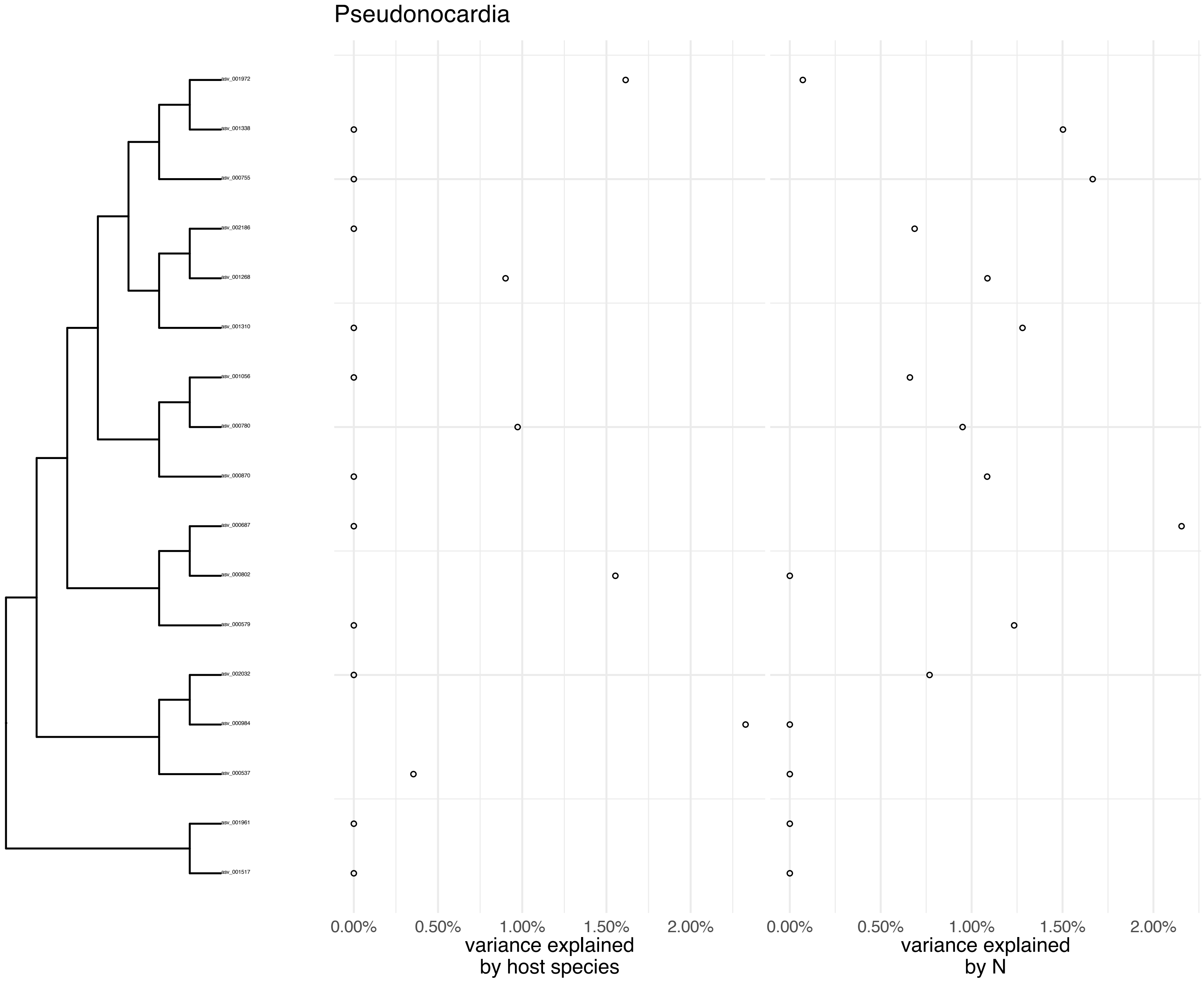
Pseudonocardia
asv_001972
asv_001338
asv_000755
asv_002186
asv_001268
asv_001310
asv_001056
asv_000780
asv_000870
asv_000687
asv_000802
asv_000579
asv_002032
asv_000984
asv_000537
asv_001961
asv_001517
0.00%
0.50%
1.00%
1.50%
2.00%
variance explained by host species
0.00%
0.50%
1.00%
1.50%
2.00%
variance explained by N

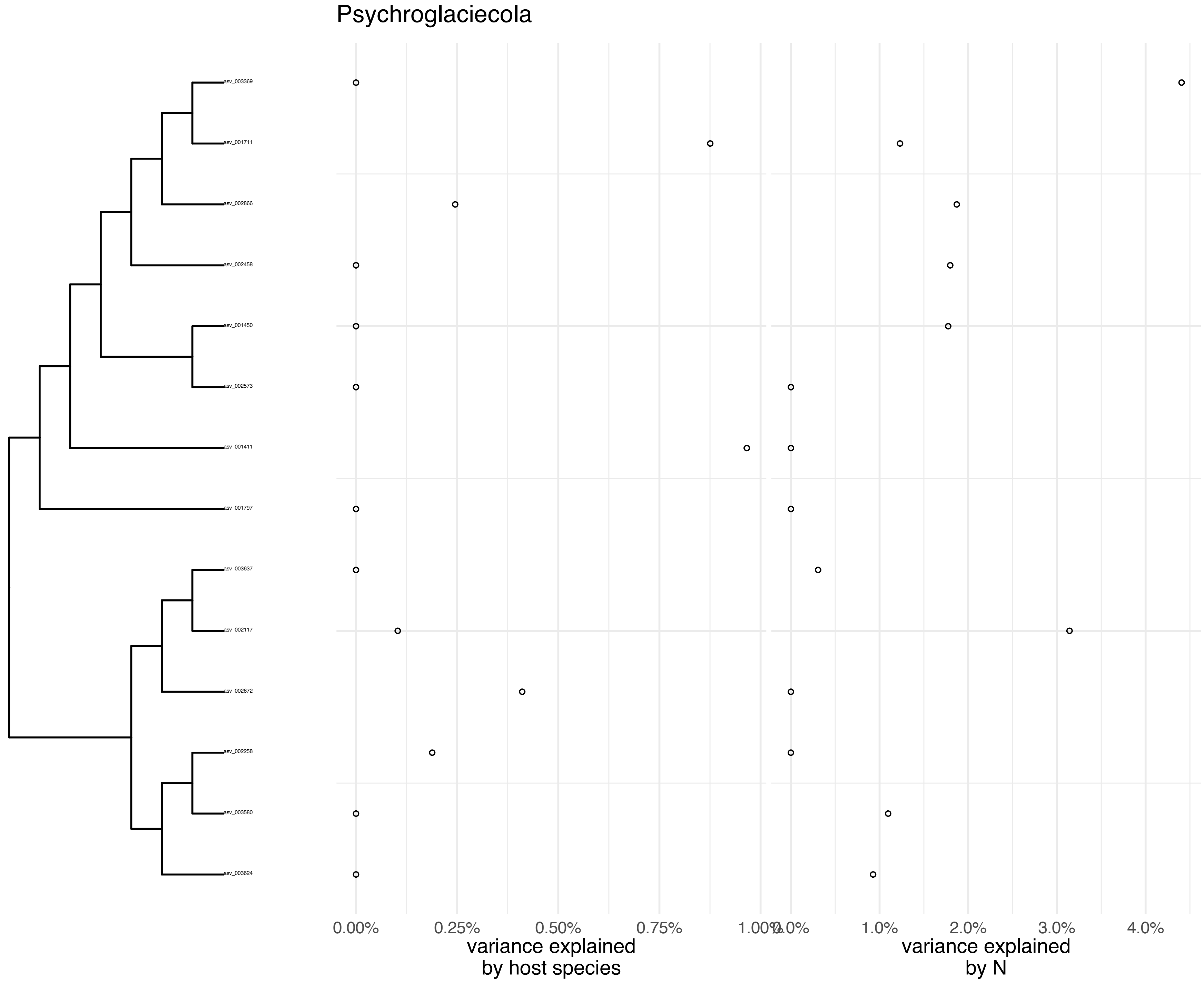
Psychroglaciecola
asv_003369
asv_001711
asv_002866
asv_002458
asv_001450
asv_002573
asv_001411
asv_001797
asv_003637
asv_002117
asv_002672
asv_002258
asv_003580
asv_003624
0.00%
0.25%
0.50%
0.75%
1.00%
0.0%
1.0%
2.0%
3.0%
4.0%
variance explained
by host species
variance explained
by N

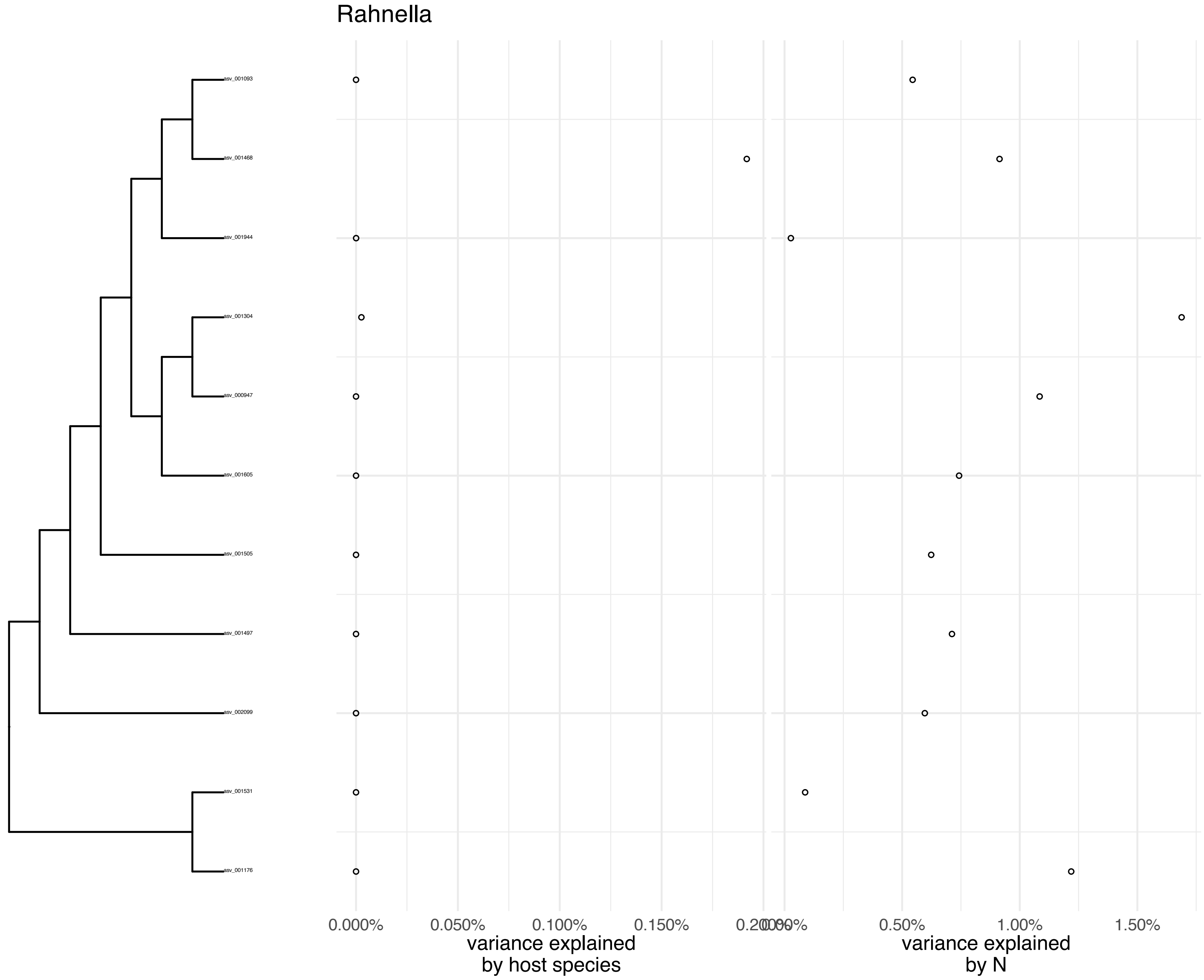
Rahnella
asv_001093
asv_001468
asv_001944
asv_001304
asv_000947
asv_001605
asv_001505
asv_001497
asv_002099
asv_001531
asv_001176
0.000%
0.050%
0.100%
0.150%
0.200%
variance explained
by host species
0.00%
0.50%
1.00%
1.50%
variance explained
by N

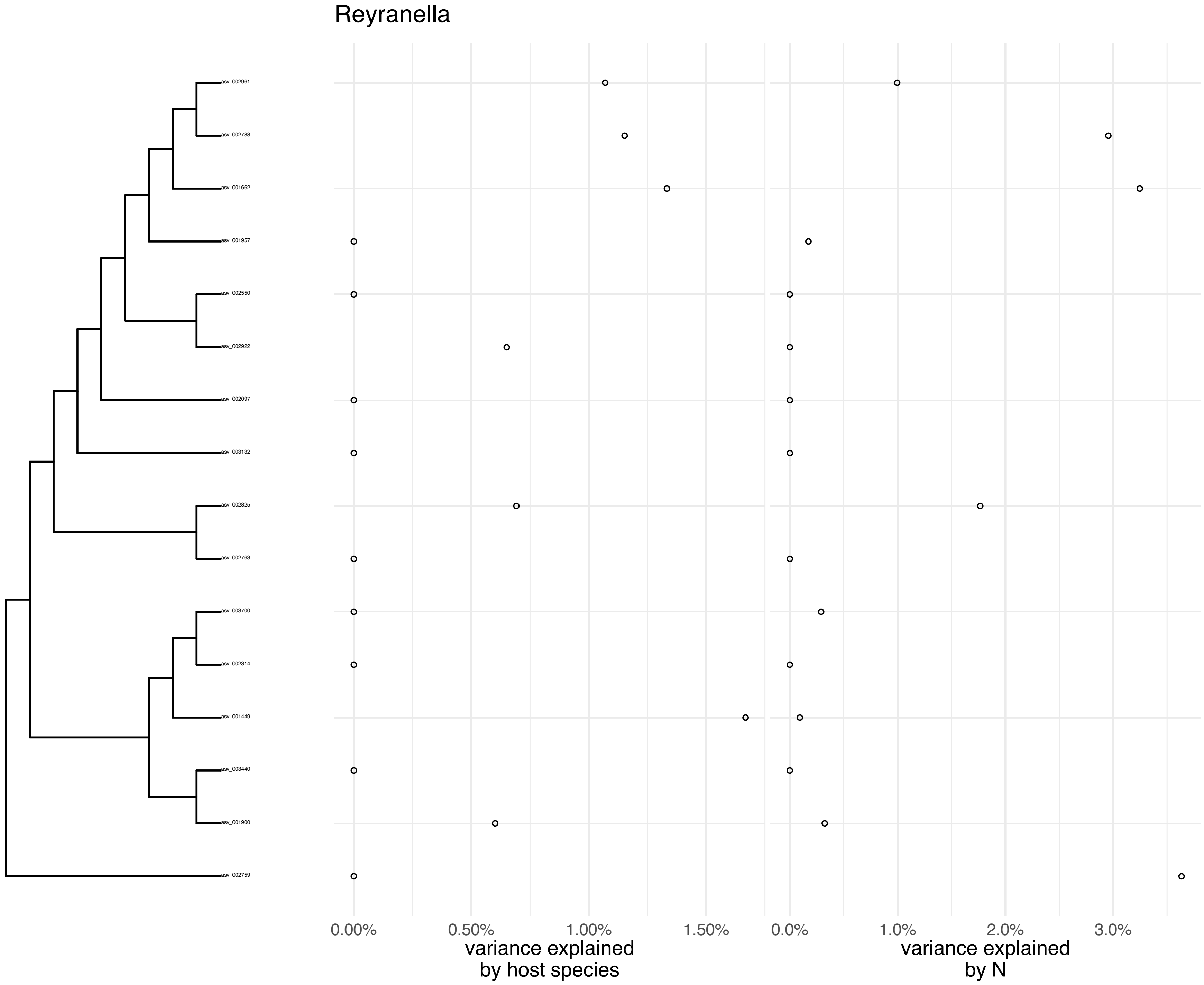

Reyranella
asv_002961
asv_002788
asv_001662
asv_001957
asv_002550
asv_002922
asv_002097
asv_003132
asv_002825
asv_002763
asv_003700
asv_002314
asv_001449
asv_003440
asv_001900
asv_002759
0.00%
0.50%
1.00%
1.50%
variance explained by host species
0.0%
1.0%
2.0%
3.0%
variance explained by N

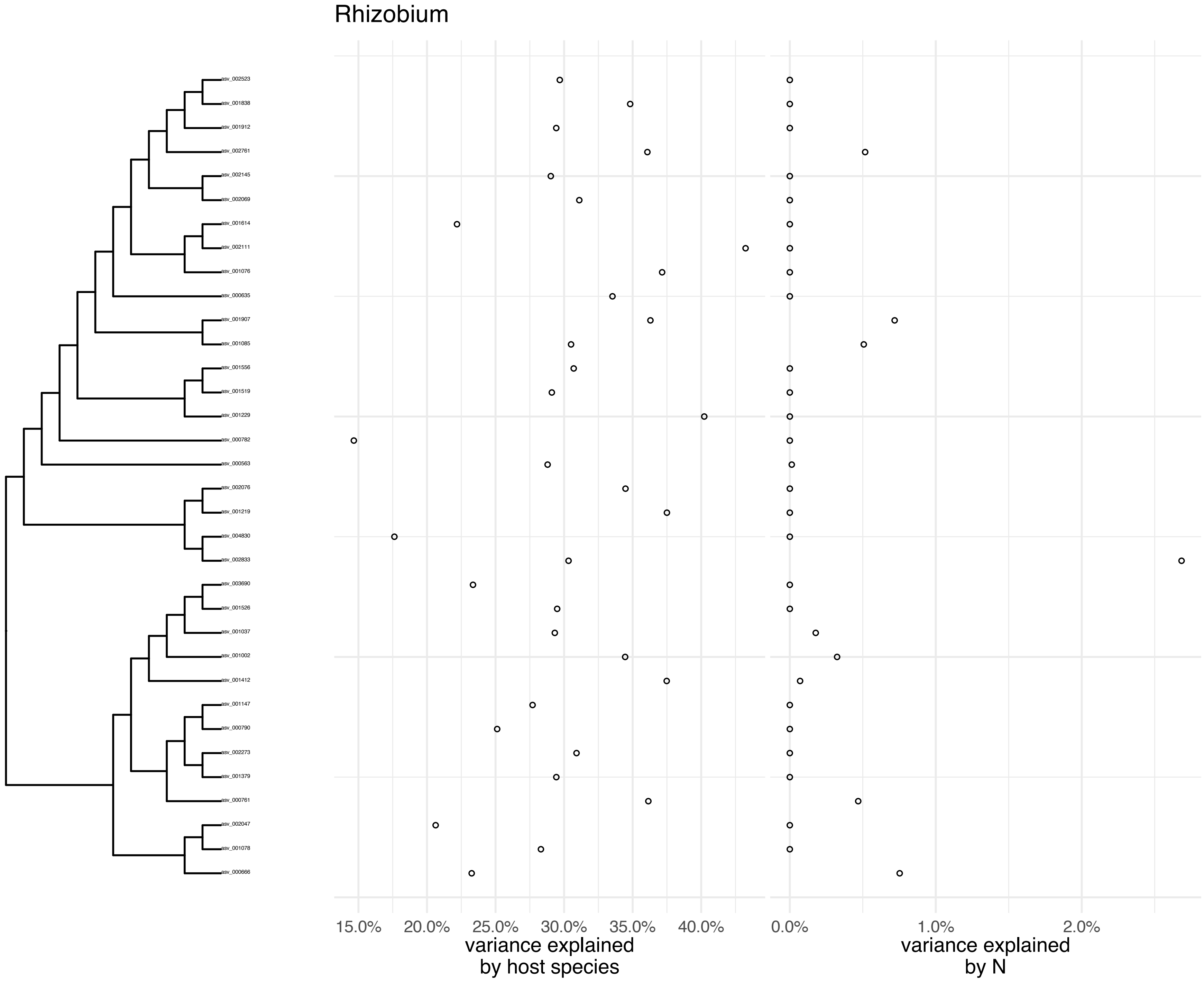
Rhizobium
asv_002523
asv_001838
asv_001912
asv_002761
asv_002145
asv_002069
asv_001614
asv_002111
asv_001076
asv_000635
asv_001907
asv_001085
asv_001556
asv_001519
asv_001229
asv_000782
asv_000563
asv_002076
asv_001219
asv_004830
asv_002833
asv_003690
asv_001526
asv_001037
asv_001002
asv_001412
asv_001147
asv_000790
asv_002273
asv_001379
asv_000761
asv_002047
asv_001078
asv_000666
15.0%
20.0%
25.0%
30.0%
35.0%
40.0%
variance explained
by host species
0.0%
1.0%
2.0%
variance explained
by N

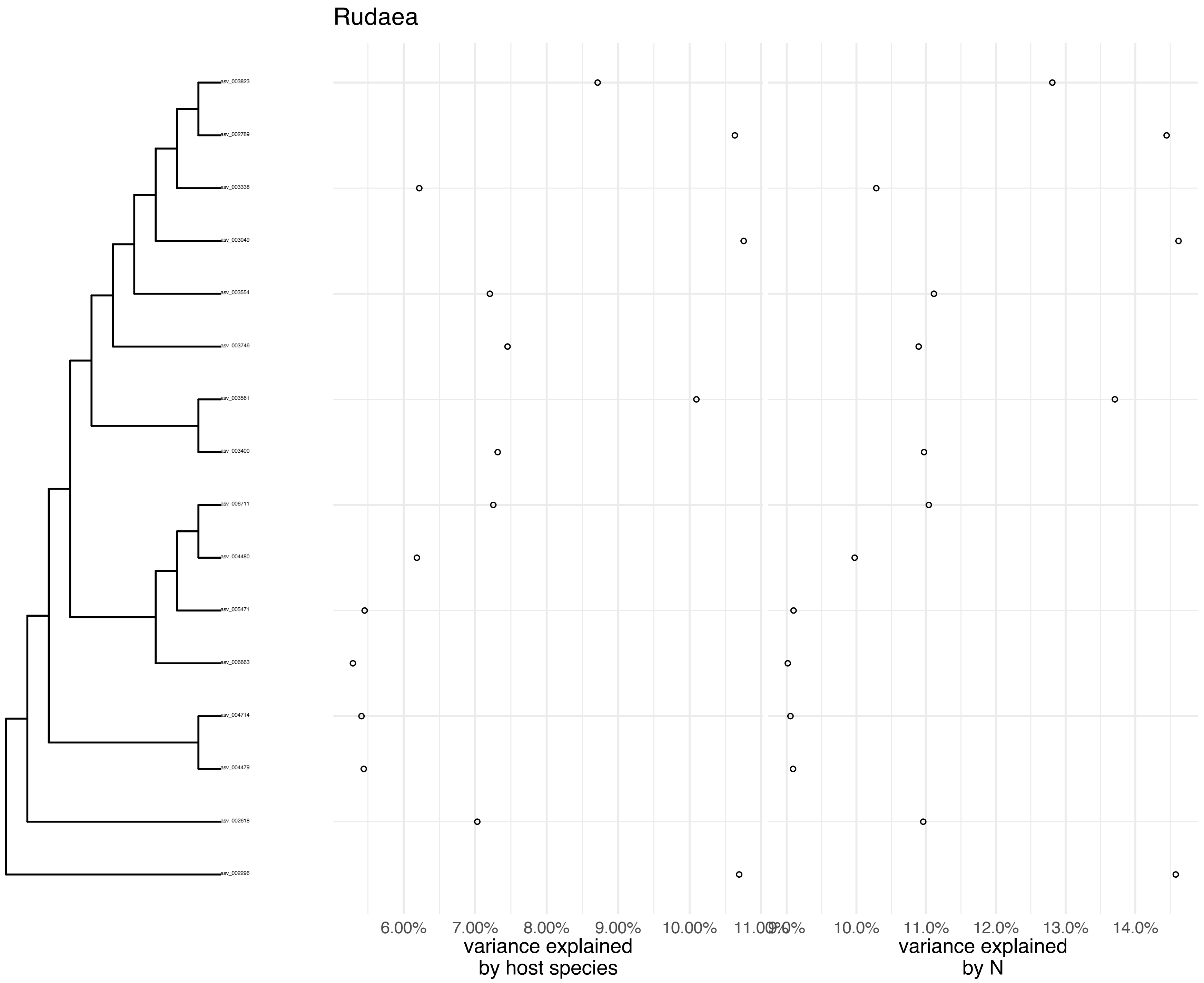

Rudaea
asv_003823
asv_002789
asv_003338
asv_003049
asv_003554
asv_003746
asv_003561
asv_003400
asv_006711
asv_004480
asv_005471
asv_006663
asv_004714
asv_004479
asv_002618
asv_002296
6.00%
7.00%
8.00%
9.00%
10.00%
11.00%
9.0%
10.0%
11.0%
12.0%
13.0%
14.0%
variance explained
by host species
variance explained
by N

Skermanella

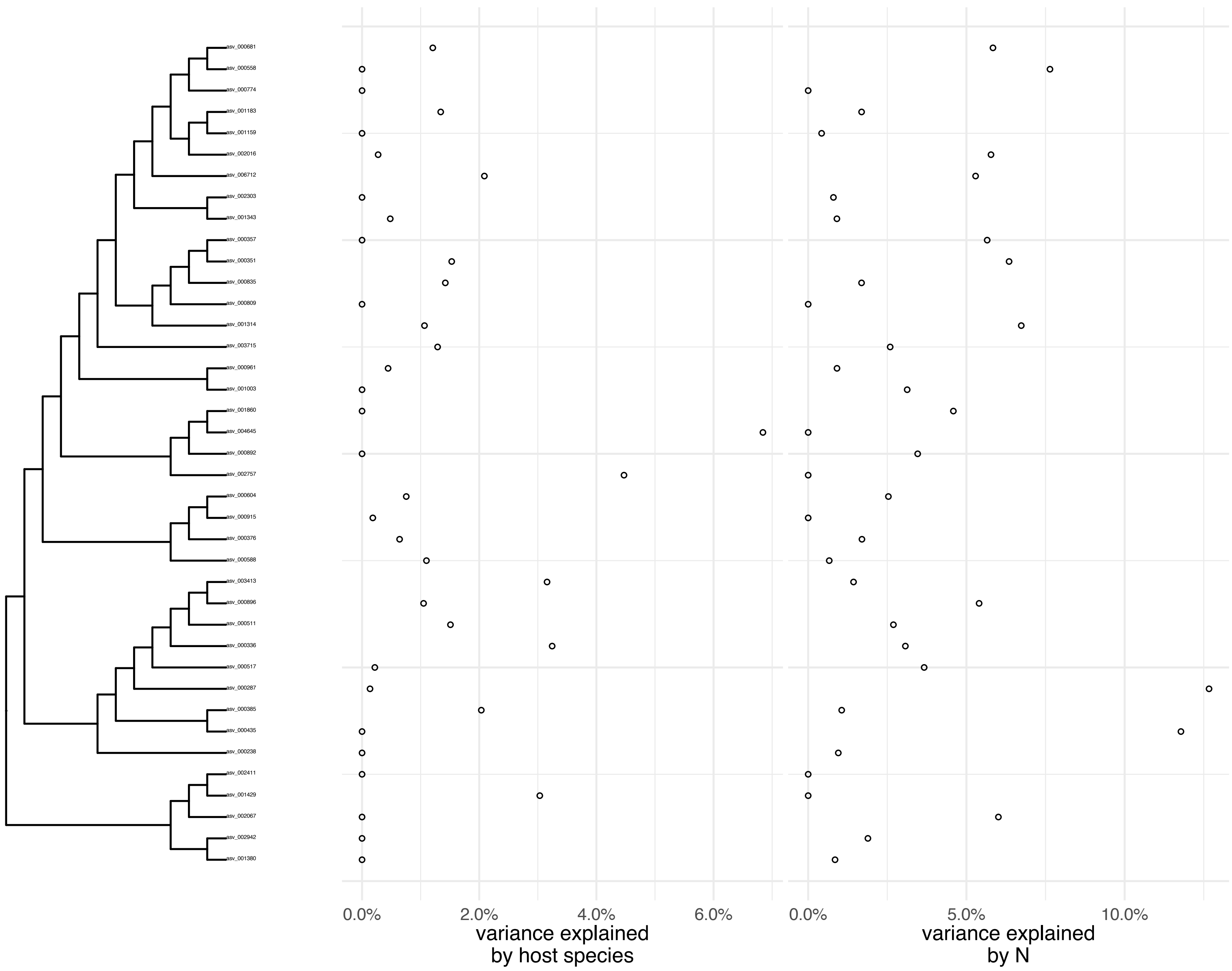

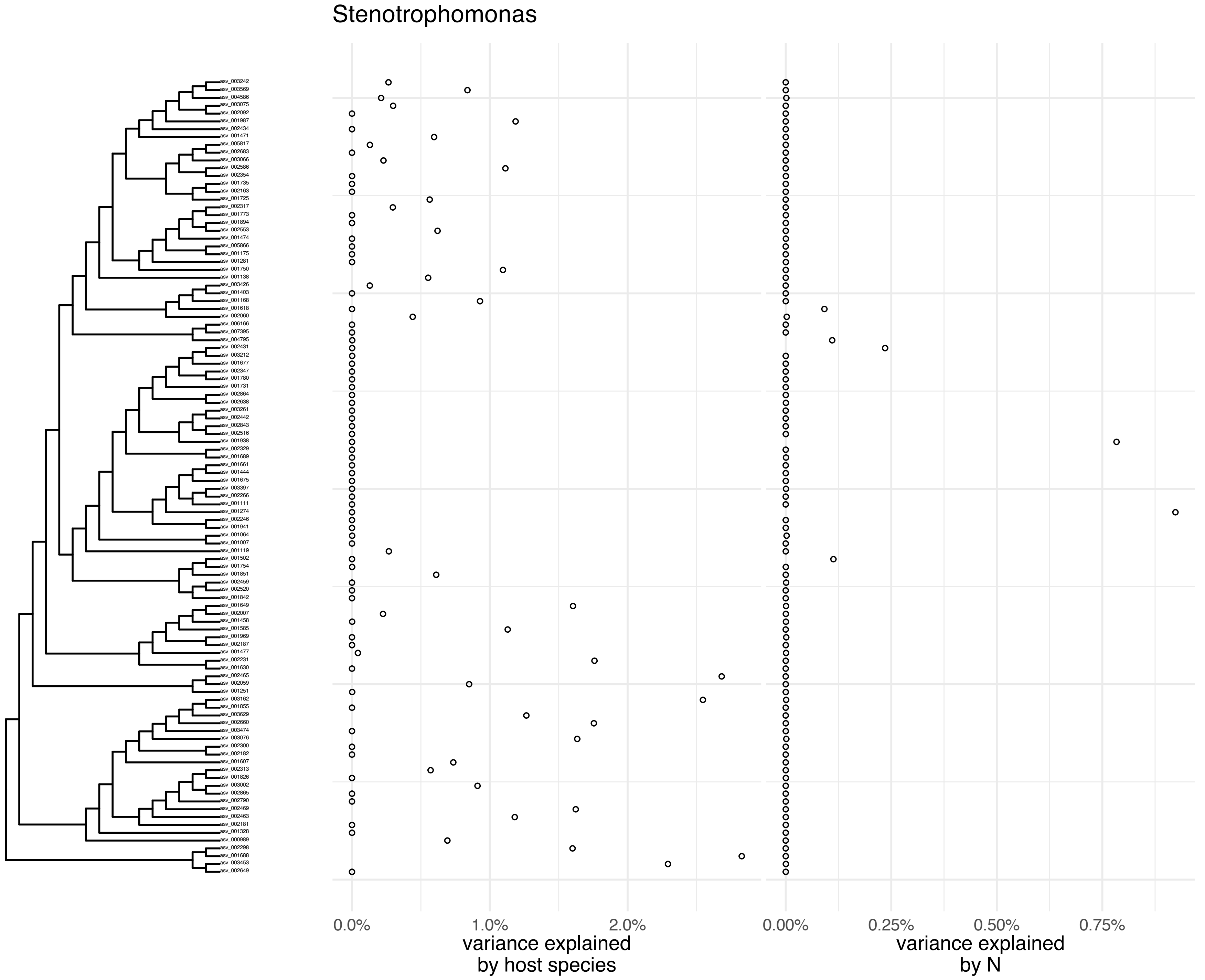
Stenotrophomonas
0.0%
1.0%
2.0%
variance explained
by host species
0.00%
0.25%
0.50%
0.75%
variance explained
by N

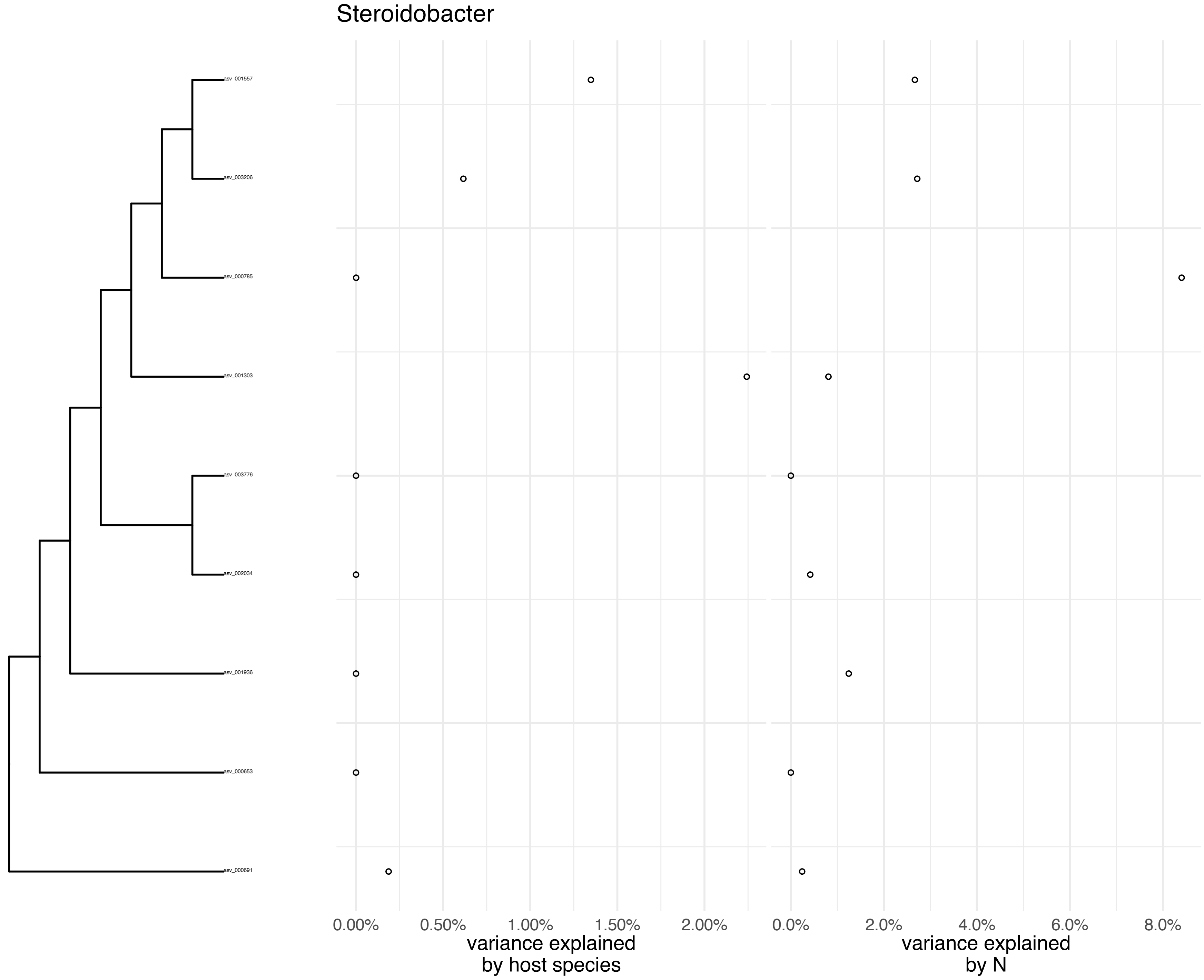
Steroidobacter
asv_001557
asv_003206
asv_000785
asv_001303
asv_003776
asv_002034
asv_001936
asv_000653
asv_000691
0.00%
0.50%
1.00%
1.50%
2.00%
variance explained
by host species
0.0%
2.0%
4.0%
6.0%
8.0%
variance explained
by N

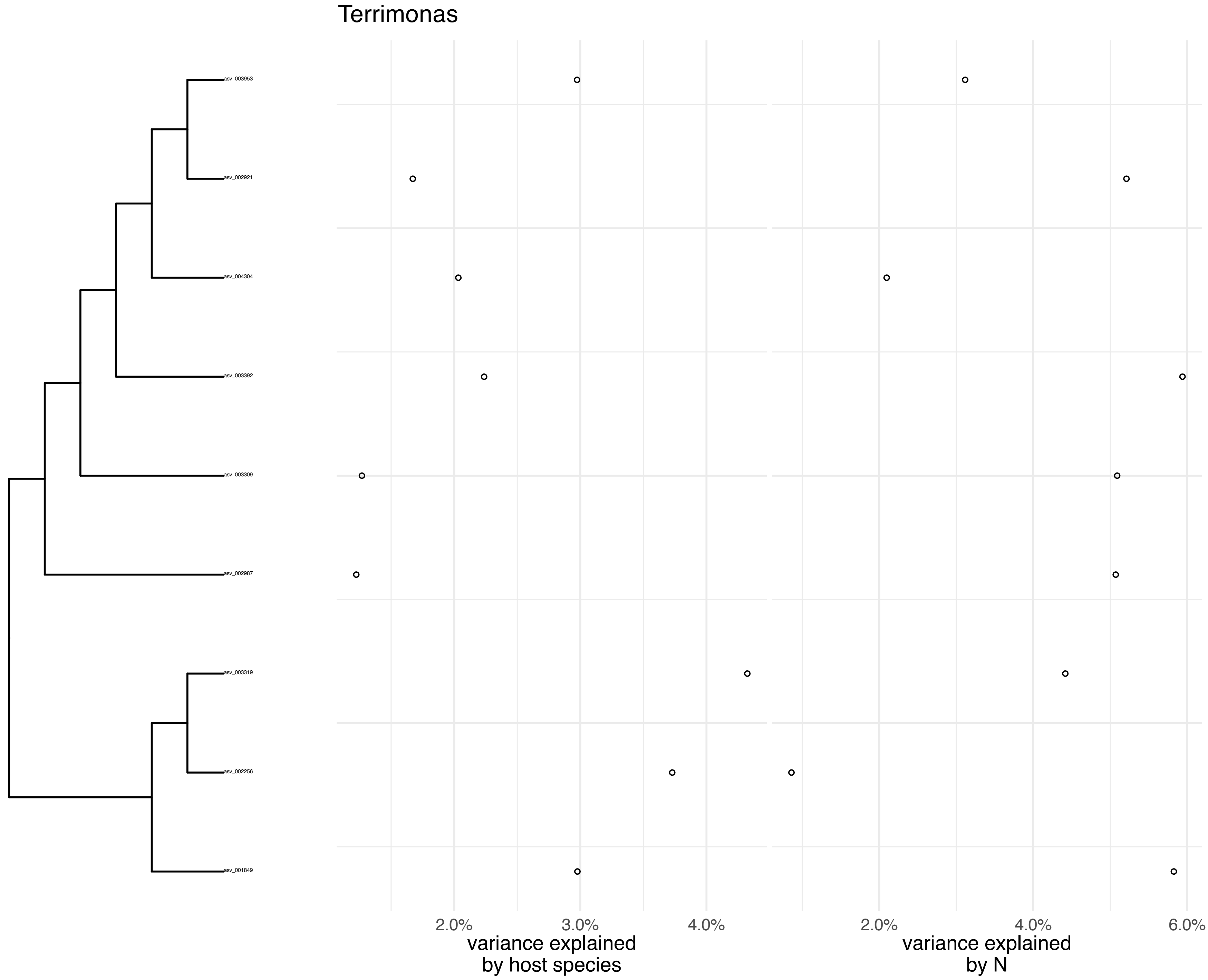
Terrimonas
asv_003953
asv_002921
asv_004304
asv_003392
asv_003309
asv_002987
asv_003319
asv_002256
asv_001849
2.0%
3.0%
4.0%
variance explained
by host species
2.0%
4.0%
6.0%
variance explained
by N

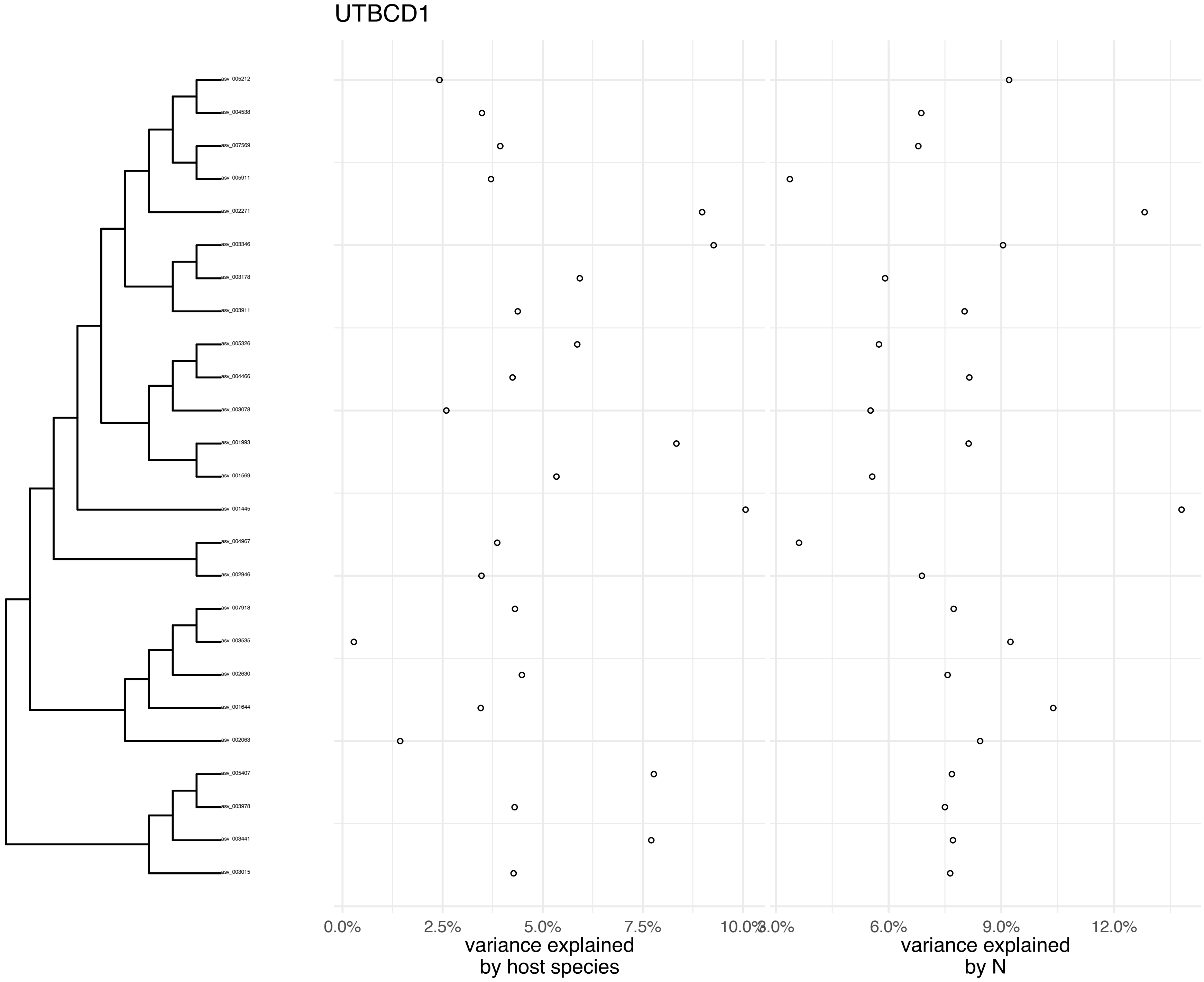
UTBCD1
asv_005212
asv_004538
asv_007569
asv_005911
asv_002271
asv_003346
asv_003178
asv_003911
asv_005326
asv_004466
asv_003078
asv_001993
asv_001569
asv_001445
asv_004967
asv_002946
asv_007918
asv_003535
asv_002630
asv_001644
asv_002063
asv_005407
asv_003978
asv_003441
asv_003015
0.0%
2.5%
5.0%
7.5%
10.0%
variance explained
by host species
3.0%
6.0%
9.0%
12.0%
variance explained
by N

Variovorax

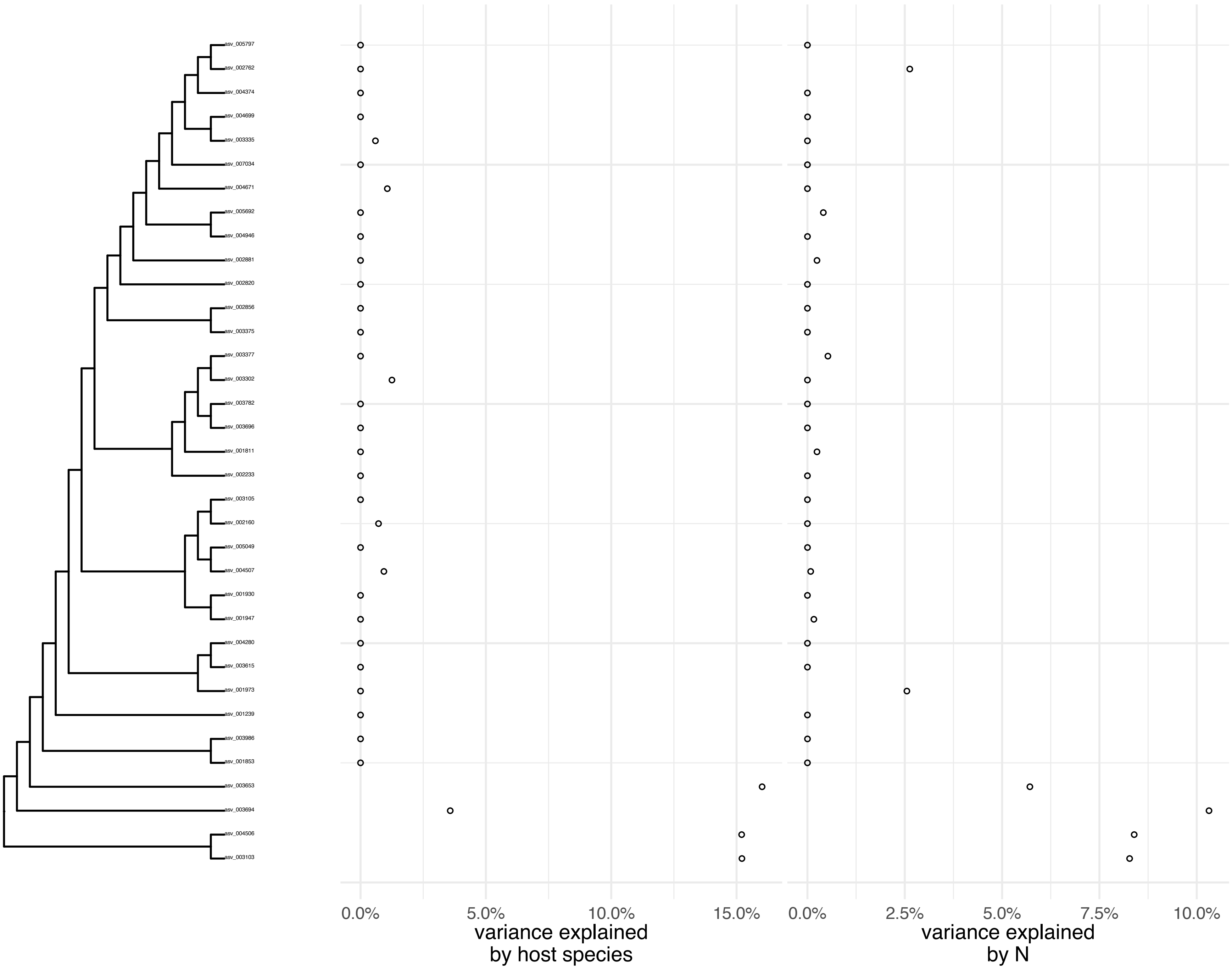

asv_005797
asv_002762
asv_004374
asv_004699
asv_003335
asv_007034
asv_004671
asv_005692
asv_004946
asv_002881
asv_002820
asv_002856
asv_003375
asv_003377
asv_003302
asv_003782
asv_003696
asv_001811
asv_002233
asv_003105
asv_002160
asv_005049
asv_004507
asv_001930
asv_001947
asv_004280
asv_003615
asv_001973
asv_001239
asv_003986
asv_001853
asv_003653
asv_003694
asv_004506
asv_003103
0.0%
5.0%
10.0%
15.0%
variance explained by host species
0.0%
2.5%
5.0%
7.5%
10.0%
variance explained by N

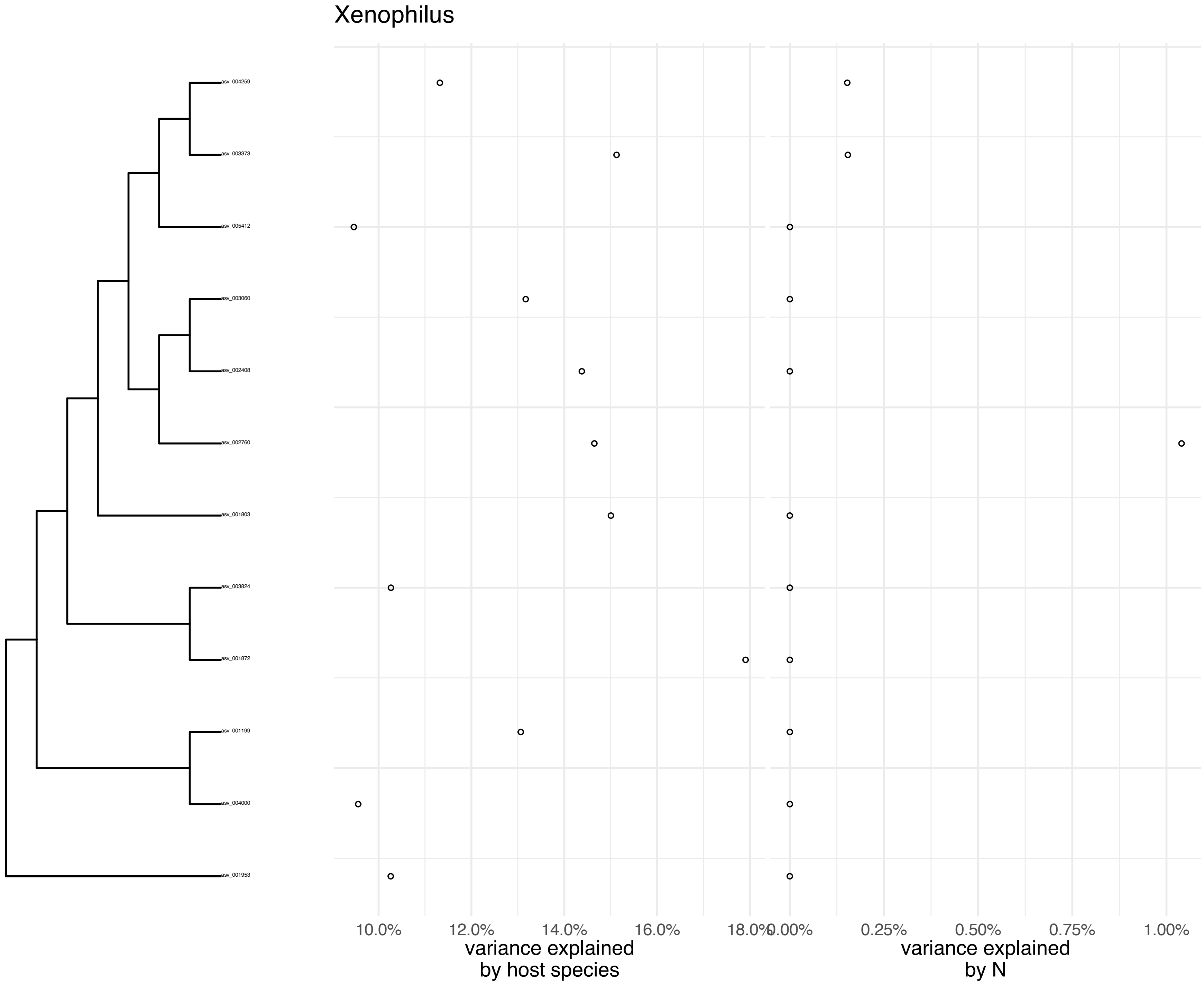
Xenophilus
asv_004259
asv_003373
asv_005412
asv_003060
asv_002408
asv_002760
asv_001803
asv_003824
asv_001872
asv_001199
asv_004000
asv_001953
10.0%
12.0%
14.0%
16.0%
18.0%
0.00%
0.25%
0.50%
0.75%
1.00%
variance explained by host species
variance explained by N

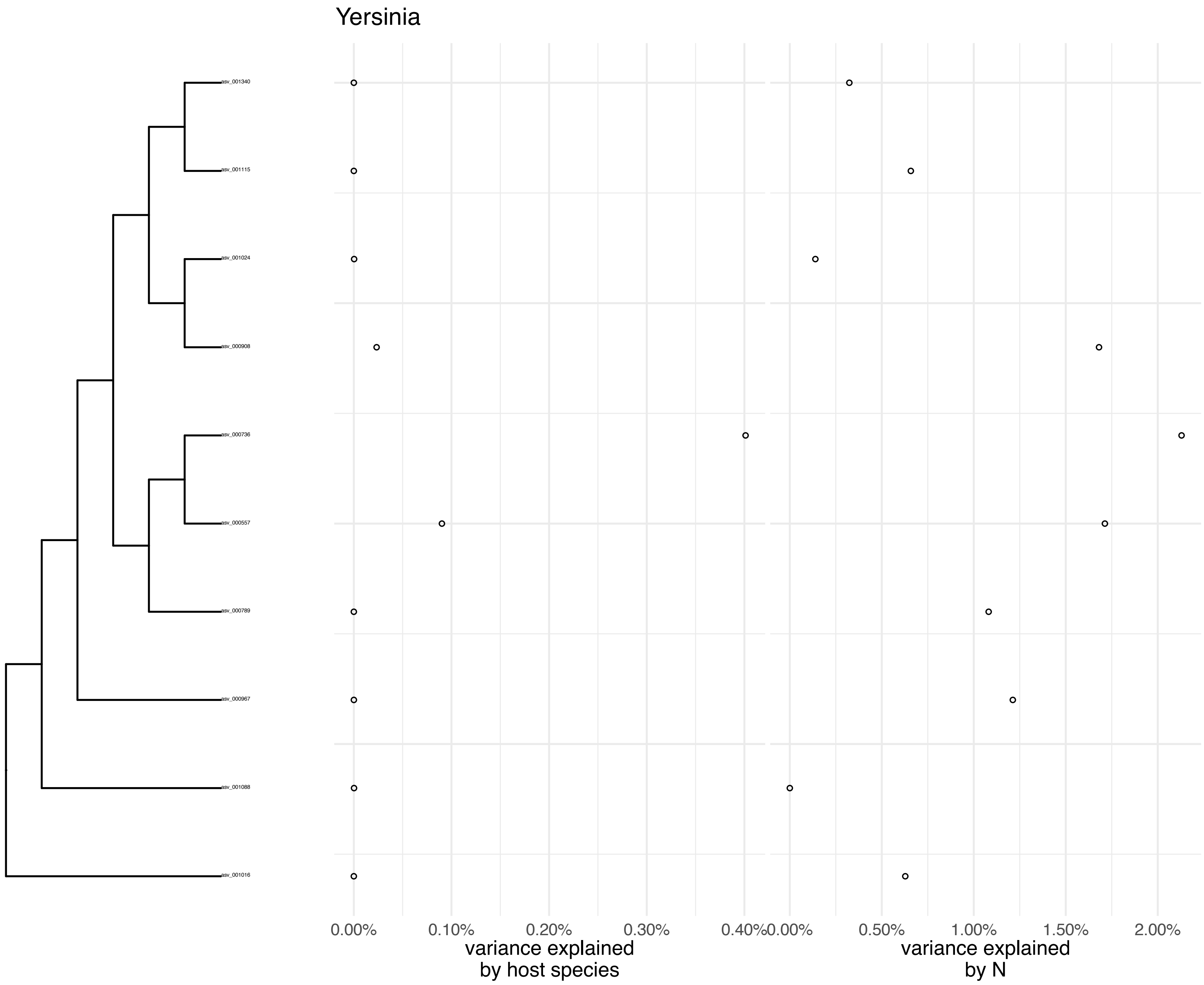

Yersinia
asv_001340
asv_001115
asv_001024
asv_000908
asv_000736
asv_000557
asv_000789
asv_000967
asv_001088
asv_001016
0.00%
0.10%
0.20%
0.30%
0.40%
variance explained by host species
0.00%
0.50%
1.00%
1.50%
2.00%
variance explained by N
